# High-speed, scanned laser structuring of multi-layered eco/bioresorbable materials for advanced electronic systems

**DOI:** 10.1038/s41467-022-34173-0

**Published:** 2022-10-31

**Authors:** Quansan Yang, Ziying Hu, Min-Ho Seo, Yameng Xu, Ying Yan, Yen-Hao Hsu, Jaime Berkovich, Kwonjae Lee, Tzu-Li Liu, Samantha McDonald, Haolin Nie, Hannah Oh, Mingzheng Wu, Jin-Tae Kim, Stephen A. Miller, Ying Jia, Serkan Butun, Wubin Bai, Hexia Guo, Junhwan Choi, Anthony Banks, Wilson Z. Ray, Yevgenia Kozorovitskiy, Matthew L. Becker, Mitchell A. Pet, Matthew R. MacEwan, Jan-Kai Chang, Heling Wang, Yonggang Huang, John A. Rogers

**Affiliations:** 1 Querrey Simpson Institute for Bioelectronics, Northwestern University, Evanston, IL 60208 USA; 2 Department of Mechanical Engineering, Northwestern University, Evanston, IL 60208 USA; 3 Department of Materials Science and Engineering, Northwestern University, Evanston, IL 60208 USA; 4 School of Biomedical Convergence Engineering, College of Information & Biomedical Engineering, Pusan National University, Pusan, 46241 Republic of Korea; 5 The Institute of Materials Science and Engineering, Washington University in St. Louis, St. Louis, MO 63130 USA; 6 Department of Neurosurgery, Washington University School of Medicine in St. Louis, St. Louis, MO 63130 USA; 7 Department of Chemistry, Duke University, Durham, NC 27708 USA; 8 Department of Biological Sciences, Northwestern University, Evanston, IL 60208 USA; 9 Department of Biomedical Engineering, Northwestern University, Evanston, IL 60208 USA; 10 Department of Neurobiology, Northwestern University, Evanston, IL 60208 USA; 11 Laser and Electronics Design Core Facility, Northwestern University, Evanston, IL 60208 USA; 12 Micro/Nano Fabrication Facility, Northwestern University, Evanston, IL 60208 USA; 13 Department of Applied Physical Sciences, University of North Carolina at Chapel Hill, Chapel Hill, NC 27599 USA; 14 Developmental Therapeutics Core, Northwestern University, Evanston, IL 60208 USA; 15 Department of Biomedical Engineering and Orthopaedic Surgery, Duke University, Durham, NC 27708 USA; 16 Division of Plastic and Reconstructive Surgery, Washington University School of Medicine in St. Louis, St. Louis, MO 63130 USA; 17 Wearifi Inc., Evanston, IL 60201 USA; 18 Center for Flexible Electronics Technology, Tsinghua University, Beijing, 100084 China; 19 Zhejiang Tsinghua Institute of Flexible Electronics Technology, Jiaxing, 314000 China; 20 Department of Civil and Environmental Engineering, Northwestern University, Evanston, IL 60208 USA; 21 Department of Neurological Surgery, Feinberg School of Medicine, Northwestern University, Chicago, IL 60611 USA

**Keywords:** Design, synthesis and processing, Biomedical engineering, Surface patterning, Sensors and biosensors, Lithography

## Abstract

Physically transient forms of electronics enable unique classes of technologies, ranging from biomedical implants that disappear through processes of bioresorption after serving a clinical need to internet-of-things devices that harmlessly dissolve into the environment following a relevant period of use. Here, we develop a sustainable manufacturing pathway, based on ultrafast pulsed laser ablation, that can support high-volume, cost-effective manipulation of a diverse collection of organic and inorganic materials, each designed to degrade by hydrolysis or enzymatic activity, into patterned, multi-layered architectures with high resolution and accurate overlay registration. The technology can operate in patterning, thinning and/or cutting modes with (ultra)thin eco/bioresorbable materials of different types of semiconductors, dielectrics, and conductors on flexible substrates. Component-level demonstrations span passive and active devices, including diodes and field-effect transistors. Patterning these devices into interconnected layouts yields functional systems, as illustrated in examples that range from wireless implants as monitors of neural and cardiac activity, to thermal probes of microvascular flow, and multi-electrode arrays for biopotential sensing. These advances create important processing options for eco/bioresorbable materials and associated electronic systems, with immediate applicability across nearly all types of bioelectronic studies.

## Abstract (web summary)

Designing and manufacturing eco/bioresorbable electronic systems remains a challenge. The authors introduce a picosecond-pulsed laser-based scheme that exploits controlled patterning, thinning, and/or cutting to manipulate multilayers of eco/bioresorbable materials for a wide range of advanced electronic systems.

## Introduction

Eco/bioresorbable electronic systems have a broad range of applications, from temporary biomedical implants to low-cost consumer gadgetry to internet-of-things (IoT) sensors, where processes of resorption eliminate risks and costs associated with surgical extraction and management of electronic waste^1–5^. High-speed scalable manufacturing of such classes of electronics is a requirement for their widespread deployment in medical (e.g., millions of registered cardiac pacemakers^6^ and brain monitors^7^ worldwide) or IoT (over 20 billion worldwide^8^, including 2 billion radio-frequency identification (RFID) tags specifically^9^) devices. Published examples rely on multi-step processes that combine photolithography-based methods in microfabrication^10–17^ with transfer printing, designed to avoid chemical or thermal degradation of the eco/bioresorbable constituent materials. These schemes work well for demonstrations but might present challenges in cost-effective, mass production^18^. Alternatives that exploit solution-based additive printing of functional inks^19–23^ can form simple, low-resolution passive components, but do not apply effectively to high-performance eco/bioresorbable semiconductors such as monocrystalline silicon. An ideal fabrication methodology must offer (1) compatibility with the full range of eco/bioresorbable materials, including conductors, semiconductors, dielectrics, and polymer substrates, (2) capabilities in precise dimensional control (thicknesses and lateral features) and accurate overlay registration, (3) straightforward routes to multi-layered, multi-material device architectures with functions to address various application scenarios.

Pulsed laser ablation methods exploit well-controlled light-matter interactions for surface patterning^24^, with various demonstrated uses in manufacturing^25^. The accuracy and precision of lateral patterning and thickness reduction depend not only on the properties of the materials but on key laser parameters, especially pulse duration and wavelength^26^. Ultrashort pulsed lasers (less than 10 ps) are advantageous because the ablation process occurs on timescales sufficiently short to limit thermal diffusion and the associated spread of the heat-affected zone^27–29^. Wavelengths ranging from ultraviolet (UV) to near infrared (NIR) for ultrashort pulsed lasers allow the ablation rates, as well as surface morphologies, to be tuned due to the wavelength-dependent reflectivity and absorption of the materials^30,31^. The mechanism of ultrafast laser ablation involves explosive vaporization and melt cavitation^32^. Direct sublimation to the gas phase and/or ionization into dense plasmas likely occur in these processes^33,34^. Dedicated systems exist for manufacturing interconnect traces in flexible circuit boards^35–37^ and components of certain classes of thin-film sensors^25,38–40^. These approaches are, however, most well developed for structures with modest resolution (lateral and vertical resolution of over 20 µm and 1 µm, respectively) and for single functional layers; they have not been applied to semiconductor materials generally, nor to eco/bioresorbable materials specifically.

This paper introduces a high-speed, scanned, picosecond-pulsed laser ablation approach, which, combined with physical lamination and/or transfer printing, serves as a versatile method for fabricating advanced multi-layered eco/bioresorbable electronic systems through careful selection of laser parameters. One appeal of this scheme is in its alignment with current manufacturing and prototyping schemes for conventional flexible printed circuit boards (PCBs)^35,37,38,40,41^. When implemented in a repetitive fashion using materials delivered to a substrate in sequence by physical lamination, transfer printing or conventional deposition or growth techniques, this methodology can yield state-of-the-art eco/bioresorbable electronic components, including complementary metal-oxide-semiconductor (CMOS) devices, through several basic operational modes. Here, ablation removes and patterns nano/micromembranes of constituent materials in an efficient, controlled manner via picosecond-pulsed light-matter interactions^42,43^, all in a fast, dry process with minimal thermal load. The process can accurately control the thicknesses (to within ~35 nm) and lateral dimensions (down to ~5 µm by overlapping scans) of features formed using these materials, in multi-layered configurations with good overlay registration (2–3 µm). A collection of system-level demonstrations in sensors of pressure, physiological parameters, thermal characteristics, flow properties, biopotential signals, and mechanical strains across multiple organ systems in live animal models illustrate important applications. Specific examples include wireless, implantable monitors for physiological behaviors in rat models, thermal probes for microcapillary flow sensing in transplanted tissues/organs in porcine models, flexible multi-electrode arrays for biopotential sensing on the cerebral cortex in mouse models, and wireless systems for tracking principal and shear strains on the epicardial surfaces in ovine models. These collective results highlight the broad spectrum of possibilities enabled by this fabrication technology.

## Results and discussion

### Laser ablation approaches for eco/bioresorbable electronics

**Fig. 1:**
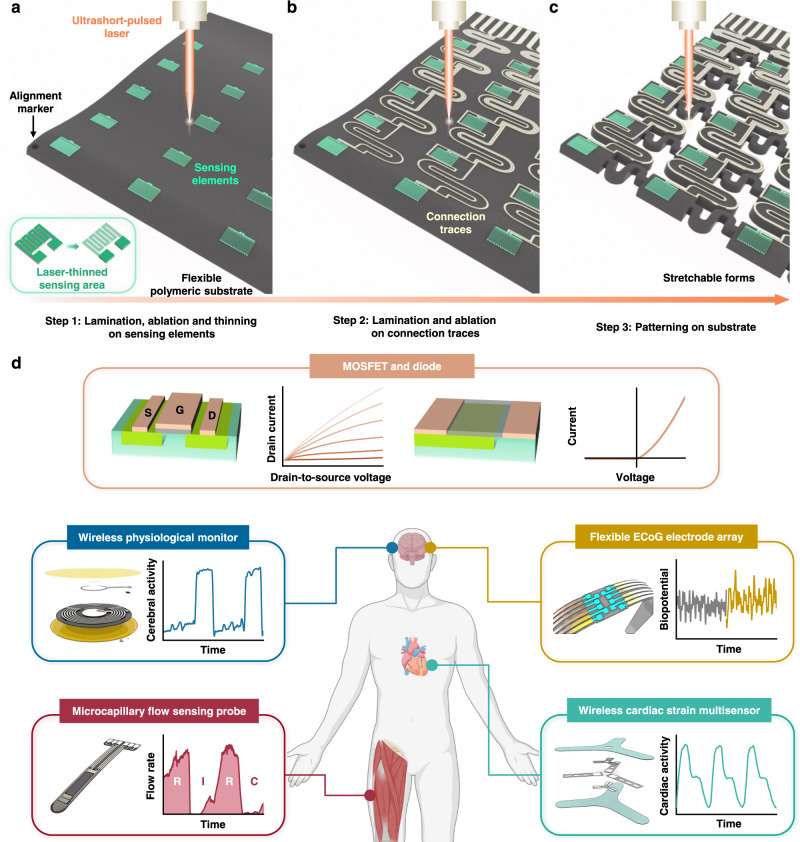
Laser ablation processes for fabricating advanced multi-layered bioresorbable electronics. Laser ablation fabrication procedures for bioresorbable electronics, including patterning of sensing elements and structuring of alignment markers (**a**), patterning of connection traces (**b**), and structuring substrates into stretchable forms (**c**). Inset: laser thinning process on the sensing elements in **a**. **d** Illustrations of a representative collection of bioresorbable electronic systems fabricated using these procedures, such as Si-based semiconductor devices and a series of sensors of pressure, cardiopulmonary activity, thermal transport, fluid flow, biopotential, and mechanical strain, for various organs and biomedical applications. System examples include wireless monitors for physiological parameters in tissue cavities, probes for microcapillary flow sensing in transplanted tissues/organs, flexible Si-based electrode arrays for biopotential recordings, and wireless systems for measuring principal and shear strains on the epicardial surface.

The picosecond-pulsed laser ablation approaches described here enable high-resolution (1) patterning of films through the removal of material from defined regions, (2) thinning of films through reductions in thickness in patterned geometries, with minimal damage to underlying layers, and (3) cutting through films and their supporting substrates. A broad range of well-established bioresorbable materials, ranging from films, membranes and/or foils of conductors, semiconductors, dielectrics to encapsulants and substrates prepared through lamination, transfer printing and/or deposition/growth, can be processed using these schemes. Repetitive application of preparation-ablation cycles can yield multi-layered structures for advanced electronic designs. This scheme offers a simple alternative to conventional cleanroom fabrication methods and photolithography techniques, and it avoids the need for any wet processing steps, including those that use aqueous solutions for etching, developing, cleaning, or washing, thereby eliminating any potential for degradation of the eco/bioresorbable materials, most of which are water-soluble (Supplementary Note 1 and Supplementary Fig. 1). As a representative example, Fig. 1a–c and Supplementary Fig. 2 illustrate the procedures for forming a multi-layered bioresorbable device that consists of an array of functional elements on a poly (lactic acid) (PLA) substrate, where monocrystalline Si micromembranes (Si MMs) serve as sensing elements and patterned Mg films act as electrical interconnections. In the 1st cycle, the process defines cuts (diameter: 1.5 mm) through the substrate as alignment marks for subsequent cycles of patterning of various material structures from uniform films or foils. The final step cuts the substrate into a serpentine structure to provide some effective level of stretchability, according to the principles of soft electronics^44,45^.

Supplementary Table 1 and Supplementary Fig. 3 summarize key features of this processing sequence. First, the procedures are entirely dry and they involve minimum heat generation resulting from ultrashort light-matter interactions associated with the use of a picosecond-pulsed laser. These characteristics minimize thermally induced degradation or damage to the constituent materials. Second, by careful selection of the parameters for ablation, material removal can occur in a well-controlled manner, with high lateral resolution (5 µm) and good overlay registration (2–3 µm). Third, complex single and multi-layered device structures are possible, including those that incorporate device-grade monocrystalline silicon, such as metal-oxide-semiconductor field-effect transistors (MOSFETs) and diodes. A collection of systems for measuring pressure, physiological parameters, thermal characteristics, flow properties, biopotential signals, and mechanical strains across various organ systems appears in Fig. 1d. Specific embodiments range from wireless monitors for physiological behaviors in tissue cavities, probes for microcapillary flow sensing in transplanted tissues/organs, flexible Si-based electrode arrays for biopotential sensing on tissue surfaces, and wireless systems for tracking principal and shear strains on the epicardium.

### Characterization of the ablation process

**Fig. 2:**
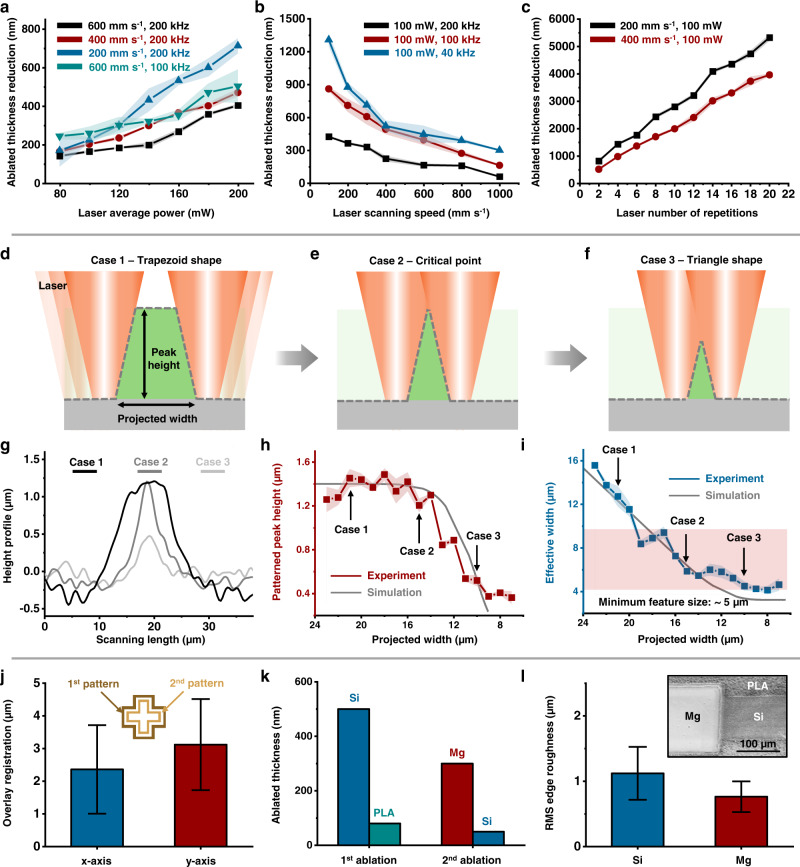
Key characteristics of the ablation process. Controlled reductions in thicknesses of monocrystalline Si MMs achieved by ablation, and their dependence on ablation parameters, including average power (**a**), scanning speed (**b**), frequency (**b**), and the number of repetitions (**c**) at fixed grid distance (7 µm) and grid mode (XY-parallel). The shaded areas denote the standard deviation. Schematic illustration of cross-sectional profiles of ribbon-shaped structures of monocrystalline Si MM formed by ablation; a trapezoid shape with a titled edge (projected width larger than the laser spot diameter *D*_*laser*_; case 1; **d**), a triangle shape at the critical point (projected width similar to D_laser_; case 2; **e**), and a proportionally reduced triangle shape (projected width smaller than D_laser_; case 3; **f**). **g** Experimentally measured profiles for these three cases. Peak height (**h**) and effective width (**i**) as functions of projected width. The effective width corresponds to the integrated cross-sectional area divided by the peak height. The minimum feature size is ~5 µm. The shaded areas denote the standard deviation. **j** Alignment accuracy in the *x*- and *y*-axis is 2.7 ± 1.3 and 3.1 ± 1.2 µm, respectively. **k** Minimized damage to underlying materials following laser ablation processing of the top layer. Ablating a 500-nm-thick top Si MM layer leads to an ablated thickness of ~80 nm for the underlying PLA layer (original thickness: 50 µm); ablating a 300-nm-thick top Mg layer leads to an ablated thickness of ~50 nm for the underlying Si layer (original thickness: 500 nm). **l**, Root-mean-square (RMS) line edge roughness for Si (thickness: 2 µm; width: 100 µm) and Mg (thickness: 500 nm; width: 100 µm) ribbons patterned on PLA substrates (thickness: 50 µm) are 1.1 ± 0.4 µm and 0.8 ± 0.2 µm, respectively. Insets: SEM images (titled angle: 45°) of a bi-layer Mg/Si structure (thickness: 300 nm for top Mg and 2 µm for bottom Si) on a PLA substrate (thickness: 50 µm).

Figure 2a–c and Supplementary Figs. 4, 5 present examples of patterned reductions in the thicknesses of micromembranes of device-grade silicon and thin films of Mg through control of key laser parameters such as average power (*P*; 80–200 mW; Fig. 2a), scanning speed (*v*; 200–600 mm s^−1^; Fig. 2b), frequency (*f*; 40–200 kHz; Fig. 2b), and the number of repetitions (*n*; 2–20; Fig. 2c) experimentally and theoretically, at fixed grid distance (*d*; 7 µm) and grid mode (XY-parallel) as described in detail in Supplementary Fig. 6. The thickness reduction increases monotonically with average power and number of repeats and decreases with scanning speed and frequency. The peak power increases while the frequency decreases when the average power remains unchanged. A simple, empirical model assumes that the intensity of the focused laser beam follows a Gaussian profile with a diameter *d*_*0*_ and that the maximum thickness reduction by a single laser pulse (*D*_max_) occurs at the circular central region, increasing with the pulse energy *W*, where *W = P/f*, and with a magnitude that depends on material properties such as the heat capacity, density, vaporization temperature, and heat of vaporization^46^ and on the laser parameters such as the wavelength^30^, the pulse duration^47^, the peak intensity^48^, and others. An empirical formula fitted from the experimental results gives *D*_*max*_ = *α* × *W*^*β*^, where *α* and *β* depend on material properties (e.g., *α* = 1 × 10^5^ nm mJ^−1/β^ and *β* = 1.9 for monocrystalline Si; *α* = 4 × 10^3^ nm mJ^−1/β^ and *β* = 1.2 for Mg). Descriptions of the nature of these nonlinearities can be found elsewhere^49,50^. The ablated thickness decreases with the distance to the center of the circular region (*r*) according to a Gaussian function^51^ $D={D}_{{{\max }}}{{\exp }}\left(-k{r}^{2}{d}_{0}^{-2}\right)$, where *k* is an empirical parameter that defines the thickness profile, and is found to take a value of ~50 for the experimental results presented here. The total thickness ablated by multiple pulses is a summation of that by an individual pulse. The experimental results agree well with those based on theoretical models of the ablation process. The minimum reduction in thickness is ~35 nm for the laser system and the Si MMs examined here (Supplementary Fig. 7). Specific parameters depend on material properties, as expected. Additionally, numerical simulation in Supplementary Fig. 8 reveals that the length scale for a body heat flux with the duration used in our system (~1 ps) is ~5 nm, which is negligible compared to the lateral patterning resolution (~5 µm). Length scale values with different durations appear in Supplementary Fig. 8c. The results highlight that the ultrashort pulsed laser efficiently minimizes the thermal diffusion zone adjacent to the edges of patterned features.

The lateral resolution of ~5 µm follows from the use of a 15-µm-diameter, Gaussian laser spot (Supplementary Fig. 9). Overlapping scans provide additional capabilities in resolution, as illustrated in the case of two sequential scans at various separations, referred to as projected widths, as in Fig. 2d–f. The Si ribbons that result from this process possess trapezoidal cross-sectional dimensions (case 1 in Fig. 2d) with projected widths larger than the laser spot diameter. Reducing the projected width to values comparable to this diameter (case 2 in Fig. 2e) yields a triangular cross-section. Further reductions decrease the size of the triangle proportionally, leading to a reduced cross-sectional area (case 3 in Fig. 2f). Figure 2g summarizes experimentally measured cross-sectional profiles for these three scenarios, consistent with simulation results (Supplementary Fig. 10). Figure 2h, i presents the peak heights and effective widths (integrated cross-sectional area divided by the peak height) of the patterned Si ribbons, respectively, as a function of projected width determined experimentally and theoretically. The peak height values in cases 1 and 2 are similar; however, the values decrease in case 3 with the reduction in the projected width. The effective width, which defines the width of the ribbons, decreases continuously with the projected width, to a minimum value of ~5 µm in case 3. Optical, scanning electron microscope (SEM), and atomic force microscope (AFM) images from the narrowest monocrystalline Si ribbons (width: ~4–5 µm) on a PLA substrate appear in Supplementary Fig. 11. Similar scenarios and minimum feature sizes occur in patterning trench shapes, shown in Supplementary Fig. 12. These and other important capabilities of the process rely on accurate registration through multiple cycles of patterning. The results in Fig. 2j and Supplementary Fig. 13 indicate that the overlay registration along the *x*-axis and *y*-axis is 2.7 ± 1.3 and 3.1 ± 1.2 µm, respectively.

This controlled ablation process extends to the ability to pattern or to reduce the thicknesses of surface layers with minimum damage to the underlying materials. Two examples illustrate this capability—complete removal of a top layer of Si (thickness: 500 nm) on PLA (initial thickness: 50 µm) and of Mg (thickness: 300 nm) on Si (initial thickness: 500 nm), as summarized in Fig. 2k. Characterization by 3D laser scanning microscopy indicates that the depth of damage to the substrate in both cases (depth for PLA: ~80 nm; depth for Si: ~50 nm) is small compared with the initial thicknesses of the overlying layers. Related surface-selective processing can be used to form micro-textures that define the morphology and control related properties such as wettability, as illustrated in Supplementary Fig. 14. The line edge roughness of patterned ribbons of Si (thickness: 2 µm; width: 100 µm) and Mg (thickness: 500 nm; width: 100 µm) on a PLA substrate (thickness: 50 µm) appears in Fig. 2l. The root-mean-square (RMS) values are 1.1 ± 0.4 µm for Si and 0.8 ± 0.2 µm for Mg, respectively. Top and side views of the surface morphologies of Si and Mg ribbons and bi-layer Si/Mg structures appear in SEM images in Supplementary Fig. 15 and Inset of Fig. 2l. The process also allows the formation of large-area arrays. An array of thermistors (materials: Mg/PLA; thickness: 0.3/50 µm) serves as a representative example, as shown in Supplementary Fig. 16, which illustrates large-area fabrication (dimension: 85 × 55 mm) of many (45 in a 9 × 5 configuration for this example) devices on a PLA substrate patterned into a stretchable, serpentine mesh. Related ablation processes apply at the industrial scale, with patterning speeds that depend on the laser scanning speed, the patterns of these scans, and the number of repetitions. For monocrystalline Si, as an example of interest to the present work, the patterning speed is ~ 10–30 s cm^−2^ with the laser system used here when the scanning speed, grid distance, and the number of repetitions are 1000 mm s^−1^, 7 µm, and 1, respectively.

### Processing by thickness reduction for wireless bioresorbable physiological monitors

**Fig. 3:**
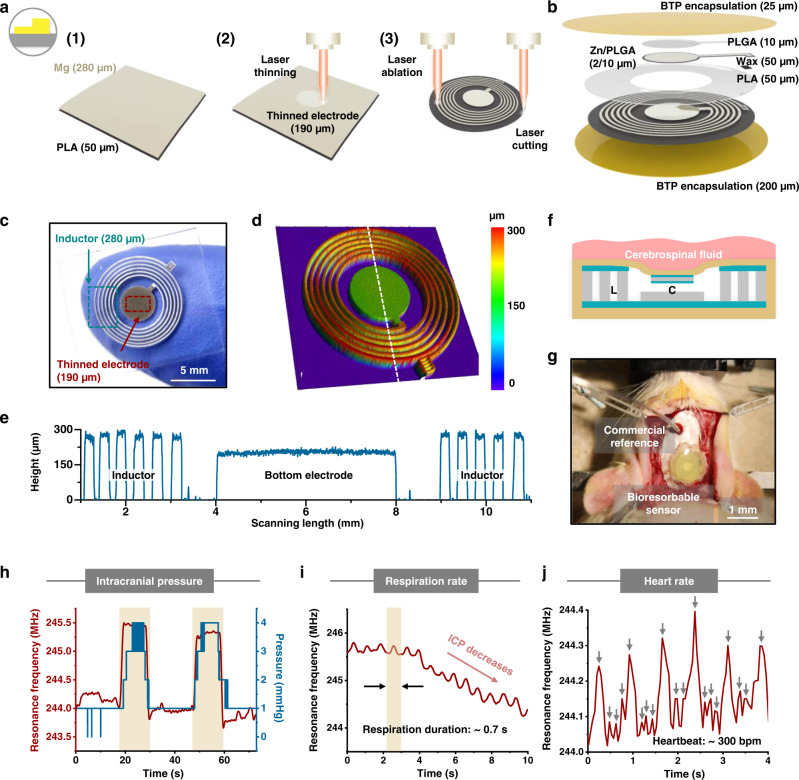
Strategy for thinning material structures used in wireless, bioresorbable physiological monitors. Procedures. **a** Schematic illustration of the process applied to an inductor with a thinned bottom electrode, as a key component in such a device. The procedures consist of (1) lamination of a uniform Mg layer (thickness: 280 µm) on a PLA substrate (thickness: 50 µm) using the BTP interfacial adhesive, (2) thinning of the Mg layer by ablation to reduce the thickness of the central circular area (diameter: 4 mm) from 280 to 190 µm for the bottom electrode, and (3) ablation of the surrounding Mg to define a helical coil structure and to cut the PLA substrate into the outline of the device. **b** Schematic illustration of devices formed in this manner. **c** Photograph of an inductor with thinned bottom electrode on a PLA substrate. **d** Height profile of the key component determined by 3D confocal optical microscopy. **e**, Quantitative results along the white dashed line in **d**. **f** Working principle of such a device. The parallel capacitor, with a gap between the top and bottom electrodes defined by the thinning process. Changes in capacitance result from changes in this distance induced by variations in pressure between the surrounding cerebrospinal fluid and the air cavity. This LC-type device converts the capacitance change into changes in resonance frequency properties, as the basis for passive, wireless sensing. In vivo acute evaluations in a rat model. **g** Photograph of a bioresorbable physiological monitoring device mounted above an opened craniotomy, with a standard clinical ICP monitor as a reference. Acute recordings of physiological parameters, including ICP (**h**; red: bioresorbable device; blue: commercial reference), respiration rate (**i**), and heart rate (**j**) in the rat model.

Tracking internal pressures and other physiological parameters forms the basis of many operations in patient monitoring, often needed only for periods of time that coincide with a temporary condition such as recovery from a surgical operation^10,52,53^. An example bioresorbable device that can be useful in this context consists of a circular inductor with a thinned bottom electrode (material: Mg; thickness: 280 µm for coil and ~190 µm for bottom electrode; substrate: 50-µm-thick PLA), an annular spacer (material: PLA; thickness: 50 µm), a top electrode (material: PLGA/Zn/PLGA; thickness: 10/2/10 µm), and top and bottom layers of bioresorbable thermoplastic polymer (BTP; synthesis process in methods section) for encapsulation (thickness: 25 µm for top and 200 µm for bottom). Figure 3a illustrates the procedures for the inductor, which incorporates a central thinned bottom electrode as a critical component of the device. Bonding a Mg foil (thickness: 280 µm) on a PLA substrate (thickness: 50 µm) with BTP as an adhesive (Supplementary Fig. 17a) completes the preparation of the material stack, shown in step (1). The ablation process reduces the thickness of the central circular area (diameter: 4 mm) from 280 to 190 µm for the bottom electrode, shown in step (2). Additional cycles of ablation pattern the surrounding Mg into a helical coil as an inductor and cut the PLA substrate to define the device outline, shown in step (3). Assembling these components and sealing the edge between the top and bottom layers of BTP completes the fabrication, shown in Fig. 3b. A photograph of the resulting inductor appears in Fig. 3c. Figure 3d highlights the height profile of the thinned bottom electrode, as characterized by 3D confocal optical microscopy. The line cut (white dashed) in Fig. 3d features the thickness variation (190 µm in the bottom electrode area and 280 µm in the inductor area), shown in Fig. 3e. The average surface roughness (Ra) and root-mean-square surface roughness (RMS) are ~ 4.4 and 5.6 µm, respectively, for the bottom electrode. The quality factor (Q factor) of the inductor is larger than 70 (Supplementary Fig. 17b). Inductors with sub-mm scale (diameter: ~1 mm; number of turns: 4; line width: 90 µm; spacing: 20 µm) dimensions are also possible, as shown in Supplementary Fig. 17c, d.

The schematic cross-sectional illustration in Fig. 3f explains the working principle of the device when used as a passive, wireless pressure sensor. The parallel-plate capacitor formed by the air gap between the top and bottom electrodes responds to changes in their separation due to changes in the pressure of surrounding biofluids. The result shifts the resonance frequency of the inductor-capacitor (LC) circuit, which can be detected wirelessly by an external readout coil and vector network analyzer. In vivo evaluations in a rat model demonstrate the operation. Figure 3g shows the device mounted above an opened craniotomy defect. A standard clinical intracranial pressure (ICP) monitor inserted through a second craniotomy defect serves as a pressure reference. Figure 3h–j highlights in vivo acute recordings. Compressing and releasing the flank of the rat cause variations in ICP, as detected by the device (marked red) in Fig. 3h. The results align with those of the commercial reference (marked blue). The data indicate a sensitivity of ~500 kHz mmHg^−1^. Additionally, physiological parameters such as respiration rate (duration: ~ 0.7 s) and heart rate (duration: ~ 0.2 s), shown in Fig. 3i, j, can also be tracked in parallel with the ICP measurement. Another demonstration of thickness reduction for resistive-type bioresorbable devices appears in Supplementary Note 2 and Supplementary Fig. 18. The same methods can process not only Mg, but also other eco/bioresorbable metals, such as Zn and Mo. Supplementary Fig. 19 presents results of precise patterning of Zn and Mo on CA substrates into flexible sensing array shapes. Besides eco/bioresorbable materials, representative non-eco/bioresorbable materials, such as polyimide (PI) and polydimethylsiloxane (PDMS), can also be laser-thinned and laser-ablated, as shown in Supplementary Fig. 20.

### Processing of bioresorbable microvascular flow sensing probes

**Fig. 4:**
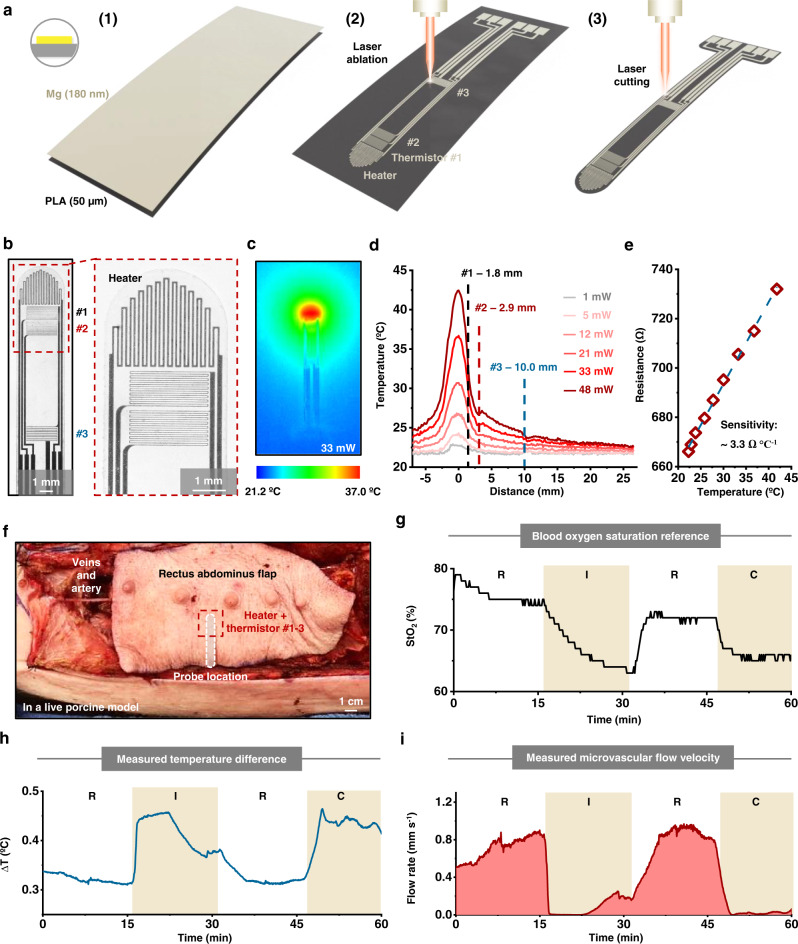
Process for fabricating bioresorbable microvascular flow sensing probes. Procedures. **a** Schematic illustration of the process, consisting of (1) vacuum deposition of a uniform Mg layer (thickness: 180 nm) on a PLA substrate (thickness: 50 µm), (2) ablation of the Mg layer to define four resistive-type devices, (3) and ablation of the PLA substrate to define a narrow, needle-shaped geometry. **b** Optical micrograph of such a device, with a magnified image in the inset to highlight the structures. **c** Temperature distribution around the probe. An input power of 33 mW generates a maximum temperature increase of ~16 °C in air. **d** Temperature distribution along with the flow probe at different heater powers. **e** Relationship between the resistance of the thermistor and temperature is linear over this range, with a sensitivity of ~3.3 Ω °C^−1^. In vivo acute evaluations in a porcine model using a rectus abdominus myocutaneous flap. **f** Optical image of the left rectus abdominus flap of a porcine model. Occlusion of the artery and veins leads to ischemia and congestion, respectively. The gray pattern indicates the location of the bioresorbable microvascular flow sensing probe. **g** StO_2_ status of the flap measured by a commercial reference device for different situations (R: release, no occlusion; I: ischemia, artery occlusion; C: congestion, vein occlusion). **h** Temperature difference between two thermistors measured by the bioresorbable device in these situations during operation of the heater. The temperature differences are ~0.35 °C in the R state, ~0.45 °C in the I and C states, respectively. **i** Corresponding microcapillary flow rate of the flap determined from these data for each situation. The microcapillary flow rates are 0.8 ± 0.2 mm s^−1^ in the R state, 0.2 ± 0.1 mm s^−1^ in the I state, and 0.05 ± 0.02 mm s^−1^ in the C state, respectively.

Early detection of thrombosis (blood clotting) in transplants is essential to preserve blood circulation and eliminate serious complications^54,55^. A class of devices with potential relevance in this context takes the form of a flexible, bioresorbable needle-shaped probe with capabilities in monitoring microvascular flow in soft tissues by using techniques in thermodilution. The laser ablation process can produce such types of probes. The device includes a thin-film heater (material: Mg; thickness: 180 nm; trace width: 50 µm; spacing: 75 µm; resistance: ~350 Ω) and three thin-film thermistors (material: Mg; thickness: 180 nm; trace width: 18 µm; spacing: 30 µm; resistance: ~650 Ω) each positioned at a different distance to the heater (1.8, 2.9, and 10.0 mm, respectively) and all integrated onto a needle-shaped polymeric substrate (material: PLA; thickness: 50 µm; dimensions: 30 × 3.5 mm). Figure 4a illustrates the fabrication procedures. Vacuum depositing a uniform layer of Mg (thickness: 180 nm) on a PLA substrate (thickness: 50 µm) prepares the structure for processing, as shown in step (1). Ablation patterns the Mg layer into narrow traces that form four thin-film resistive-type devices along the length of the substrate, as shown in step (2). Another ablation process cuts the PLA substrate into a needle-shaped structure, shown in step (3). Replacing the Mg layer with a multi-layered stack of SiO_2_/Mg/SiO_2_ (thickness: 100/180/100 nm) can enhance the performance stability when surrounded by biofluids. Laminating a layer of PLGA (thickness: ~10 µm) on top and then sandwiching the device with layers of BTP as top and bottom encapsulants complete the fabrication. Supplementary Fig. 21 presents bioresorption of a device without encapsulation in a solution that approximates the chemistry of biofluids (PBS at 95 °C, pH 7.4). The results show that the materials largely dissolve within 5 days and that elimination of residues occurs after 10 days.

Figure 4b presents an optical micrograph of the probe. An inset shows a magnified view of the structures of the heater and thermistors. Results of characterizing the heater and thermistors appear in Fig. 4c–e. The heater produces a temperature distribution around the probe, with a maximum temperature increment of ~16 °C in air for an input power of 33 mW, shown in Fig. 4c. As the heater power rises from 1 to 48 mW, the maximum temperature increases and the heat spreading region expands, shown in Fig. 4d. The temperature values at the locations of the three thermistors are different due to the different distances to the heater. The thermistors exhibit a proportional relationship between the resistance and temperature, shown in Fig. 4e, with a sensitivity of ~ 3.3 Ω °C^−1^.

In vivo acute evaluations in a porcine model demonstrate capabilities in accurate, continuous monitoring of microcapillary flow velocity using this device. The model consists of the rectus abdominus myocutaneous flap (dimensions: ~30 × 10 × 2 mm) connected to the surrounding tissues by one main artery and three main veins, as shown in Fig. 4f. Clamping the corresponding vein and artery simulates venous (“congested states”, C) and arterial (“ischemia states”, I) thrombosis states, respectively, both of which block capillary blood flow and jeopardize flap viability. R stands for “release state” without clamping the vein or artery. As shown in Fig. 4g, in both I and C states, the lack of microcapillary blood flow leads to a drop in blood oxygen saturation (StO_2_), measured by a commercial oximeter reference mounted on the flap paddle. Inserting the bioresorbable probe into the flap allows measurements of the capillary blood flow rate, shown in Fig. 4h, i. The heater generates thermal power inside the flap, and measurements from the thermistors capture the resulting changes in temperature. The difference between the temperature of adjacent thermistors (∆T_12_) appears in Fig. 4h. In both I and C states, the temperature differences are large (~ 0.45 °C) compared with that in the R state (~ 0.35 °C). Converting the temperature difference into microcapillary blood flow velocities (Supplementary Note 3) yields values of 0.8 ± 0.2 mm s^−1^ in the R state, 0.18 ± 0.13 mm s^−1^ in the I state, and 0.05 ± 0.02 mm s^−1^ in the C state, shown in Fig. 4i. Notably, the immediate change in the measured flow rate upon entering the I or the C state illustrates capabilities for fast diagnosis and early detection of adverse conditions related to restrictions in blood circulation. Images from hematoxylin and eosin (H&E) staining of tissue adjacent to the location of the probe collected from a rat model at 4 and 8 weeks after implantation appear in Supplementary Fig. 22. The results indicate the biocompatibility of the devices and the products of their dissolution in biofluids.

### Processing of multi-layered structures for bioresorbable Si-based devices

**Fig. 5:**
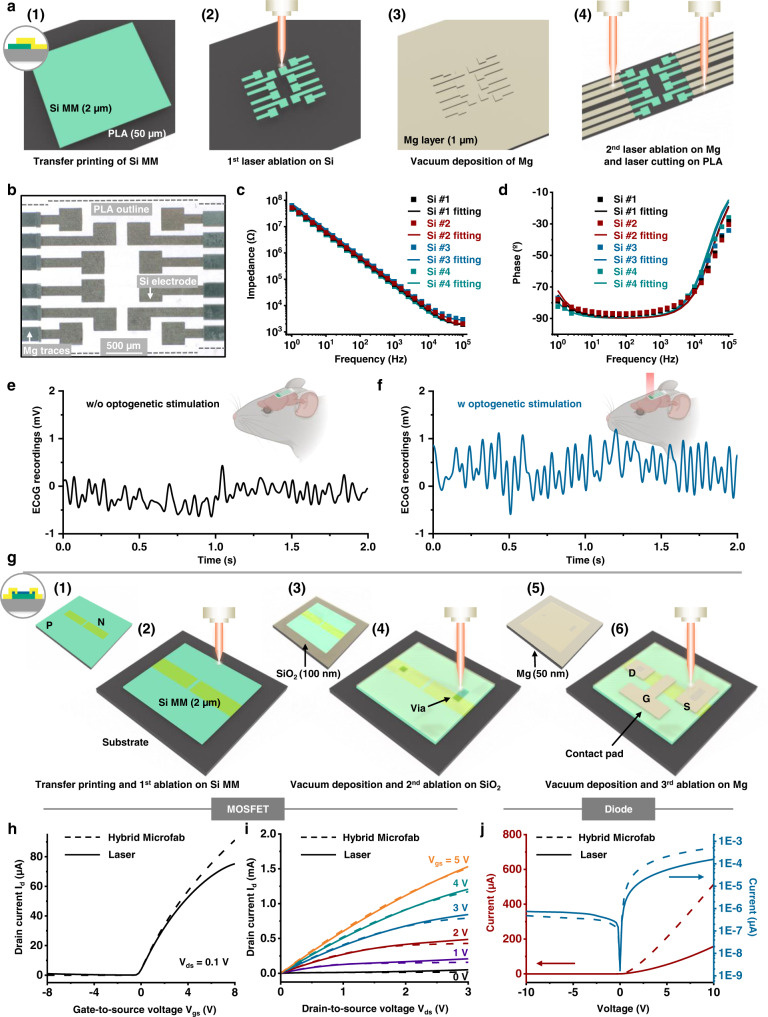
Multi-layer fabrication strategies for Si-based devices. Si-based electrode arrays. **a** Schematic illustration of the process to form the multi-layer device, consisting of (1) transfer printing of a monocrystalline highly n-doped Si MM (thickness: 2 µm) on a PLA substrate (thickness: 50 µm), (2) ablation to pattern the Si MM into an array of electrodes, (3) vacuum deposition of a uniform Mg layer (thickness: 1 µm), and (4) ablation of the Mg layer to define connection traces and to cut the PLA substrate into a ribbon shape. **b** Optical micrograph of such a device, with 12 Si electrodes and Mg traces. Electrochemical impedance spectrum (**c** impedance; **d** phase angle) of three representative electrodes measured in PBS (pH 7.4) at room temperature (dash line). The interface charge transfer resistance, *R*_*ct*_, and double layer capacitance, *C*_*dl*_, are ~250 MΩ and 2.8 µF cm^−2^, respectively, from fits to an equivalent Randles circuit (solid line). In vivo acute optogenetic evaluations in a mouse model. In vivo ECoG recordings in a mouse measured by a representative Si electrode without (**e**) and with (**f**) optical simulation. Si-based MOSFETs and diodes. **g** Schematic illustration of the process to form a Si-based n-channel MOSFET, consisting of (1) doping of specific regions of a Si MM (thickness: 2 µm), (2) ablation to pattern the Si MM into a rectangular pattern, (3) vacuum deposition of a uniform SiO_2_ dielectric layer (thickness: 100 nm), (4) ablation of the SiO_2_ layer to define shapes similar to those of Si and with two openings for metal interconnection, (5) vacuum deposition of a uniform Mg layer (thickness: 50 nm), and (6) ablation of the Mg layer to define three connection pads as electrodes. Transfer characteristics (**h**) and output characteristics (**i**) of bioresorbable n-channel MOSFETs formed by ablation (solid line) and previously reported approaches (dash line) with the same materials and dimensions. **j** IV characteristics of bioresorbable Si diodes with the same materials and the same dimensions fabricated by ablation (solid line) and previously reported approaches (dash line) in linear and log scale.

Multi-layered silicon-based electronic architectures can also be realized through multiple cycles of material preparation-patterning. Example devices exploit device-grade, monocrystalline doped Si MMs as the semiconductor for active electronic systems that also include metal interconnection traces and patterned dielectric films. (Examples of resistive-type and capacitive-type Si devices appear in Supplementary Figs. 23, 24). A flexible Si-based bioresorbable multiplexed electrode array using Si MMs as sensing components and films of Mg as interconnection traces serves as a demonstration platform designed for neurophysiologic monitoring of brain function. Figure 5a illustrates the fabrication procedures. Transfer printing a highly n-doped Si MM (thickness: 2 µm) onto a PLA substrate (thickness: 50 µm) defines the initial material structure, as illustrated in step (1). Ablation patterns the Si MM into electrode shapes (number: 12; dimensions of the electrode pad: 300 × 300 µm; sensing area in total: 2 × 2 mm) with interconnection sites (trace width: 120 µm), as shown in step (2). Another cycle of ablation defines three alignment markers (diameter: 1.5 mm) on the corners of the Si pattern. The second cycle of material preparation-patterning begins with vacuum deposition of a uniform layer of Mg (thickness: 1 µm) on top. Ablation then patterns the Mg into interconnection traces registered to the alignment markers, with minimal damage to the underlying Si layer, as shown in step (3). Another cycle of ablation cuts the PLA substrate into the shape of a ribbon, as shown in step (4). Laminating a layer of PLGA patterned by ablation on top (thickness: ~10 µm) encapsulates the system but leaves the Si sensing areas exposed (1.8 × 1.8 mm), to complete the fabrication. An optical micrograph of such a device appears in Fig. 5b. An alternative laser fabrication process appears in Supplementary Note 4 and Supplementary Fig. 25. This strategy performs ablation after material preparation, as a means to eliminate errors from overlay registration.

Characterization by electrochemical impedance spectroscopy (EIS) quantifies the performance of these Si electrodes in phosphate-buffered saline (PBS; pH: 7.4) at room temperature. Figure 5c, d corresponds to the impedance (|Z|) and phase (°) values. At frequencies relevant to neural sensing (~1 kHz), the impedances are ~50–80 kΩ and the phase angle suggests capacitive behaviors (~−90°), consistent with related types of Si devices fabricated using conventional schemes^11,56^. An equivalent Randles circuit, as shown in Supplementary Fig. 26, indicates that the interface charge transfer resistance, *R*_*ct*_, and double layer capacitance per unit area, C_dl_, are ~250 MΩ and 2.8 µF cm^−2^, respectively. All of these values are in a range consistent with silicon electrodes fabricated by conventional photolithography processes^11,56,57^.

In vivo electrocorticogram (ECoG) recordings in a mouse model illustrate the performance, summarized in Fig. 5e, f. A device and a control electrode lie on the exposed surface of the right primary motor cortex (dimension: 3 × 3 mm) of an anesthetized mouse (details in methods section). A representative segment (duration: 2 s) of ECoG recordings of sleep spindle activity appears in Fig. 5e. Optogenetic activation of ChrimsonR by illumination with a light-emitting diode (LED; wavelength: 570 nm; power density: ~10 mW/mm^2^) leads to a considerable increment in ECoG signal, shown in Fig. 5f, reaching ~1.0 mV in amplitude when compared to those without optical stimulation (~0.5 mV). The Si electrode has a thickness of ~2 µm and partial transparency (~40% at 570 nm, calculated according to the equation: absorption = log (1/transmittance)^58^) to allow light penetration for co-located optogenetic activation. Similar multi-layer schemes can be used to form other classes of devices, such as Si-based temperature sensors with higher sensitivity than thermistors formed with conventional metals (Supplementary Fig. 27).

Si-based MOSFETs and diodes are essential elements of advanced eco/bioresorbable electronic systems. Previous publications describe their fabrication based on multiple cycles of photolithography, anisotropic substrate etching, passivation, and transfer printing, with an overall scheme designed specifically to avoid degradation of the bioresorbable constituent materials due to thermal and chemical exposures associated with the processing steps^59,60^. As an alternative, ablation enables highly simplified routes to similar devices, without compromising their performance parameters. Figure 5g illustrates the procedures for a bioresorbable n-channel MOSFET as a representative example, formed by three cycles of material preparation-patterning in a sequence similar to that for the ECoG systems mentioned above. The patterned materials are monocrystalline doped Si (thickness: 2 µm), SiO_2_ (thickness: 100 nm), and Mg (thickness: 50 nm). Supplementary Fig. 28 presents schematic illustrations and an optical micrograph of the resulting device. The bottom Si MM (dimension: 505 × 515 µm) includes two highly n-doped areas (dimension: 100 × 248.5 µm), with the other areas lightly p-doped (channel dimension: 18 × 100 µm). The middle SiO_2_ film covers the underlying Si MM, with two openings (dimensions: 40 × 40 µm and 40 × 100 µm, respectively) to allow for connection between the Si and Mg pads for electrodes on top. The dimensions for the source, drain, and gate pads are 125 × 205 µm, 100 × 135 µm, and 65 × 180 µm, respectively. Figure 5h–i shows transfer and output characteristics of devices formed by laser ablation (solid line) and by previously reported schemes (dashed line), with the same materials and dimensions. The effective mobility (*µ*_*eff*_), on-off current ratio (*I*_*on/off*_), threshold voltage (*V*_*th*_), and subthreshold slope (SS) values for MOSFETs by laser ablation are 610 ± 20 cm^2^ S^−1^ V^−1^, 700 ± 120, −0.35 ± 0.05 V, and 330 ± 60 mV decade^−1^, respectively; those for devices fabricated by microfabrication are 620 ± 30 cm^2^ S^−1^ V^−1^, 3100 ± 2600, −0.30 ± 0.05 V, and 280 ± 50 mV decade^−1^, respectively.

Similar procedures can produce diodes, as illustrated in Supplementary Fig. 29a–e. Figure 5j compares the characteristics of devices formed by laser ablation (solid line) and previously reported schemes (dashed line), again with the same materials and dimensions. The I_on/off_ and ideality factor (*n*) for diodes fabricated by laser ablation are 200 ± 60 and 1.6 ± 0.3, respectively; those fabricated by hybrid microfabrication show values of 990 ± 160 and 1.4 ± 0.2, respectively. The differences are likely due to small residual amounts of Mg. As expected, these devices exhibit a photoresponse comparable to that of devices formed by hybrid microfabrication, as in Supplementary Fig. 29f, g. As shown in Supplementary Fig. 11b, some debris with characteristic sizes of ~1 µm appears on the surface of the ablated polymeric substrate, and that with sizes of ~100 nm appears on both the Si ribbon and polymeric substrate. According to the observed performance of the MOSFETs and diodes, this particulate debris has no significant effect on the operation of fabricated devices.

### Integrated processing for wireless bioresorbable systems for cardiac monitoring

**Fig. 6:**
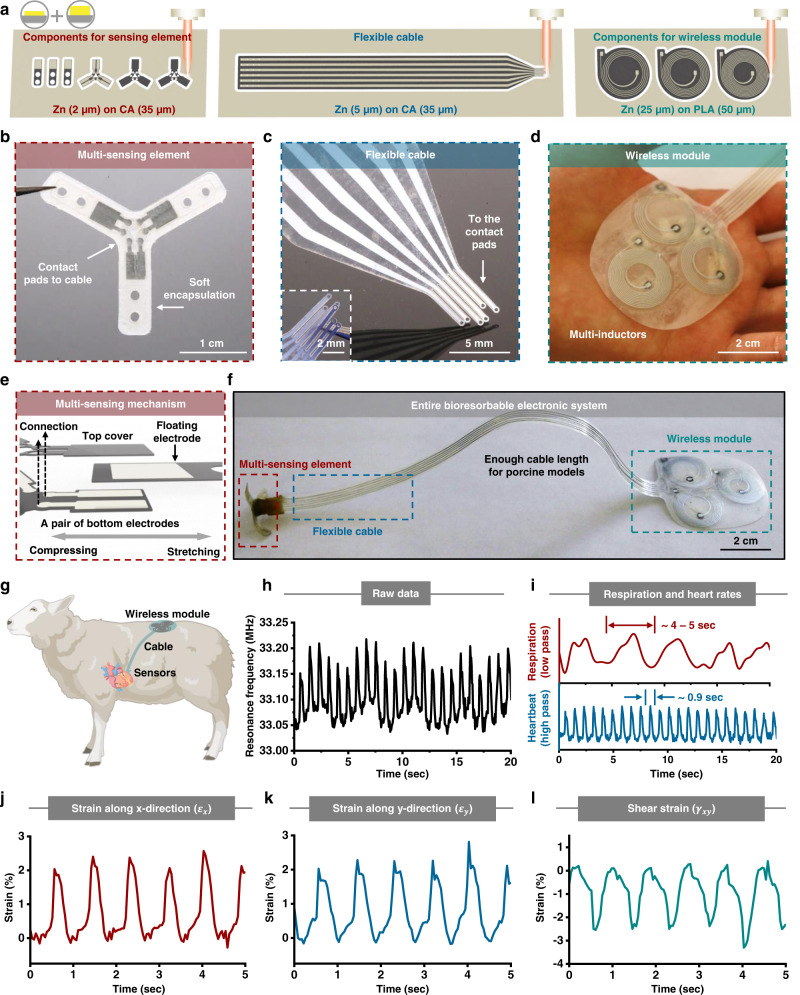
Laser ablation for integrated wireless bioresorbable cardiac systems. **a** Schematic illustration of the components used to construct the cardiac devices. **b** Photograph of the multi-sensing element. Constituent components include, from bottom to top: a bottom WPU encapsulation (thickness: ~100 µm), a tri-arm Y-shaped layer with three pairs of bottom electrodes, three single-arm floating electrodes, and a tri-arm Y-shaped top cover, and another top WPU encapsulation. The hole on each arm close to the center aids device mounting with a customized accessory. The hole close to the end facilitates suturing. **c** Photograph of the flexible cable. The element possesses six independent Zn traces (thickness: 5 µm) on a CA substrate (thickness: 35 µm), with a uniform PLGA layer as the top encapsulation (thickness: ~10 µm). **d** Photograph of the wireless module. The element adopts three Zn inductors (thickness: 25 µm) with different inductance values on a PLA substrate (thickness: 50 µm). A pair of PLGA layers serves as top and bottom encapsulation (thickness: 50 µm). **e** Working principle of the device. Strains along each arm lead to changes in the relative position between the floating electrode and the pair of bottom electrodes. This change in position leads to a corresponding change in capacitance that, in turn, changes the frequency of the resonance. **f** Photograph of the complete device for measuring strains on the surface of cardiac tissue in large animals. In vivo acute evaluations in an ovine model. **g** Schematic illustration of a device designed for measurements on the LV of an ovine animal. The multi-sensing element integrates on the LV. The wireless module lies in the dorsal subcutaneous area. A flexible cable provides an electrical interface. **h** Raw data from one sensing unit. **i** Filtering the raw data yields the respiration rate (duration: ~4–5 s; bandpass <1 Hz; marked red) and the heart rate (duration: ~0.9 s; bandpass: 40–200 Hz; marked blue). Quantitative assessment of principal strains (*ε*_*x*_, **j**; *ε*_*y*_, **k**) and shear strain (*γ*_*xy*_, **l**) of the ovine LV.

Monitoring of mechanical strains associated with cardiac activity can be valuable in the diagnosis of cardiac disorders and early detection of cardiac arrest^61^. In an example relevant to this area of medical monitoring, an integrated processing sequence yields a type of wireless, bioresorbable, mechanically compliant, multi-sensing electronic system for measuring principal and shear strains on the surface of the heart. A unique device of this type, formed by laser ablation processes, consists of a multi-sensing capacitive element, a flexible cable with six connection traces, and a wireless module with three in-plane inductors, as illustrated in Fig. 6a and shown in the photographs of Fig. 6b–d. The multi-sensing element (Fig. 6b) includes several components from bottom to top: a tri-arm Y-shaped layer with three pairs of bottom electrodes (material: Zn/CA; thickness: 2/35 µm; dimension of each arm: 6.9 × 3.1 mm), three single-arm floating electrodes (material: Zn/CA; thickness: 2/35 µm; dimension: 10.9 × 3.1 mm), and a tri-arm Y-shaped top cover (material: CA; thickness: 35 µm; dimensions of each arm: 6.9 × 3.3 mm), shown in Supplementary Fig. 30a–c. Two uniform layers of bioresorbable wax-based polyurethane (WPU) elastomers (Young’s modulus: ~100 kPa; Supplementary Fig. 31a) act as top and bottom encapsulation layers (thickness: ~100 µm; dimensions of each arm: 15.0 × 5.0 mm). The WPU elastomers, based on dynamic covalent polyurethanes^62^, support improved water barrier properties through the addition of natural wax, without compromise in mechanical properties, shown in Supplementary Fig. 31b–d. Stable recording is possible for several hours in vivo. Enhancing the water barrier properties of the encapsulation material is critical to increasing the functional lifetime of the device. The hole closest to the center of the device on each arm facilitates device manipulation with a custom 3D-printed attachment (Supplementary Fig. 32). The hole close to the end allows for suturing to underlying cardiac tissues. The flexible cable (Fig. 6c) includes six traces of Zn (thickness: 5 µm; trace width: 1 mm; spacing: 1.5 mm) on a cellulose acetate (CA) substrate (thickness: 35 µm; width: 14 mm). A uniform layer of PLGA serves as the top encapsulation (thickness: ~10 µm). The wireless module (Fig. 6d) consists of three Zn inductors (thickness: 25 µm; line width: 200 µm; spacing: 500 µm; outside diameter: 19.6 mm; number of turns: 4, 6, and 10) on a PLA substrate (thickness: 50 µm) with different inductance values to achieve different resonance frequencies for each of the three capacitive sensors in the multi-sensing element. Two uniform PLGA layers act as the top and bottom encapsulation structures (thickness: 50 µm). Stretching each arm of the multi-sensing element along its axis leads to a change in the relative position between the floating electrode and the pair of bottom electrodes. The result is a corresponding change in the capacitance between the pair of the bottom electrodes, as shown in Fig. 6e and Supplementary Fig. 30d, e. These changes alter the resonance frequencies of the LC oscillators along each arm (Supplementary Note 5 and Supplementary Fig. 33–35) which can be converted to the principal strains (*ε*_*x*_ and *ε*_*y*_) and shear strain (*γ*_*xy*_) by coordinate transformation (Supplementary Fig. 36). A photograph of the entire system appears in Fig. 6f.

Characterization of the device reveals high strain sensitivity, negligible bending sensitivity, and minimal mechanical load on the underlying tissue. The variance of the resonance frequency in each arm is ~ 3–4 % at a maximum strain of ~18 %, indicating a sensitivity of ~200 kHz per unit strain (Supplementary Fig. 37). The tri-arm Y-shaped top cover fixes the distance between the floating electrode and the pair of bottom electrodes. In this way, the capacitance remains largely unchanged by bending deformations. Even at a bending angle of 90°, the change in the resonance frequency of each arm is smaller than 0.2%, which is negligible compared to changes associated with stretching through a cardiac cycle (Supplementary Fig. 37c) and consistent with simulation results (Supplementary Fig. 38).

Characterization by 3D-particle tracking velocimetry (3D-PTV)^63,64^ proves that the device is mechanically compliant with the cardiac tissues, with negligible constraint. The strain distributions on the same position of the porcine cardiac surface without and with a mounted device during the cycles of expansion and contraction (duration: 4.5 s for each stage) appear in Supplementary Fig. 39. The deformations with the device (strain at the full volume: ~8%) are similar to those without the device (strain at the full volume: ~9%). By contrast, the strain at full expansion decreases ~2% as a result of mounting a uniform film in a similar shape (Young’s modulus: ~1 GPa) on the cardiac surface. The mechanical compliance of the device corresponds well with simulation results (Supplementary Fig. 40) and allows accurate recordings of natural cardiac deformations.

Ex vivo and in vivo evaluations highlight the functionality. The ex vivo studies utilize an artificial heart system with adjustable and oscillatory flow and pressure. Mounting the device on the right ventricle (RV) of a porcine heart with three suture sites on the ends of each arm allows the compliant deformation of the device for mounting on the heart. Supplementary Fig. 41a, b presents the resonance frequency for the three sensing units, each of which follows the reference pressure and flow rate data in terms of frequency and qualitative waveform shapes. The maximum change in frequency increases as the pressure in the pulmonary artery (PA) rises (Supplementary Fig. 41c). Converting the frequencies to strains along each arm, and then analyzing the strains according to the coordinate transformation determines the principal and shear strains of the local position, as shown in Supplementary Fig. 42.

In vivo evaluations utilize the left ventricle (LV) of the heart in an ovine model. The multi-sensing element mounts on the LV, and the wireless module lies in the dorsal subcutaneous area, with the electrical connections supported by a flexible cable, shown in Fig. 6g. Figure 6h presents the raw data from one sensing unit in a representative segment (duration: 20 s; raw data for the three units appear in Supplementary Fig. 43). Filtering the data in different cutoff frequencies (Supplementary Fig. 44) yields the respiration rate (duration: ~ 4–5 s; marked red) and heart rate (duration: ~0.9 s; marked blue) of the anesthetized ovine animal, shown in Fig. 6i. Analyzing the data from the three sensing units together through coordinate transformation results in principal strains (*ε*_*x*_ and *ε*_*y*_) and shear strain (*γ*_*xy*_) (Supplementary Note 6, Supplementary Fig. 45 and Fig. 6j–l).

The results described here span topics in laser-material interactions, fabrication sequences, material layouts, device architectures, and system-level demonstrations of eco/bioresorbable electronics through ex vivo and in vivo studies, all in the context of a scanned, picosecond-pulsed laser ablation approach to manufacturing. Systematic characterization illustrates compatibility with the full range of eco/bioresorbable materials, with the ability for precise thickness control, high spatial resolution, and good overlay registration in multi-layered configurations. Successful fabrication of silicon MOSFETs and diodes suggests applicability to nearly all classes of IC and CMOS components, in isolation or as interconnected collections. Comprehensive evaluations in animal models highlight good performance of a diverse collection of bioresorbable electronic devices across multiple organs, ranging from wireless physiological monitors in tissue cavities to microcapillary flow sensing probes in transplanted tissues, biopotential sensing arrays for the cerebral cortex, and wireless strain systems for the epicardial surface. Because this dry manufacturing process aligns well with established tools adapted from the flexible printed circuit board industry, it is possible to envision use at industrial scales. These concepts may form a starting point for further developments of laser-based schemes for miniaturized, high-density, and stacked IC and microelectromechanical systems (MEMS) chips in eco/bioresorbable forms and high-resolution features, particularly when used in conjunction with previously reported schemes for transfer printing of eco/bioresorbable components produced using foundry facilities^60,65^. Additional improvements may include increasing the resolution and overlay registration through advanced optics, e.g., flat-topped laser spots, and mechanical stages, introducing fast switching of laser wavelengths selected to match the absorption spectra of processed materials, incorporating in-line sensing for closed-loop control of manufacturing processes, and extending applications to include multi-layer patterning directly on biomaterials or living tissues.

## Methods

### Materials

#### Materials

Materials used in this study included bioresorbable substrates, conductors, semiconductors, dielectrics, and encapsulants. Polymer substrates consisted of cellulose acetate (CA; thickness: 35 µm; Goodfellow Corporation, PA, USA), poly (lactic acid) (PLA; thickness: 50 µm; Goodfellow Corporation, PA, USA), and poly(D,L-lactide-*co*-glycolide) (PLGA; lactide:glycolide: 65:35; *M*_*w*_: 40–75k; thickness: ~10 µm; Sigma-Aldrich, MO, USA). Electron beam evaporation (AJA International Inc., MA, USA) yielded uniform layers of metal (materials: Mg, Zn, and Fe) on these substrates. A bioresorbable thermoplastic polymer (BTP; synthesis process appears in the following section) enabled robust lamination of uniform metal films (thickness: 5–250 µm; materials: Mg, Zn, Mo, and Fe; Goodfellow Corporation, PA, USA) on these substrates. Device-grade, monocrystalline silicon membranes (thickness: 0.5–2 µm) were obtained from silicon on insulator wafers (SOI wafers; University Wafer, Inc., MA, USA) by eliminating the buried oxide layer via immersion in hydrofluoric acid (HF; concentration: 49%; Honeywell, NC, USA) for 2 d. These membranes were doped by ion implantation (Leonard Kroko, Inc., CA, USA) or diffusion (Ty star Corporation, CA, USA) in patterns and at levels to match device requirements. Annealing by a rapid thermal processor (RTP; AW-610; Allwin 21 Corporation, CA, USA) preceded immersion in HF to eliminate the buried oxide. Uniform layers of dielectric materials, such as SiO_2_, (thickness: 10–100 nm), were deposited by electron beam evaporation (AJA International Inc., MA, USA).

#### Synthesis

Chemical synthesis procedures yielded BTP and WPU polymers. The synthesis of BTP followed a previously reported strategy based on a thiol-yne step-growth polymerization^66^. Briefly, two monomers, bis(3-mercaptopropyl) succinate (C_SS_; Sigma-Aldrich, MO, USA) and 1,3-propane diyl dipropiolate (C_3A_; Sigma-Aldrich, MO, USA), were synthesized by sulfuric acid-catalyzed Fischer esterification reactions in toluene. C_SS_ was synthesized from 3-mercaptopropanol (25.000 g, 271 mmol, 2.14 equiv; Sigma-Aldrich, MO, USA) and succinic acid (15.000 g, 144 mmol, 1 equiv; Sigma-Aldrich, MO, USA). C_3A_ was synthesized from propanediol (20.000 g, 263 mmol, 1 equiv; Sigma-Aldrich, MO, USA) and propiolic acid (50.000 g, 714 mmol, 2.7 equiv; Sigma-Aldrich, MO, USA). All three monomers, C_SS_ (0.715 g, 0.2 equiv), C_3A_ (2.420 g, 1 equiv), and 1,10-decanedithiol (C_10S_; 2.210 g, 0.8 equiv; TCI America, Inc., OR, USA) were distilled prior to addition to an oven-dried round bottom flask charged with HPLC-grade chloroform (CHCl_3_; 25 mL; Thermo-Fisher Scientific, MA, USA). The resulting solution was cooled to −15 °C while stirring for 15 min before adding 1,8-diazabicyclo(5.4.0)undec-7-ene (DBU; 20 μL; Sigma-Aldrich, MO, USA) and then warming to room temperature 5 min after addition. The reaction proceeded for 2 hr then a slight excess of C_3A_ (1–2 drops) was introduced to cap terminating thiol moieties. Shortly after, butylated hydroxytoluene (BHT; 0.225 g; Sigma-Aldrich, MO, USA) was added to quench DBU, then the solution was precipitated into diethyl ether (400 mL; Sigma-Aldrich, MO, USA) and filtered to give a yellow polymer which was subsequently dried under high vacuum at ambient temperature for 24 h. Size-exclusion chromatography (SEC; EcoSEC HLC-8320GPC; Tosoh Bioscience LLC, PA, USA) and differential scanning calorimetry (DSC; Q2000 DSC Packing; TA Instruments, DE, USA) determined the molecular weight (*M*_*n*_ = 32.5 kDa; *M*_*w*_ = 68.2 kDa; *Ð*_*M*_ = 2.1) and the melting temperature (*T*_*m*_ = 99.3 °C), respectively. Fabrication of uniform layers of BTP utilized compression molding (temperature at 120 °C and compression at 22 kN for 10 min, 44 kN for 10 min, and 66 kN for 60 min, respectively), with a polyimide (PI) mold (thickness = 10–200 μm) treated by anti-adhesion spray to achieve target thicknesses. The synthesis of WPU followed previous protocols^62^, modified by adding a natural wax mixture (ratio of Candelilla wax to Beeswax: 4:1) to enhance the water barrier property. In general, the synthesis began with melting polycaprolactone triol (PCL-triol; M_n_: ~ 900 g/mol; 2.7 g; Sigma-Aldrich, MO, USA) at 60 °C, followed by mixing with hexamethylene diisocyanate (HDI; 504 µL; Sigma-Aldrich, MO, USA) and butyl acetate (15 mL; Sigma-Aldrich, MO, USA) until full dissolution at the same temperature. Adding tin(II) 2-ethylhexanoate (Sn(Oct)_2_; Sigma-Aldrich, MO, USA) after cooling the solution to room temperature and then drop-casting the resulting solution on a hydrophobic glass surface yielded films with the desired thickness (~100 µm) defined by the volume and area.

### Tool and processing modes for laser ablation

The ablation processes described here used a commercial picosecond-pulsed laser system (wavelength: 1030 nm; pulse duration: 1.0 ± 0.2 ps; beam diameter: 15 µm; maximum processing area: 30 cm × 23 cm; LPKF Laser & Electronics, OR, USA). The average power (80–200 mW), scanning speed (200–600 mm/s), frequency (40–200 kHz), and the number of repetitions were adjusted according to different application scenarios. Grid distance (2–7 µm) and grid mode (X-parallel, Y-parallel, and XY-parallel) were adjusted to meet patterning requirements. The ablation process enabled by this laser allowed material thinning, complete removal, and cutting by appropriate selection of these parameters. Thinning corresponds to controlled, patterned reductions in the thickness of the top layer to a target value. Removal corresponds to complete, patterned ablation of the top layer with minimum damage to the underlying materials. Multi-layered device architectures were fabricated by multiple cycles of material preparation-ablation. Cutting corresponded to ablation through the entire material stack, including the substrate, to achieve target shapes.

### Characterization of the ablation process

#### Ablated thickness

Square regions of monocrystalline Si (5 mm × 5 mm) were ablated using various laser settings (average power, scanning speed, frequency, and the number of repetitions) at fixed grid distance (*d*; 7 µm) and grid mode (XY-parallel). Atomic force microscopy (AFM; Dimension Icon; tapping mode; Bruker, MA, USA) defined the topography of the Si surfaces to determine the ablated thicknesses.

#### Lateral resolution

Ablation (average power: 90 mW; scanning speed: 300 mm/s; frequency: 200 kHz; number of repetitions: 3 for ribbon shape, 5 for trench shape; grid distance: 2 µm; grid mold: Y-parallel) with different projected widths between two adjacent laser scans determined ribbon (projected width: 7–23 µm) and trench (projected width: 15–23 µm) shapes in monocrystalline Si or Mg. Specifically, ribbon shapes (length: 1 mm) were created by separating two parallel ablated regions with well-controlled projected widths. Trench shapes were achieved by ablating various single-line segments (length: 1 mm) with different projected widths. AFM revealed the cross-sectional profiles of the Si ribbons and trench shapes to determine the peak heights and effective widths (integrated cross-sectional area divided by the peak height). Optical profilometry (Dektak 150 Surface Profiler; Veeco, NY, USA) characterized the cross-sectional profiles of the Mg ribbons and trenches.

#### Overlay registration

The top layer (material: Au; thickness: 1 µm) on a polymer substrate (material: polytetrafluoroethylene; thickness: 350 µm) was ablated into a crisscross pattern (length and width: 8 mm; line width: 0.5 mm; average power: 100 mW; frequency: 100 kHz; scanning speed: 500 mm s^−1^; number of repetitions: 1; grid distance: 7 µm; grid mold: XY-parallel), with three alignment marks (circle shape; diameter: 1.5 mm) on the corners. After changing the position of the sample, the alignment marks were used to perform aligned ablation of a second, smaller crisscross pattern (length and width: 6 mm; line width: 0.5 mm) on the top layer with the same central point as the first. The designed distance between the two cross patterns was 0.5 mm. An optical microscope characterized the actual distance. The difference between these distances corresponds to the overlay registration along the *x*- and *y*-axes.

#### Minimal damage to underlying substrate

Characterization involved studies of damage to a PLA film (thickness: 50 µm) when ablating a top layer of Si (thickness: 500 nm) and damage to a Si membrane (thickness: 500 nm) when ablating a top layer of Mg (thickness: 300 nm). In both cases, after completely removing the top layer by ablation in a square pattern (5 mm × 5 mm), the samples were characterized using a 3D laser scanning microscope (OLS 5000; Olympus Corp., Tokyo, Japan) to obtain the thickness of the ablated surface region *T*_*ab*_. The difference between *T*_*ab*_ and the thickness of top layer (*T*_*top*_) represented a useful metric to define the selectivity of the ablation process to the top layer.

#### Line edge roughness

Characterization involved studies of Si (thickness: 2 µm; width: 100 µm) and Mg (thickness: 500 nm; width: 100 µm) ribbons patterned on PLA substrates (thickness: 50 µm). Top-view imaging the ribbons through a digital microscope (VHX-7000 series; Keyence, IL, USA), followed by stripes analysis in ImageJ^67,68^, determined the RMS line edge roughness.

#### Surface treatment on substrates

Ablation also enabled surface texturing of polymer substrates for bioresorbable electronics. As an example, ablation defined a square pattern array (dimension: 60 µm; space: 60 µm) to generate a micro-textured surface with a Cassie-Baxter state and enhanced surface hydrophobicity.

### FEA studies of heat transfer

Analysis focused on energy absorbed from laser radiation using the transient heat transfer module of Abaqus (Dassault Systems Simulia Corp., RI, USA). The sample consisted of a 500-nm-thick Si MM on a 50-µm-thick PLA substrate. The absorbed energy was modeled by a body heat flux *Q* in the Si at the region of the laser spot (diameter *d*_0_ = 15 μm). The heat flux was uniform along the thickness direction and followed a Gaussian distribution^51^ ($Q={Q}_{{{\max }}}{{\exp }}\left(-50{r}^{2}{d}_{0}^{-2}\right)$). The thermal conductivity (*k*) and thermal diffusivity (*α*) were *k*_Si_ = 148 W m^−1^ K^−1^ and *α*_Si_ = 88.0 mm^2^ s^−1^ for Si MM, respectively, and *k*_PLA_ = 0.13 W m^−1^ K^−1^ and *α*_PLA_ = 0.058 mm^2^ s^−1^ for PLA substrate, respectively.

### Laser ablation for wireless bioresorbable physiological monitors

#### Inductors

Bonding a uniform foil of Mg (thickness: 280 µm) on a PLA substrate (thickness: 50 µm) using the BTP interfacial adhesive (5 wt% in chloroform; heat compression for 30 min) defined samples for processing. Ablation (average power: 4 W; frequency: 40 kHz; scanning speed: 400 mm s^−1^; number of repetitions: 4; grid distance: 7 µm; grid mold: XY-parallel) created a thinned central circular area of the Mg layer (thickness: from 280 µm to 190 µm) to define the bottom electrode of the device. Another ablation process (average power: 4 W; frequency: 40 kHz; scanning speed: 400 mm s^−1^; number of repetitions: 125; grid distance: 7 µm; grid mold: XY-parallel) applied to the surrounding area of the Mg created the necessary helical coil pattern (line width: 150 µm; line space: 500 µm; out diameter: 10 mm; number of turns: 6; thickness of 280 µm). Measurements using a 3D laser scanning microscope (OLS 5000) characterized the surface topography of the device.

#### Other components

Ablation defined the geometries of all components, including a circular top electrode (PLGA/Zn/PLGA; thickness: 10/2/10 µm), an annular spacer (PLA; thickness: 50 µm), and BTP encapsulation (top, thickness: 25 µm; bottom, thickness: 200 µm) by cutting. Manual assembly of the system began with adhering the circular top electrode (PLGA/Zn/PLGA) onto the top BTP encapsulation (thickness: 25 µm) using hexane to soften the surface of the BTP. Adhering the annular spacer (PLA) and the inductor with the thinned bottom electrode (Mg/PLA) to the device in sequence at 70 °C using a conductive wax (a mixture of W microparticles and Candelilla wax at a weight ratio of 16:1) connected the top and bottom electrodes. Sealing the device with a bottom encapsulating layer of BTP (thickness: 200 µm) at 120 °C completed the assembly process.

### Evaluations of wireless bioresorbable physiological monitors in a rat model

The animal studies were performed according to protocols approved by the institutional animal care and use committees at Washington University in St Louis and conformed to the Guide for the Care and Use of Laboratory Animals. Male Lewis rats weighing 250–350 g (Charles River Laboratories, MA, USA) received hair trimming, povidone-iodine spreading (10 vol%), and isopropanol spreading (70 vol%) in the surgical area, followed by subcutaneous injections of buprenorphine hydrochloride (0.05 mg/kg; Reckitt Benckiser Healthcare) and ampicillin (50 mg/kg; Sage Pharmaceuticals) for pain management and infection prevention at the implantation site, respectively. The surgeons provided appropriate postoperative care along with analgesia post-surgery. Device implantation involved a surgical process of anesthetizing the rats with isoflurane gas, holding the head in a stereotaxic frame, opening a craniectomy and dura, placing the bioresorbable device on the cortical surface, and sealing the craniectomy with a commercial dental cement on the edge of the device (Micron Superior Type 2; Prevest Denpro Limited, Jammu, India). Embedding a clinical ICP monitor (Camino System; Model MPM-1; Integra LifeSciences) in a nearby craniectomy enabled comparison testing as a reference. Carefully squeezing and releasing the flank of rats induced increments and recoveries in ICP values, respectively. The readout system included a single-turn readout coil that was connected to a vector network analyzer (E5063A; Keysight, CA, USA). The external readout coil was placed on top of the head without physical contact, with a fixed working distance of ~1 cm. The vector network analyzer measured the real and imaginary parts of S-matrix element S_11_ in real time. The measurements of intracranial pressure, respiration rate, and heart rate were performed under animal anesthesia.

### Laser ablation for bioresorbable microvascular flow sensing probes

Vacuum deposition of a uniform Mg layer (thickness: 180 nm) on a PLA substrate (thickness: 50 µm) completed the preparation step. Ablation (average power: 150 mW; frequency: 100 kHz; scanning speed: 400 mm s^−1^; number of repetitions: 9; grid distance: 7 µm; grid mold: XY-parallel) patterned the Mg into four serpentine shapes to define one resistive heater (trace width: 50 µm; spacing: 75 µm; resistance: ~350 Ω) and three thermistors (trace width: 18 µm; spacing: 30 µm; resistance: ~ 650 Ω), each with a different distance to the heater (1.8, 2.9, and 10.0 mm, respectively). Ablation then cut the PLA substrate into a needle-shaped structure (dimensions: 30 × 3.5 mm) to complete the fabrication (average power: 100 mW; frequency: 100 kHz; scanning speed: 400 mm s^−1^; number of repetitions: 60; grid distance: 7 µm; grid mold: XY-parallel). The resistive heater filled the area at the tip of the needle to ensure a spatially uniform pattern of heating. Manual assembly of the device began with bonding a uniform layer of PLGA adhesive (thickness: ~10 µm) on top of the probe at 70 °C (over the glass transition temperature (*T*_*g*_) of PLGA (~ 65 °C)). Encapsulating the probe on top and bottom with films of BTP (thickness: 25 µm) and sealing the edges using a soldering iron at 120 °C for 2 min, completed the assembly.

### Characterization of bioresorbable microvascular flow sensing probes

The characterization included studies of the thermal distributions produced by the resistive heater and of the sensitivity of the thermistors. A programmable low noise power source (6221; Keithley Instruments, OH, USA) supplied current to the heater with a power of 1–48 mW. An infrared (IR) camera (a6255sc; FLIR Systems, OR, USA) imaged the temperature distribution across the device, including the temperature values at the positions of the three thermistors. Simultaneously, a data acquisition/switch unit (34970A/34901A; Agilent Technologies, CA, USA) determined the resistances of these thermistors. The slope of the plot of resistance as a function of temperature determined the sensitivity of these thermistors, and enabled conversion from resistance to temperature.

### Evaluations of bioresorbable microvascular flow sensing probes in a porcine model

The animal studies were performed according to protocols approved by the institutional animal care and use committees at Washington University in St Louis and conformed to the Guide for the Care and Use of Laboratory Animals. Four live pigs were utilized in separate experiments for this study. Anesthesia was induced with Telazol, ketamine, and xylazine followed by maintenance with inhaled isoflurane. Pedicled rectus abdominus myocutaneous flaps were raised with the superficial superior epigastric vein, and the deep superior epigastric artery and veins were separated for occlusion. An incision along muscle fibers near the bottom surface of the rectus abdominus muscle was made by a 15# blade, followed by the probe deployment into the muscle pocket. After restoring the flap to its anatomic orientation, a commercial ViOptix device which was mounted onto the skin padder, with wire connection to an external monitor, recorded the oxygen saturation (StO_2_) of the flap as the reference. A voltage source (arbitrary waveform generator; 3390; Keithley Instruments, OH, USA) powered the heater and the data acquisition/switch unit (34970A/34901A) measured the resistance values of the three thermistors. A complete process of in vivo evaluation included ischemia (I; 15 min), recovery (R; 15 min), congestion (C; 15 min), and recovery (*R;* 15 min). An Acland clamp was applied to the right deep superior epigastric artery, which led to a complete ischemia state (I). The Acland clamps were applied to the deep and superficial superior epigastric veins to induce a venous congestion state (C). Releasing the Acland clamps from artery or veins allowed for flap recovery (R) and re-establishment of a stable baseline reading. After the completion of the experiments, the animals were euthanized with pentobarbital.

### Laser ablation for bioresorbable multi-layered Si-based electrode arrays

The process consisted of two cycles of material preparation-ablation and one cycle of cutting. Ablation (1st ablation; average power: 90 mW; frequency: 200 kHz; scanning speed: 300 mm s^−1^; number of repetitions: 25; grid distance: 6 µm; grid mold: X-parallel) patterned the doped Si MM (thickness: 2 µm) on the PLA substrate (thickness: 50 µm) to define the sensing element. Three alignment makers (diameters: 1.5 mm) were fabricated on the corners of the Si pattern. Ablation patterned a Mg layer (thickness: 1 µm) deposited by electron beam evaporation (AJA International Inc., MA, USA), into the shape of connection traces (2nd ablation; average power: 30 mW; frequency: 200 kHz; scanning speed: 400 mm s^−1^; number of repetitions: 45; grid distance: 6 µm; grid mold: X-parallel). Ablation cut the PLA substrate into a ribbon shape to complete the fabrication (average power: 1 W; frequency: 100 kHz; scanning speed: 400 mm s^−1^; number of repetitions: 80; grid distance: 7 µm; grid mold: XY-parallel). Characterization of the electrochemical impedance spectrum (impedance and phase angle) of these electrodes used an Autolab electrochemical workstation (Metrohm AG, Herisau, Switzerland) in 0.1 M PBS (pH 7.4) at room temperature.

### Evaluations of bioresorbable multi-layered Si-based electrode arrays in a mouse model

The animal studies were performed according to protocols approved by the institutional animal care and use committees at Northwestern University and conformed to the Guide for the Care and Use of Laboratory Animals. Young adult male C57BL/6 mice (postnatal day: 60–80; weight: ~20 g) were used. Mice were maintained at ~25 °C and humidity ranging from 30 to 70%, on a standard 12 h light–12 h dark cycle (lights on at 6:00 am) with ad libitum feeding. Right primary motor cortex (M2) was targeted for stereotactic injection under isoflurane anesthesia using the following coordinates relative to bregma: AP = +0.8 mm, ML = +1.5 mm and DV = −1.0 mm. AAV9-syn-ChrimsonR-tdT virus (Addgene #59171) was diluted to a titer of ~5 × 10^12^ GC mL^−1^ in PBS (pH 7.4) and injected using a glass pipette and a micro-injector. Three weeks after virus transduction, the local skull was removed to create a 3 × 3 mm^2^ cranial window for acute ECoG measurement using the multi-layered Si-based electrode array. The electrode was placed over the right primary motor cortex and a ground/reference electrode was placed at AP = +3.0 mm, ML = +0.5 mm. For optogenetic activation of ChrimsonR, an external LED driver coupled with a commutator (Plexon, Dallas, TX) provided light stimulation (wavelength: 570 nm; power density: ~10 mW mm^−2^). The LED emission terminal was placed ~1 mm over the device. The device was connected to an electrophysiology data acquisition system (RHD recording system; Intan Technologies, CA, USA) for data recording. The ECoG signal was analyzed using Open Ephys Acquisition Board and Open Ephys GUI (Open Ephys, Cambridge, MA) at 1 kHz and subsequently filtered with a bandpass filter between 0.1 and 20 Hz.

### Laser ablation for bioresorbable multi-layered Si-based semiconductor devices

The process consisted of three cycles of material preparation-ablation. Ablation (1st ablation; average power: 80 mW; frequency: 200 kHz; scanning speed: 1000 mm s^−1^; number of repetitions: 45; grid distance: 7 µm; grid mold: Y-parallel) first directly patterned a doped Si (thickness: 2 µm) to define the sensing element (the doped areas differ in MOSFETs and diodes, Supplementary Figs. 28 and 29). Patterns on the corners of the Si formed three alignment markers (diameters: 1.5 mm). Thermal oxidation (Tytan mini furnace system; Tystar Corporation, CA, USA) and plasma-enhanced chemical vapor deposition (PECVD; STS LpX CVD; SPTS Technologies, Newport, UK) formed uniform layers of SiO_2_ (thickness: 100 nm) and SiN_x_ (thickness: 200 nm) for MOSFETs and diodes, respectively, subsequently patterned by ablation (MOSFETs: average power: 70 mW; frequency: 200 kHz; scanning speed: 1000 mm s^−1^; number of repetitions: 20; grid distance: 7 µm; grid mold: Y-parallel; diodes: average power: 70 mW; frequency: 200 kHz; scanning speed: 1000 mm s^−1^; number of repetitions: 10; grid distance: 7 µm; grid mold: Y-parallel) to form several vias for connection. Ablation patterned a layer of Mg deposited by electron beam evaporation (thickness: 50 nm for MOSFETs and 100 nm for diodes) into the shape of connection pads (dimension: 160 × 580 µm; average power: 30 mW; frequency: 200 kHz; scanning speed: 1000 mm s^−1^; number of repetitions: 6 for MOSFETs and 29 for diodes; grid distance: 2 µm for MOSFETs and 7 µm for diodes; grid mold: Y-parallel for MOSFETs and XY-parallel for diodes). Characterization utilized a manual probe station (S-1160; Signatone, CA, USA) with a semiconductor parameter analyzer (4155C; Keysight, CA, USA). A white LED (10,000 lux) for basic measurements of the photoresponses of the diodes.

### Laser ablation for wireless bioresorbable cardiac systems

#### Multi-sensing element

This element included several components, from bottom to top: a uniform bottom encapsulating layer of WPU (thickness: ~100 µm; dimensions of each arm: 15.0 × 5.0 mm), a tri-arm Y-shaped layer with three pairs of bottom electrodes (Zn/CA; thickness: 2/35 µm; dimensions of each arm: 6.9 × 3.1 mm), three single-arm floating electrodes (Zn/CA; thickness: 2/35 µm; dimensions: 10.9 × 3.1 mm), a tri-arm Y-shaped top cover (CA; thickness: 35 µm; dimensions of each arm: 6.9 × 3.1 mm), and a top encapsulating layer WPU (thickness: ~100 µm; dimensions of each arm: 15.0 × 5.0 mm). The ablation process patterned all of these components. For the bottom electrodes, the floating electrodes, and the top cover, ablation directly patterned the top Zn layer (thickness: 2 µm; vacuum deposition through electron beam evaporation) on the CA substrate (thickness: 35 µm). Ablation cut the WPU (tri-arm; dimensions of each arm: 15.0 × 5.0 mm) into desired shapes. After stacking these components, bonding the edge of the top and bottom WPU encapsulation layers by heat compression at 200 °C (bonding width at edge: ~0.9 mm) completed the fabrication.

#### Flexible cable

The flexible cable included an array with six independent traces (material: Zn/CA; thickness: 5/35 µm) and a uniform layer of PLGA as encapsulation (thickness: ~10 µm). For the traces, the ablation process directly patterned the top Zn layer (number: 6; trace width: 1 mm; trace space: 1.5 mm; length: 120 mm) on the CA substrate (dimensions: 120 mm × 14 mm). Laser cutting of a uniform layer of PLGA defined the top encapsulation (dimensions: 120 mm × 14 mm). Heat compression at 70 °C facilitated the bonding of the encapsulation. Long cables utilized Zn wires (diameter: 250 µm; Goodfellow Corporation, PA, USA) sandwiched by uniform layers of PLGA using heat compression at 70 °C, as an extended connection between the flexible cable and the wireless module.

#### Wireless module

The wireless module included three in-plane inductors (Zn/PLA; thickness: 25/50 µm) with different inductance values, encapsulated by two uniform layers of PLA (thickness: 50 µm). Ablation defined all of the components. For in-plane inductors, BTP served as an adhesive between the Zn film (thickness: 25 µm) and the PLA substrate (thickness: 50 µm). Ablation patterned the top Zn layer (thickness: 25 µm) into a helical coil shape (line width: 200 µm; line space: 500 µm; outside diameter: 19.6 mm; number of turns: 4, 6, and 10). Ablation cut the PLA encapsulation layer (diameter: 90 mm) and the substrate (diameter: 22.6 mm) into the desired shapes. The three inductors were each positioned between PLA encapsulation layers to achieve optimized Q factors, defined by measurements of S_11_, when evaluated together using a single-turn readout coil (diameter: ~5 mm) connected to a vector network analyzer (E5063A; Keysight, CA, USA). Tightly bonding these components together by spreading a small amount of chloroform and heat compression at 120 °C in sequence completed the fabrication.

#### Connection among the modules

Connections between the multi-sensing element and the flexible cable used six independent traces interfaced to the six pads of the bottom electrodes in the multi-sensing element using bioresorbable conductive wax (Supplementary Fig. 46a). To suppress the parasitic capacitance in the cable, the sequence of the six independent traces followed the order of bottom electrode pair #1-#2-#3-#1-#2-#3. In this way, the distance between the two traces for the same bottom electrode pair was controlled to ~6.5 mm. A bioresorbable polymer mixture based on PCL and shellac (ratio of PCL to shellac: 9:1) sealed the connection sites (Supplementary Fig. 46b). For connection between the flexible cable and the wireless module, zinc wires (diameter: 250 µm; Goodfellow Corporation, PA, USA) bonded to the contact pads in the flexible cable and in the wireless module with a bioresorbable conductive wax. Encapsulation used a natural wax mixture (ratio of Candelilla wax to Beeswax: 4:1).

### FEA analysis of wireless bioresorbable cardiac systems

FEA performed with commercial software (Abaqus; Dassault Systemes Simulia Corp., RI, USA) predicted the mechanical deformation and the electrical-to-mechanical coupling of the cardiac device. The bottom electrodes, floating electrodes, and top cover were modeled by 4-node shell elements, while the encapsulations and the connection sites were modeled by 8-node solid elements. Refined mesh with a size smaller than 5% of the electrode width was adopted to ensure accuracy. Two models were adopted for the cardiac tissues—the simplified model and the Living Heart Model. In the simplified model, the heart geometry was simplified as a cuboid (thickness: 10 mm)^69^, and the in-plane size was much larger than the device size. The materials of the cardiac tissues adopted the hyperelastic constitutive relationship, and the heart contraction was modeled via an equal biaxial stretching with uniform far-field strain. Supplementary Figs. 38 and 40a utilized the simplified model. As a more accurate model, the Living Heart Model^70^ (Dassault Systemes Simulia Corp., RI, USA), which was adopted and integrated with the device, including the precise geometries from 3D scanning, the anisotropic and strain-hardening constitutive relationship, and the electrical-to-mechanical coupled heart contraction. Supplementary Figs. 30e and 40b utilized the Living Heart Model.

### Characterization of the wireless bioresorbable cardiac system

Characterization tests evaluated the strain sensitivity, bending sensitivity, and effects of mechanical constraints on cardiac tissues through ex vivo evaluations. A vector network analyzer (E5063A) measured the S_11_ antenna parameter (reflection coefficient) of the three LC-type devices together in one system to determine the resonance frequency and Q factor of each. Device performance under stretching (strain: 0–10%) and bending (angle: 0–90°) was determined through the use of a custom mechanical stretcher. Quantitative imaging of a collection of black plastic particles (diameter: 1 mm) spread on the porcine heart revealed the effects of mechanical constraints imposed by the device on underlying cardiac tissues. 3D-particle tracking velocimetry (3D-PTV) through two digital cameras (2560 × 1600 pixels CMOS Phantom Miro 340 with 12 GB on-board memory and frame rates of 1000 f.p.s.) recorded the location of each particle as a function time during simulated cycles of beating. Pre-processing, calibration, 3D reconstruction, tracking, and post-processing exploited previous 3D PT codes^71^. Tracking the 3D reconstructed positions of these particles determined the deformation of the porcine hearts without and with the cardiac device, and with a rigid film for comparison. Ex vivo studies utilized porcine hearts with similar structures and dimensions to those of humans. An artificial heart system with a mechanical pump provided an adjustable and oscillatory pattern of flow (TS410 tubing module; Transonic Systems Inc., NY, USA) and pressure (Tru-wave disposable pressure transducer; Edwards Lifesciences, CA, USA) in the right ventricle (RV) of the porcine heart. Recordings of the deformation using the device mounted on the RV with three sutures on the end of each arm utilized the readout coil of the vector network analyzer (distance between the wireless module and the readout coil: ~5 mm).

### Animal evaluations of wireless bioresorbable cardiac systems

The animal procedures followed U.S. Department of Agriculture Animal Welfare Regulations at an accredited facility. Ovine model was utilized for this study. The multi-sensing element was mounted on the left ventricle (LV) of the heart, with the wireless module in the dorsal subcutaneous area. During the mounting process, a custom 3D-printed accessory grasped all three arms to prevent the device from bending or fracturing. The device was firmly attached to the LV with three suturing sites between the holes of each arm and the underlying tissues. The device continuously recorded the cardiac signals through a wireless readout coil connected to a vector network analyzer (E5063A). The signals were analyzed using a custom program (LabVIEW; National Instruments, TX, USA).

## Supplementary information

Supplementary Information

## Data Availability

The data that support the findings of this study are available from the corresponding author upon reasonable request.

## Code availability

The custom code used to collect and analyze S_11_ signals is available from the corresponding author upon reasonable request.

## References

[1] Hwang S-W (2012). A physically transient form of silicon electronics. Science.

[2] Li C (2020). Design of biodegradable, implantable devices towards clinical translation. Nat. Rev. Mater..

[3] Han WB, Lee JH, Shin J, Hwang S (2020). Advanced materials and systems for biodegradable, transient electronics. Adv. Mater..

[4] Hsu E, Barmak K, West AC, Park A-HA (2019). Advancements in the treatment and processing of electronic waste with sustainability: a review of metal extraction and recovery technologies. Green. Chem..

[5] Irimia-Vladu, M., Glowacki, E. D., Sariciftci, N. S. & Bauer, S. *Green Materials for Electronics*. (Wiley-VCH, 2017). 10.1039/c3tb20193g 32261127

[6] Global Pacemakers and Implantable Cardioverter Defibrillators (ICDs) Market—Analysis and Forecast, 2017–2023. (2017).

[7] Brain Monitoring Systems Market By Product (fNIRS, EEG, MEG, MRI), By Modality (Portable/Handheld, Standalone), By End User (Hospitals, Ambulatory Surgical Centers, Clinics, Pediatric & Neonatal Intensive Care Units) & Region—Forecast 2022–2028. (2022).

[8] Earle, N. *The Future of IoT*. (2022).

[9] Das, R., Chang, Y.-H. & Dyson, M. RFID Forecasts, Players and Opportunities 2022–2032. (2021).

[10] Kang S-K (2016). Bioresorbable silicon electronic sensors for the brain. Nature.

[11] Yu KJ (2016). Bioresorbable silicon electronics for transient spatiotemporal mapping of electrical activity from the cerebral cortex. Nat. Mater..

[12] Bai W (2019). Bioresorbable photonic devices for the spectroscopic characterization of physiological status and neural activity. Nat. Biomed. Eng..

[13] Koo J (2018). Wireless bioresorbable electronic system enables sustained nonpharmacological neuroregenerative therapy. Nat. Med..

[14] Choi YS (2021). Fully implantable and bioresorbable cardiac pacemakers without leads or batteries. Nat. Biotechnol..

[15] Koo, J. et al. Wirelessly controlled, bioresorbable drug delivery device with active valves that exploit electrochemically triggered crevice corrosion. *Sci. Adv.* **6**, (2020). 10.1126/sciadv.abb1093 PMC7455185 32923633

[16] Boutry CM (2019). Biodegradable and flexible arterial-pulse sensor for the wireless monitoring of blood flow. Nat. Biomed. Eng..

[17] Jamshidi R, Taghavimehr M, Chen Y, Hashemi N, Montazami R (2022). Transient electronics as sustainable systems: from fundamentals to applications. Adv. Sustain. Syst..

[18] Hwang S-W (2014). High-performance biodegradable/transient electronics on biodegradable polymers. Adv. Mater..

[19] Yu X, Shou W, Mahajan BK, Huang X, Pan H (2018). Materials, processes, and facile manufacturing for bioresorbable electronics: a review. Adv. Mater..

[20] Shou W (2017). Low‐cost manufacturing of bioresorbable conductors by evaporation–condensation‐mediated laser printing and sintering of Zn nanoparticles. Adv. Mater..

[21] Lee YK (2017). Room temperature electrochemical sintering of Zn microparticles and its use in printable conducting inks for bioresorbable electronics. Adv. Mater..

[22] Han WB, Ko G-J, Shin J-W, Hwang S-W (2020). Advanced manufacturing for transient electronics. MRS Bull..

[23] Huang X (2014). Biodegradable materials for multilayer transient printed circuit boards. Adv. Mater..

[24] MAIMAN TH (1960). Stimulated optical radiation in ruby. Nature.

[25] Joe DJ (2017). Laser-material interactions for flexible applications. Adv. Mater..

[26] Munoz-Garcia C (2022). Influence of wavelength and pulse duration on the selective laser ablation of WOx, VOx and MoOx thin films. Surf. Interfaces.

[27] Sundaram SK, Mazur E (2002). Inducing and probing non-thermal transitions in semiconductors using femtosecond laser pulses. Nat. Mater..

[28] Hermann S, Harder N-P, Brendel R, Herzog D, Haferkamp H (2010). Picosecond laser ablation of SiO2 layers on silicon substrates. Appl. Phys. A.

[29] Park JH (2019). Self-powered flexible electronics beyond thermal limits. Nano Energy.

[30] Keller WJ (2019). Physics of picosecond pulse laser ablation. J. Appl. Phys..

[31] Mustafa H, Matthews DTA, Römer GRBE (2022). Wavelength dependence of picosecond-pulsed laser ablation of hot-dip galvanized steel. Appl. Phys. A.

[32] Vidal F (2001). Critical-point phase separation in laser ablation of conductors. Phys. Rev. Lett..

[33] von der Linde D, Sokolowski-Tinten K (2000). The physical mechanisms of short-pulse laser ablation. Appl Surf. Sci..

[34] von der Linde D, Schüler H (1996). Breakdown threshold and plasma formation in femtosecond laser–solid interaction. J. Optical Soc. Am. B.

[35] Shin G (2017). Flexible near-field wireless optoelectronics as subdermal implants for broad applications in optogenetics. Neuron.

[36] Bian J (2019). Laser transfer, printing, and assembly techniques for flexible electronics. Adv. Electron. Mater..

[37] Kim, K. K., Choi, J., Kim, J., Nam, S. & Ko, S. H. Evolvable skin electronics by in situ and in operando adaptation. *Adv. Funct. Mater.* 2106329 10.1002/adfm.202106329 (2021).

[38] Yang Y (2020). A laser-engraved wearable sensor for sensitive detection of uric acid and tyrosine in sweat. Nat. Biotechnol..

[39] Wang M, Yang Y, Gao W (2021). Laser-engraved graphene for flexible and wearable electronics. Trends Chem..

[40] You R (2020). Laser fabrication of graphene‐based flexible electronics. Adv. Mater..

[41] Stuart T (2021). Biosymbiotic, personalized, and digitally manufactured wireless devices for indefinite collection of high-fidelity biosignals. Sci. Adv.

[42] Chichkov BN, Momma C, Nolte S, Alvensleben F, Tünnermann A (1996). Femtosecond, picosecond and nanosecond laser ablation of solids. Appl. Phys. A Mater. Sci. Process..

[43] Kumar V, Verma R, Kango S, Sharma VS (2021). Recent progresses and applications in laser-based surface texturing systems. Mater. Today Commun..

[44] Rogers JA, Someya T, Huang Y (2010). Materials and mechanics for stretchable electronics. Science.

[45] Kim D-H, Ghaffari R, Lu N, Rogers JA (2012). Flexible and stretchable electronics for biointegrated devices. Annu. Rev. Biomed. Eng..

[46] Wang Y, Shen N, Befekadu GK, Pasiliao CL (2017). Modeling pulsed laser ablation of aluminum with finite element analysis considering material moving front. Int. J. Heat. Mass Transf..

[47] Pou-Álvarez P (2021). Nanosecond, picosecond and femtosecond laser surface treatment of magnesium alloy: role of pulse length. Surf. Coat. Technol..

[48] Bogaerts A, Chen Z (2005). Effect of laser parameters on laser ablation and laser-induced plasma formation: a numerical modeling investigation. Spectrochim. Acta Part B Spectrosc..

[49] Neuenschwander, B. et al. Processing of metals and dielectric materials with ps-laserpulses: results, strategies, limitations and needs. in (eds. Niino, H., Meunier, M., Gu, B. & Hennig, G.) 75840R. 10.1117/12.846521 (2010).

[50] Liu HC, Mao XL, Yoo JH, Russo RE (1999). Early phase laser-induced plasma diagnostics and mass removal during single-pulse laser ablation of silicon. Spectrochim. Acta Part B Spectrosc..

[51] Xian J (2018). A simple model to predict machined depth and surface profile for picosecond laser surface texturing. Appl. Sci..

[52] Shin J (2019). Bioresorbable pressure sensors protected with thermally grown silicon dioxide for the monitoring of chronic diseases and healing processes. Nat. Biomed. Eng..

[53] Yang Q (2020). Materials, mechanics designs, and bioresorbable multisensor platforms for pressure monitoring in the intracranial space. Adv. Funct. Mater..

[54] Smit JM, Zeebregts CJ, Acosta R (2008). Timing of presentation of the first signs of vascular compromise dictates the salvage outcome of free flap transfers. Plast. Reconstr. Surg..

[55] Chao AH, Meyerson J, Povoski SP, Kocak E (2013). A review of devices used in the monitoring of microvascular free tissue transfers. Expert Rev. Med. Devices.

[56] Lee YK (2017). Dissolution of monocrystalline silicon nanomembranes and their use as encapsulation layers and electrical interfaces in water-soluble electronics. ACS Nano.

[57] Oskam G, Long JG, Natarajan A, Searson PC (1998). Electrochemical deposition of metals onto silicon. J. Phys. D. Appl Phys..

[58] Wang S (2013). Large-area free-standing ultrathin single-crystal silicon as processable materials. Nano Lett..

[59] Hwang S-W (2013). Materials and fabrication processes for transient and bioresorbable high-performance electronics. Adv. Funct. Mater..

[60] Chang J-K (2017). Materials and processing approaches for foundry-compatible transient electronics. Proc. Natl Acad. Sci..

[61] Dandel M, Lehmkuhl H, Knosalla C, Suramelashvili N, Hetzer R (2009). Strain and strain rate imaging by echocardiography—basic concepts and clinical applicability. Curr. Cardiol. Rev..

[62] Choi YS (2020). Stretchable, dynamic covalent polymers for soft, long-lived bioresorbable electronic stimulators designed to facilitate neuromuscular regeneration. Nat. Commun..

[63] Ferraiuoli P (2019). Full-field analysis of epicardial strain in an in vitro porcine heart platform. J. Mech. Behav. Biomed. Mater..

[64] Zhang H, Iijima K, Huang J, Walcott GP, Rogers JM (2016). Optical mapping of membrane potential and epicardial deformation in beating hearts. Biophys. J..

[65] Yin L, Bozler C, Harburg DV, Omenetto F, Rogers JA (2015). Materials and fabrication sequences for water soluble silicon integrated circuits at the 90 nm node. Appl Phys. Lett..

[66] Wandel MB (2021). Concomitant control of mechanical properties and degradation in resorbable elastomer-like materials using stereochemistry and stoichiometry for soft tissue engineering. Nat. Commun..

[67] Schneider CA, Rasband WS, Eliceiri KW (2012). NIH Image to ImageJ: 25 years of image analysis. Nat. Methods.

[68] Schindelin J (2012). Fiji: an open-source platform for biological-image analysis. Nat. Methods.

[69] Lee PT (2013). Left ventricular wall thickness and the presence of asymmetric hypertrophy in healthy young army recruits. Circ. Cardiovasc. Imaging.

[70] SIMULIA LIVING HEART https://www.3ds.com/products-services/simulia/solutions/life-sciences-healthcare/living-heart-human-model/.

[71] Kim J-T, Nam J, Shen S, Lee C, Chamorro LP (2020). On the dynamics of air bubbles in Rayleigh–Bénard convection. J. Fluid Mech..

