# Supplementary material for: High-speed, scanned laser structuring of multi-layered eco/bioresorbable materials for advanced electronic systems
Source: Nat Commun. 2022 Oct 31;13:6518. doi: 10.1038/s41467-022-34173-0 (PMC9622701; doi:10.1038/s41467-022-34173-0)
Supplement: Supplementary file 1 — Supplementary Information [file 41467_2022_34173_MOESM1_ESM.pdf]

Supplementary Information

# High-speed, scanned laser structuring of multi-layered eco/bioresorbable materials for advanced electronic systems

Quansan Yang[1,2†], Ziying Hu[1†], Min-Ho Seo[1,3,4], Yameng Xu[5], Ying Yan[6], Yen-Hao Hsu[7], Jaime Berkovich[3], Kwonjae Lee[8], Tzu-Li Liu[1,2], Samantha McDonald[7], Haolin Nie[9], Hannah Oh[10], Mingzheng Wu[10], Jin-Tae Kim[1], Stephen A. Miller[11], Ying Jia[12], Serkan Butun[12], Wubin Bai[1,13], Hexia Guo[1,3], Junhwan Choi[1], Anthony Banks[1], Wilson Z. Ray[6], Yevgenia Kozorovitskiy[10,14], Matthew L. Becker[7,15], Mitchell A. Pet[16], Matthew R. MacEwan[6], Jan-Kai Chang[1,17], Heling Wang[2,18,19]*, Yonggang Huang[2,3,20]*, John A. Rogers[1,2,3,9,21*]

[1]Querrey Simpson Institute for Bioelectronics, Northwestern University, Evanston, IL 60208, USA. [2]Department of Mechanical Engineering, Northwestern University, Evanston, IL 60208, USA. [3]Department of Materials Science and Engineering, Northwestern University, Evanston, IL 60208, USA [4]School of Biomedical Convergence Engineering, College of Information & Biomedical Engineering, Pusan National University, Pusan 46241, Republic of Korea. [5]The Institute of Materials Science and Engineering, Washington University in St. Louis, St. Louis, MO 63130, USA. [6]Department of Neurosurgery, Washington University School of Medicine in St. Louis, St. Louis, MO 63130, USA. [7]Department of Chemistry, Duke University, Durham, NC 27708, USA. [8]Department of Biological Sciences, Northwestern University, IL 60208, USA. [9]Department of Biomedical Engineering, Northwestern University, Evanston, IL 60208, USA. [10]Department of Neurobiology, Northwestern University, Evanston, IL 60208, USA. [11]Laser and Electronics Design Core Facility, Northwestern University, Evanston, IL 60208, USA. [12]Micro/Nano Fabrication Facility, Northwestern University, Evanston, IL 60208, USA. [13]Department of Applied Physical Sciences, University of North Carolina at Chapel Hill, Chapel Hill, NC 27599, USA. [14]Developmental Therapeutics Core, Northwestern University, Evanston, IL 60208, USA. [15]Department of Biomedical Engineering and Orthopaedic Surgery, Duke University, Durham, NC 27708, USA. [16]Division of Plastic and Reconstructive Surgery, Washington University School of Medicine in St. Louis, St. Louis, MO 63130, USA. [17]Wearifi Inc., Evanston, IL 60201, USA. [18]Center for Flexible Electronics Technology, Tsinghua University, Beijing 100084, China. [19]Zhejiang Tsinghua Institute of Flexible Electronics Technology, Jiaxing 314000, China. [20]Departments of Civil and Environmental Engineering, Northwestern University, Evanston, IL 60208, USA. [21]Department of Neurological Surgery, Feinberg School of Medicine, Northwestern University, Chicago, IL 60611, USA. [†]These authors contributed equally to this work. [*]To whom correspondence should be addressed to helingwang1@gmail.com (H.W.), y-huang@northwestern.edu (Y.H.), and jrogers@northwestern.edu (J.A.R.).

Content

Supplementary Note 1. Challenges and limitations in current microfabrication technologies for bioresorbable electronics ........ 5

Supplementary Note 2. Processing by thickness reduction for resistive-type bioresorbable devices........ 7

Supplementary Note 3. Theory for converting temperature differences to flow rate for bioresorbable microvascular sensing probes ........ 8

Supplementary Note 4. Alternative laser process for multi-layer electronic architectures. ........ 9

Supplementary Note 5. LabVIEW capture and fitting programs ........ 10

Supplementary Note 6. Theory for converting resonance frequency to strain for wireless bioresorbable cardiac electronic systems........ 12

Supplementary Table 1 | Comparisons among fabrication technologies for bioresorbable electronics, including the proposed laser ablation method and two widely used strategies (photolithography-based microfabrication and solution-based additional manufacturing)........ 14

Supplementary Figure 1 | Current fabrication steps for bioresorbable electronics........ 16

Supplementary Figure 2 | Cross-sectional schematic illustrations of laser ablation procedures for advanced bioresorbable electronics, corresponding to Fig. 1a-c. ........ 17

Supplementary Figure 3 | Representative laser-processed bioresorbable device types include mono-layer thinned and ablated devices, multi-layer ablated devices, and integrated systems, with features including dry processing, minimum heat generation, precise thickness reduction, excellent resolution, and multi-layer design. ........ 18

Supplementary Figure 4 | Simulation results for controlled thickness reduction in monocrystalline Si MM by tuning the laser parameters, corresponding to Fig. 2a-c........ 19

Supplementary Figure 5 | Experimental and simulation results for controlled thickness reduction in Mg by tuning the laser parameters........ 20

Supplementary Figure 6 | Schematic illustrations of key parameters used for ablation using ultrashort pulsed lasers........ 21

Supplementary Figure 7 | Method resolution in thickness reduction (~ 35 nm)........ 22

Supplementary Figure 8 | Simulations of the thermal diffusion zone induced by a body heat flux with durations *t* from 10 ns to 100 fs. ........ 24

Supplementary Figure 9 | Gaussian distribution of the power density along the radial direction of the laser spot (diameter: ~ 15 μm). ........ 25

Supplementary Figure 10 | Simulation results for the cross-sectional profiles of Si ribbons patterned in three cases, corresponding to Fig. 2g........ 26

Supplementary Figure 11 | Characteristic feature sizes of a monocrystalline Si ribbon patterned on a PLA substrate. ........ 27

Supplementary Figure 12 | Quantitative characterization of a trench shape formed by laser method........ 28

Supplementary Figure 13 | Schematic illustration and optical micrograph of the pattern for characterizing overlay registration, corresponding to Fig. 2j. .................................................... 29

Supplementary Figure 14 | Laser ablation method for surface treatment on substrates. ............. 30

Supplementary Figure 15 | SEM characterization of laser-ablated Si and Mg structures. ........... 31

Supplementary Figure 16 | Capability for forming arrays of devices over large areas. ............... 32

Supplementary Figure 17 | Laser ablation strategy for bioresorbable inductors at the cm- and sub-mm scales. ........................................................................................................................... 33

Supplementary Figure 18 | Laser thinning strategy for bioresorbable resistive-type devices. ..... 34

Supplementary Figure 19 | Laser patterning of Zn (a) and Mo (b) on CA substrates into flexible sensing arrays. ........................................................................................................................ 35

Supplementary Figure 20 | Laser thinning and cutting of non-eco/bioresorbable polymers, including polyimide (PI) and polydimethylsiloxane (PDMS), into probe shapes. ....................... 36

Supplementary Figure 21 | Bioresorption of laser-fabricated devices. ........................................ 37

Supplementary Figure 22 | Biocompatibility of bioresorbable sensing probes formed by laser ablation. .............................................................................................................................. 38

Supplementary Figure 23 | Laser ablation strategy for Si-based resistive-type devices. ............. 39

Supplementary Figure 24 | Laser ablation strategy for forming Si-based capacitive-type devices. ......................................................................................................................................... 40

Supplementary Figure 25 | Schematic illustration of an alternative process for forming multi-layer Si-based electrode arrays. ........................................................................................ 41

Supplementary Figure 26 | Equivalent circuit model used to fit the electrochemical impedance spectra of Si-based electrodes. ........................................................................................ 42

Supplementary Figure 27 | Multi-layer laser ablation strategy for Si-based temperature sensing arrays. .............................................................................................................................. 43

Supplementary Figure 28 | Additional information for multi-layer Si-based n-channel MOSFETs fabricated by laser methods, corresponding to Fig. 5g-i, including top view (a), cross-sectional view (b), 3D exploded view (c), and optical micrograph (d) of the MOSFET devices............... 44

Supplementary Figure 29 | Multi-layer laser ablation strategy for Si-based diodes. .................... 46

Supplementary Figure 30 | Device architectures and working principles of the multi-sensing element. ........................................................................................................................... 48

Supplementary Figure 31 | Tissue-like mechanical and water barrier encapsulation properties of the WPU layer. ............................................................................................................... 49

Supplementary Figure 32 | Customized 3D-printed accessory to hold the device in a manner that avoids bending and fracture during the surgery. ........................................................... 50

Supplementary Figure 33 | Equivalent circuit model for simultaneous wireless measurements.. 51

Supplementary Figure 34 | Flow chart for a custom LabVIEW program for real-time data collection and analysis and subsequent further analysis. ............................................................ 52

Supplementary Figure 35 | Custom LabVIEW program for real-time data collection and analysis and subsequent further analysis. .......................................................................................... 54
Supplementary Figure 36 | Coordinate transformation to convert the strains along 0, 120, and 240° for each arm to principal strains ($\varepsilon_x$ and $\varepsilon_y$) and shear strain ($\gamma_{xy}$). ..................................... 55
Supplementary Figure 37 | Strain sensitivity and bending insensitivity of the multi-sensing element. ....................................................................................................................... 56
Supplementary Figure 38 | Simulation results for the sensitivity to strain and bending. ............. 57
Supplementary Figure 39 | Insignificant mechanical constraints on natural motions of the cardiac tissues. ....................................................................................................................... 59
Supplementary Figure 40 | Results using a simplified model and a living heart model on the constraints to natural motions of cardiac tissues............................................................ 61
Supplementary Figure 41 | *Ex vivo* evaluations of the cardiac device on an artificial heart system with adjustable oscillatory flow and pressure. .............................................................. 62
Supplementary Figure 42 | Principal and shear strains in *ex vivo* evaluations of the cardiac device. ....................................................................................................................... 63
Supplementary Figure 43 | Raw data for the three units in a representative segment (duration: 20 s). ....................................................................................................................... 64
Supplementary Figure 44 | Data filtering for raw data measured from the LV of the porcine heart. ....................................................................................................................... 65
Supplementary Figure 45 | Quantitative assessment of the strains of the ovine LV along the three arms, corresponding to Fig. 6j-l. ......................................................................................... 66
Supplementary Figure 46 | Connection between the multi-sensing element and the flexible cable. ....................................................................................................................... 67
References ....................................................................................................................... 68

**Supplementary Note 1. Challenges and limitations in current microfabrication technologies for bioresorbable electronics**

Existing schemes for fabricating bioresorbable electronic devices mainly rely on (1) conventional (e.g., photolithography) processes in microfabrication and (2) solution-based additive manufacturing. The comparisons between these technologies and the laser ablation method appear in Supplementary Table 1.

Photolithography is the most commonly used method to pattern thin film materials with high resolution (~ 1 μm) and alignment accuracy (~ 1 μm). A challenge is that most bioresorbable materials degrade during key steps in this process. For example, aqueous developers used to remove photoresists can partially dissolve bioresorbable metals and other water-soluble constituent materials. Additionally, most bioresorbable polymers, such as PLGA and PCL, are vulnerable to chemicals and thermal cycles required for the use of photoresists. As a result, existing schemes rely on processing on inert substrates (such as Si) followed by transfer printing to a polymer substrate[1–3], as shown in Supplementary Fig. 1. For example, patterning films of Mg starts with sequential spin-coating a layer of poly(methyl methacrylate) (PMMA) and a supporting film of polyimide (PI) in sequence, followed by vacuum deposition of a uniform layer of Mg on top. To minimize direct contact of Mg with developers, the patterning uses protective layers of Fe that are subsequently removed by wet etching. Spin-coating another PI layer on the top yields a tri-layer PI/Mg/PI structure on PMMA/Si substrate. The transfer printing starts with immersion in acetone to dissolve the PMMA and release the tri-layer structure for transfer to a stamp of polydimethylsiloxane (PDMS). The top and bottom PI act as mechanical supporting layers, with

the central Mg structure at the neutral mechanical plane. The sample is then transferred to a bioresorbable polymer substrate after reactive ion etching (RIE) to remove the bottom layer of PI. A second RIE step removes the top layer of PI to complete the fabrication.

An alternative relies on solution-based additive manufacturing methods[4–12] such as screen printing, stencil printing, microcontact printing, digital printing, and others. Most strategies employ metal-nanoparticles or conductive polymers printed and sintered (heat, laser, and electro-chemical sintering) on inert substrates (*e.g.*, Si and glass) and then transferred to polymer substrates. Although conceptually simple, such schemes have many challenges and limitations with transient conductors. Certain metals such as Zn work well due to the low melting point for sintering and strong photonic absorption. Patterning Mg in this manner is difficult, however, due to its strong chemical activity. Limitations with W and Mo follow from their high melting points (W, 3422 °C; Mo, 2623 °C). Other barriers with Fe arise from its low electronegativity, and its incompatibility with many semiconductor materials. The conductivity of the resulting patterned traces depends strongly on the size and morphology of the micro/nanoparticles, their physical contact, and the type of solvents. Low resolution in lateral patterning (~ 40 μm) and difficulty in thickness control are two other limitations of existing methods.

**Supplementary Note 2. Processing by thickness reduction for resistive-type bioresorbable devices.**

Thickness reduction by ablation can also be utilized for sensing elements in resistive-type devices (material: Zn/CA; thickness: 25/35 μm). In one example, this process locally reduces the thickness of a metal trace from 25 to 5 – 6 μm to create a highly resistive segment for sensing and low resistive regions for efficient electrical interconnection, shown in Supplementary Fig. 18a. Supplementary Fig. 18b shows that as the thickness of the sensing element reduces from 25 to 5 – 6 μm, the resistance of the entire device increases ~ 3.5 times than the initial value.

**Supplementary Note 3. Theory for converting temperature differences to flow rate for bioresorbable microvascular sensing probes**

The conversion of temperature to flow rate uses a previously reported equation[14,15] as follows

$$\Delta T = \frac{\frac{qR}{k}}{1+0.76s\sqrt{\frac{\mu R}{\alpha}}} F\left(\frac{r}{R}\right),$$

where $\Delta T$ is the temperature difference between two thermistors, $q$ is the heat flux of the heater, $R$ is the radius of the heater (1.34 mm; assuming the heater is a perfect circle), $k$ is the effective thermal conductivity of biological tissue, $r$ is the distance of these thermistors to the heater, $s$ is the capillary volume fraction in the tissue (~ 4% for muscle), $\mu$ is the blood flow rate in the tissue capillaries, $\alpha$ is the blood thermal diffusivity (~ 0.1 mm$^2$ s$^{-1}$), and

$$F\left(\frac{r}{R}\right) = \int_0^{\infty} J_0\,(\lambda r) J_1(\lambda R) \frac{d\lambda}{\lambda},$$

where $J_0$ and $J_1$ are Bessel functions of the first kind for integer orders of 0 and 1, respectively.

**Supplementary Note 4. Alternative laser process for multi-layer electronic architectures.**

As an alternative to multiple cycles of material preparation-patterning, multi-layer silicon-based electronic architectures can be formed by laser ablation after a single step of materials preparation. A representative example is in the fabrication of flexible Si-based bioresorbable multiplexed electrode arrays for ECoG recording, where Si MMs serve as sensing components and films of Mg as interconnection traces. As illustrated in Supplementary Fig. 25, this alternative process starts with preparation of layers of Si and Mg by transfer printing a highly n-doped Si MM (thickness: 2 μm), as shown in step (1), followed by vacuum deposition of a uniform layer of Mg (thickness: 1 μm) on top, as illustrated in step (2). Ablation patterns the top Mg into interconnection traces, as shown in step (3), then continues to pattern the Si MM into electrode shapes (number: 12; dimensions of the electrode pad: 300 × 300 μm; sensing area in total: 2 × 2 mm) with interconnection sites (trace width: 120 μm), and finally cuts the PLA substrate into the shape of a ribbon, as illustrated in step (4). The advantage of this alternative is to minimize offsets in overlay registration. This scheme is not, however, effective for device architectures that require some part of the top functional layer to directly contact the substrate.

## Supplementary Note 5. LabVIEW capture and fitting programs

Custom LabVIEW programs were developed for real-time data collection and analysis and data analysis. The data collection and analysis flow appear in Supplementary Fig. 34. A custom LabVIEW program, shown in Supplementary Fig. 35a allowed the collection and analysis of the reflection coefficient ($S_{11}$) determined with the ENA network analyzer, in real time. The $S_{11}$ data are first converted to the real part of the impedance (*Re(Z)*) according to

$$Re(Z) = Z_0 \frac{1 + S_{11}}{1 - S_{11}},$$

where $Z_0$ is determined as 50 Ω. The LabVIEW program presents *Re(Z)* and $|S_{11}|$ as a function of frequency, as well as the $S_{11}$ in a polar plot. *Re(Z)* exhibits three peaks for three sensing units in a sensing element. In parallel with the data collection, each of these peaks is defined into three separate frequency ranges. Each peak was fitted according to the following equation using the Levenberg-Marquardt algorithm:

$$\Delta Re(Z) = \frac{Af^2 + Bf + C}{\frac{f_s}{Q}\left[1 + Q^2\left(\frac{f}{f_s} - \frac{f_s}{f}\right)^2\right]} + D,$$

where $f$ is the frequency, $f_s$ is resonance frequency, $Q$ is quality factor, $D$ is the offset from zero, and $A$, $B$, and $C$ are related to the magnitude of each peak and are used to scale the fitting function. $f_s$ and $Q$ are the parameters of interest and are plotted as a function of time, respectively, by the

LabVIEW program. The program allows the operators to check the data in real time, albeit with lower time resolution. The LabVIEW real-time fitting algorithm (maximum data frequency: ~ 5 Hz) operates more slowly than the data collection (maximum data frequency: ~ 25 Hz). As a result, another version of the fitting program is utilized for post-processing all the data, shown in Supplementary Fig. 35b. This version is identical to the original algorithm except that every data spectrum can be fitted.

**Supplementary Note 6. Theory for converting resonance frequency to strain for wireless bioresorbable cardiac electronic systems**

The relationship between the resonance frequency, wirelessly measured by the vector network analyzer, and the strain is described in the following. The resonance frequency $f$ is related to the inductance $L$ of the electric circuit and total capacitance $C_{\text{total}}$ via $f = \frac{1}{2\pi}\sqrt{\frac{1}{LC_{\text{total}}}}$. During *in vivo* evaluations, the total capacitance consists of three parts, *i.e.*, the device capacitance $C$, the air capacitance $C_{\text{air}}$, and the capacitance from biological fluids and tissues $C_{\text{biology}}$, such that the equation is

$$f = \frac{1}{2\pi}\sqrt{\frac{1}{L(C + C_{\text{air}} + C_{\text{biology}})}}.$$

In the reference state without strain ($\varepsilon = 0$), the floating electrode fully overlaps with the bottom electrode (maximum overlapping area) and the device capacitance $C$ has a maximum value ($C_{\text{max}}$). In the fully stretched state ($\varepsilon = \varepsilon_{\text{max}}$), the edge of the floating electrode moves to that of the bottom electrode such that the overlapping area is zero, and the device capacitance is $C_{\text{min}}$. In a general case, when $0 \le \varepsilon \le \varepsilon_{\text{max}}$, the device capacitance is linearly related to the strain $\varepsilon$ via

$$C = C_{\text{max}} + \frac{C_{\text{min}} - C_{\text{max}}}{\varepsilon_{\text{max}}}\varepsilon.$$

The geometry of the floating electrode and the fixed bottom electrodes gives $\varepsilon_{\text{max}} = 0.18$ and FEA simulation gives $C_{\text{min}}/C_{\text{max}} = 0.2$. With $f_{\text{max}}$ denoting the resonance frequency in the fully overlapping state, the relationship between $f$ and $\varepsilon$ is

$$\frac{f}{f_{\text{max}}} = \sqrt{\frac{C_{\text{max}} + C_{\text{air}} + C_{\text{biology}}}{C + C_{\text{air}} + C_{\text{biology}}}} = 1 - \frac{C_{\text{min}} - C_{\text{max}}}{2(C_{\text{max}} + C_{\text{air}} + C_{\text{biology}})} \frac{\varepsilon}{\varepsilon_{\text{max}}}.$$

Taylor's expansion with respect to $\varepsilon$ is applied to derive the second equation above. Measuring the resonance frequency in the fully overlapping state ($f_{\text{max,air}}$) and the fully stretched state ($f_{\text{min,air}}$) in the air ($C_{\text{biology}} = 0$) calibrates $C_{\text{air}}$, *i.e.*,

$$C_{\text{air}} = \frac{f_{\text{max,air}}^2 C_{\text{max}} - f_{\text{min,air}}^2 C_{\text{min}}}{f_{\text{min,air}}^2 - f_{\text{max,air}}^2}.$$

By mounting the device on the heart in the fully overlapping state, $f_{\text{max}}$ can be measured and used to calibrate $C_{\text{biology}}$, *i.e.*,

$$C_{\text{biology}} = -\frac{f_{\text{max,air}}^2 C_{\text{max}} - f_{\text{max}}^2 C_{\text{max}} + f_{\text{max,air}}^2 C_{\text{air}} - f_{\text{max}}^2 C_{\text{air}}}{f_{\text{max,air}}^2}.$$

## Supplementary Table

| | Photolithography-based microfabrication | Solution-based additional manufacturing | High-speed, scanned laser ablation methods |
|---|---|---|---|
| Type | Wet process | Wet process | Dry process |
| Additional steps | Transfer printing | Sintering and transfer printing | \ |
| Resolution | ~ 1 μm | ~ 40 μm | ~ 5 μm |
| Alignment accuracy | ~ 1 μm | \ | 2 – 3 μm |
| Patterning speed with precise features | Highly dependent on proficiency level | \ | ~ 10 – 30 sec $cm^{-2}$ |
| Other limitations | • Material incompatibility<br>• Complicated procedures | • Effectiveness only on Zn<br>• Chemical sensitivity on solvents<br>• Purity of metal particles<br>• Difficulty in thickness control | \ |

**Supplementary Table 1 | Comparisons among fabrication technologies for bioresorbable electronics, including the proposed laser ablation method and two widely used strategies (photolithography-based microfabrication and solution-based additional manufacturing).**

# Supplementary Figures

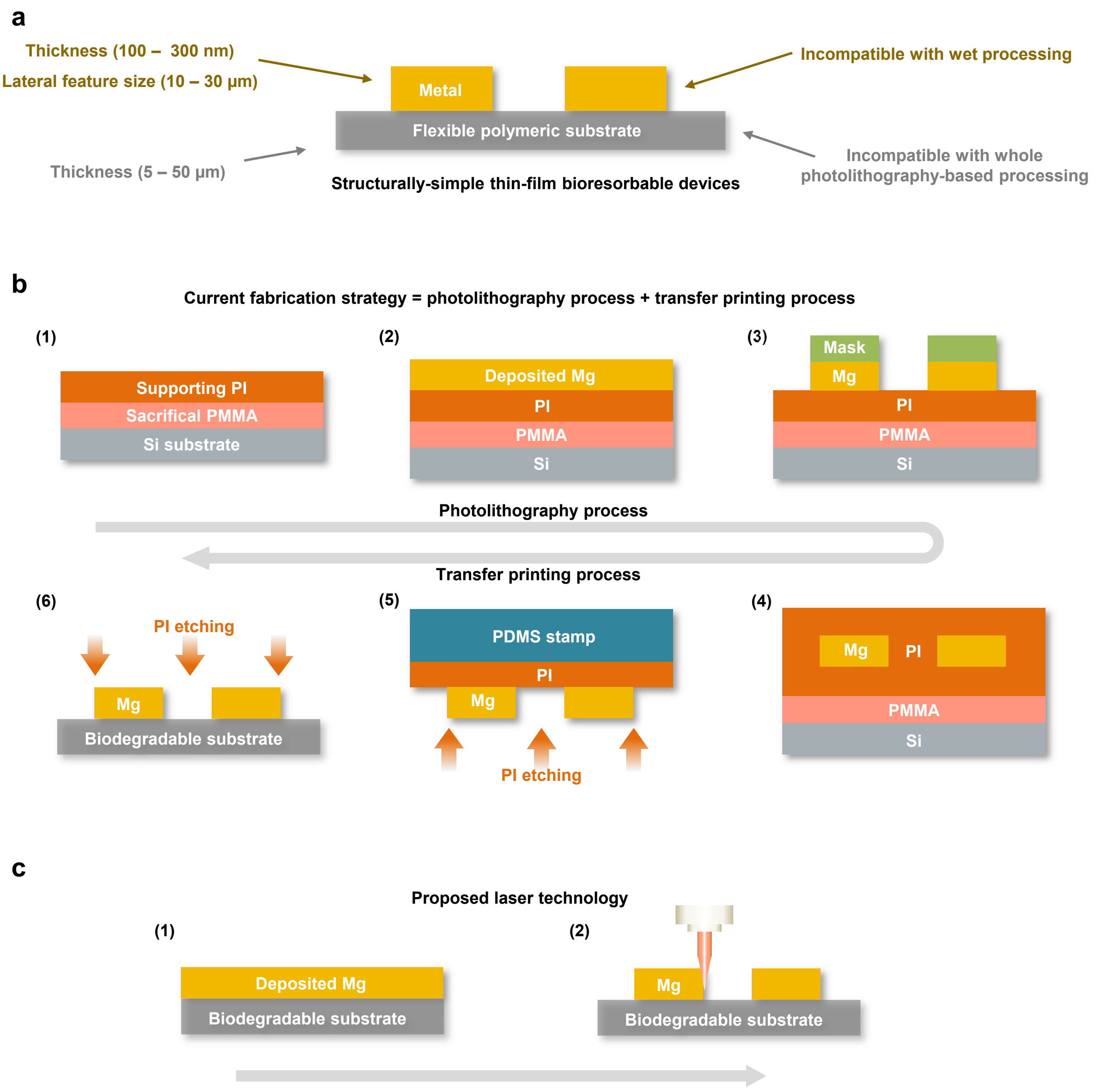

**Supplementary Figure 1 | Current fabrication steps for bioresorbable electronics. a,** Structurally-simple thin-film bioresorbable devices include a top functional metal layer (thickness: 100 – 300 nm; lateral feature size: 5 – 30 μm) and a flexible polymer substrate (thickness: 5 – 50 μm). A challenge is that the metal is incompatible with wet processing, and the bottom polymer substrate is incompatible with photolithography-based processing. **b,** Current fabrication scheme includes photolithography-based microfabrication and transfer printing of the patterned devices. The specific procedures consist of (1) spin-coating layers of supporting PI and sacrificial PMMA on a Si substrate, (2) vacuum deposition of a uniform layer of Mg, (3) patterning of a mask layer by photoresist (e.g., Fe), patterning of the Mg layer using diluted HCl, and removing the mask layer, (4) spin-coating another supporting PI layer, (5) releasing the sacrificial PMMA, retrieving the PI-Mg-PI tri-layer structure, and performing RIE on the bottom PI, and (6) transferring to the bioresorbable polymer substrate and performing RIE on the top PI. **c,** The proposed laser fabrication scheme introduced here includes (1) vacuum deposition of a uniform layer of Mg directly on a bioresorbable polymer substrate and (2) laser ablation of the Mg layer into desired patterns.

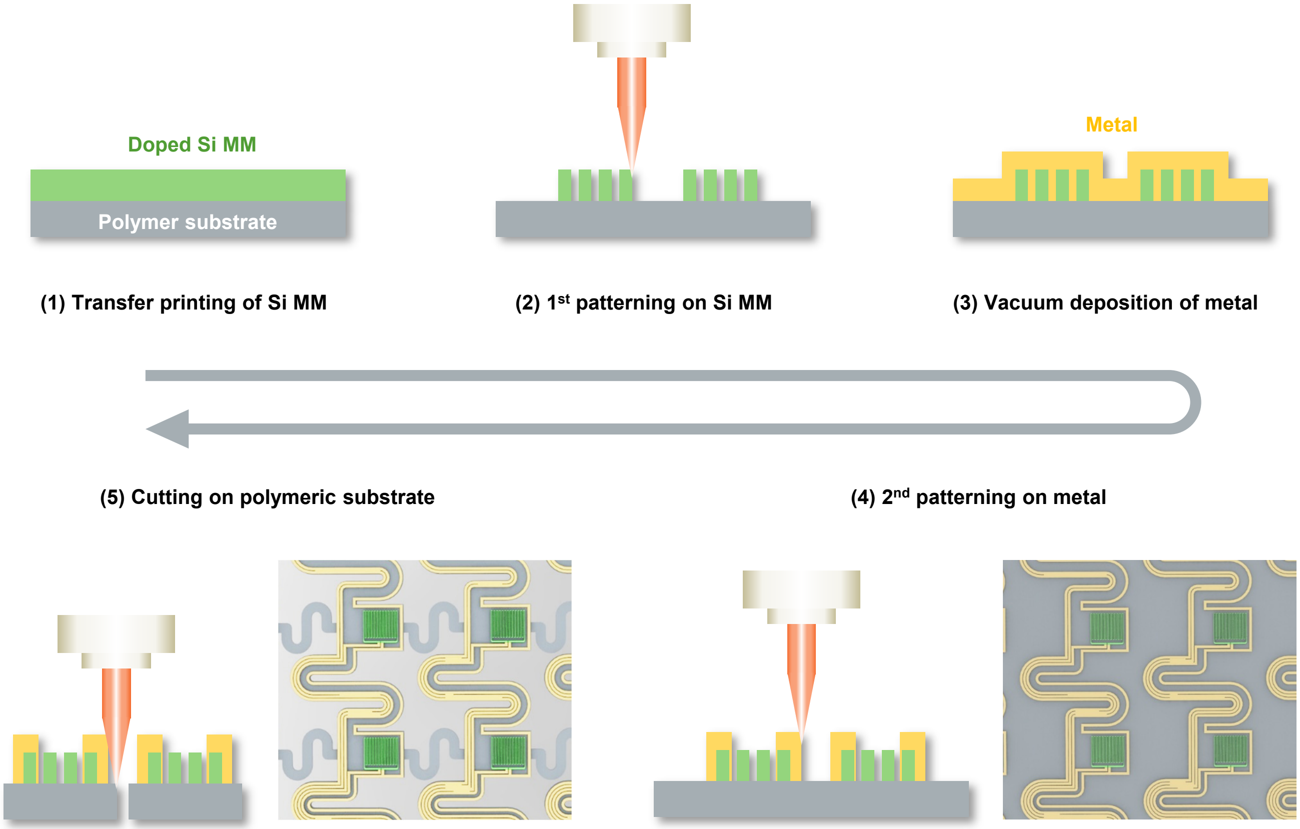


**Supplementary Figure 2 | Cross-sectional schematic illustrations of laser ablation procedures for advanced bioresorbable electronics, corresponding to Fig. 1a-c.**

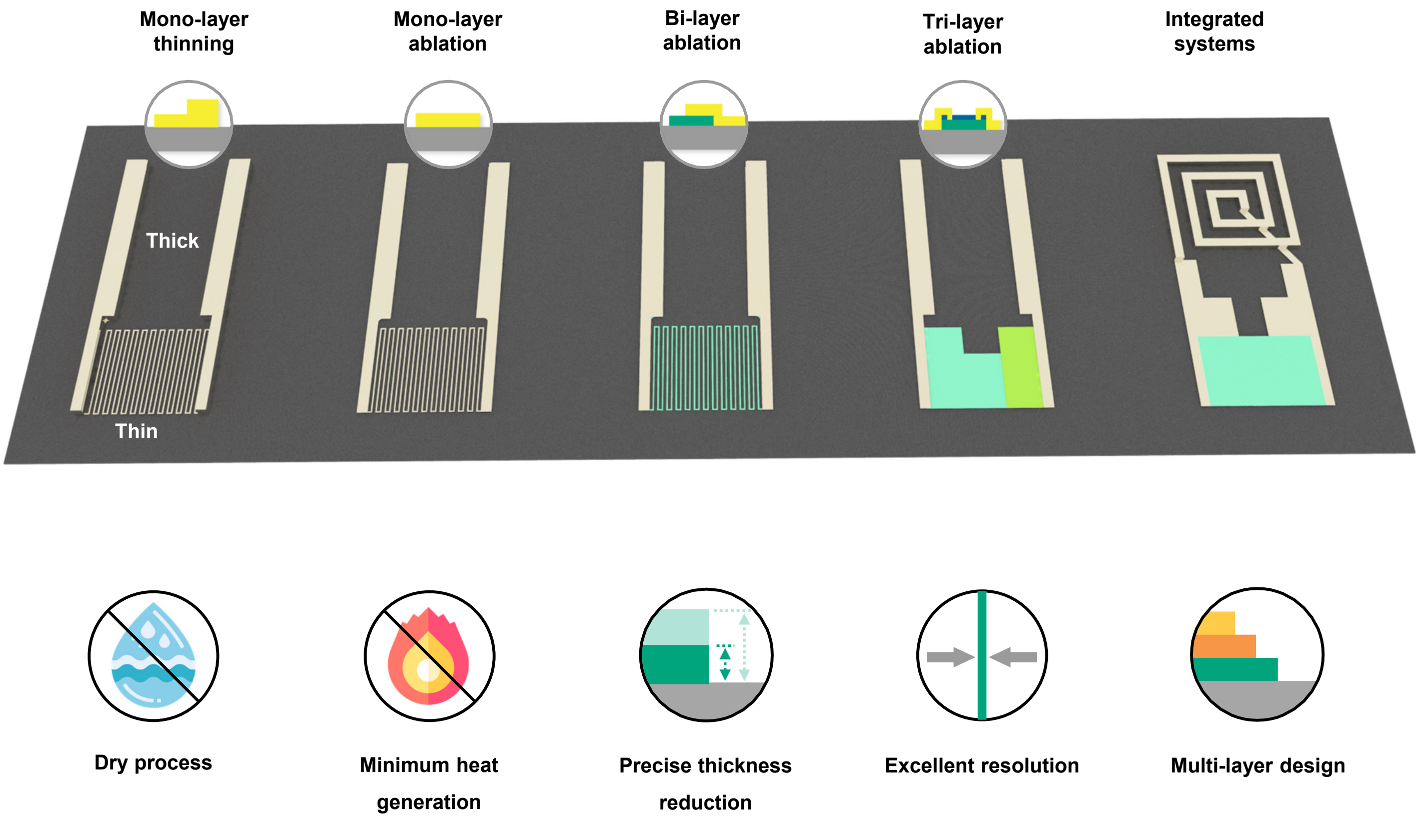


**Supplementary Figure 3 | Representative laser-processed bioresorbable device types include mono-layer thinned and ablated devices, multi-layer ablated devices, and integrated systems, with features including dry processing, minimum heat generation, precise thickness reduction, excellent resolution, and multi-layer design.**

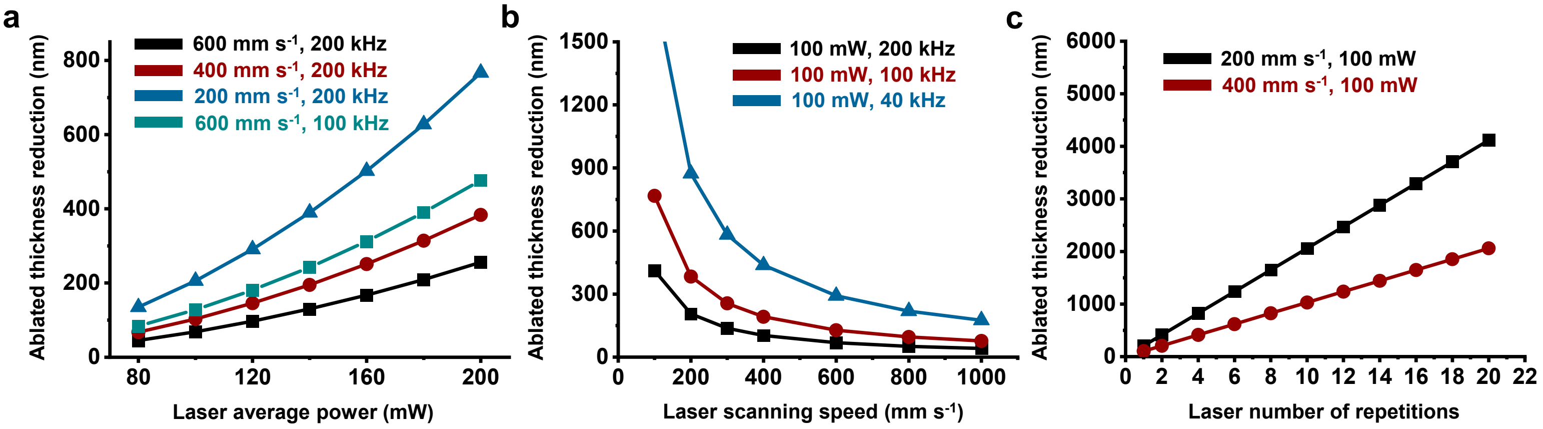


**Supplementary Figure 4 | Simulation results for controlled thickness reduction in monocrystalline Si MM by tuning the laser parameters, corresponding to Fig. 2a-c.** The laser parameters include average power (**a**), scanning speed (**b**), frequency (**b**), and the number of repetitions (**c**). The results are consistent with experimental measurements.

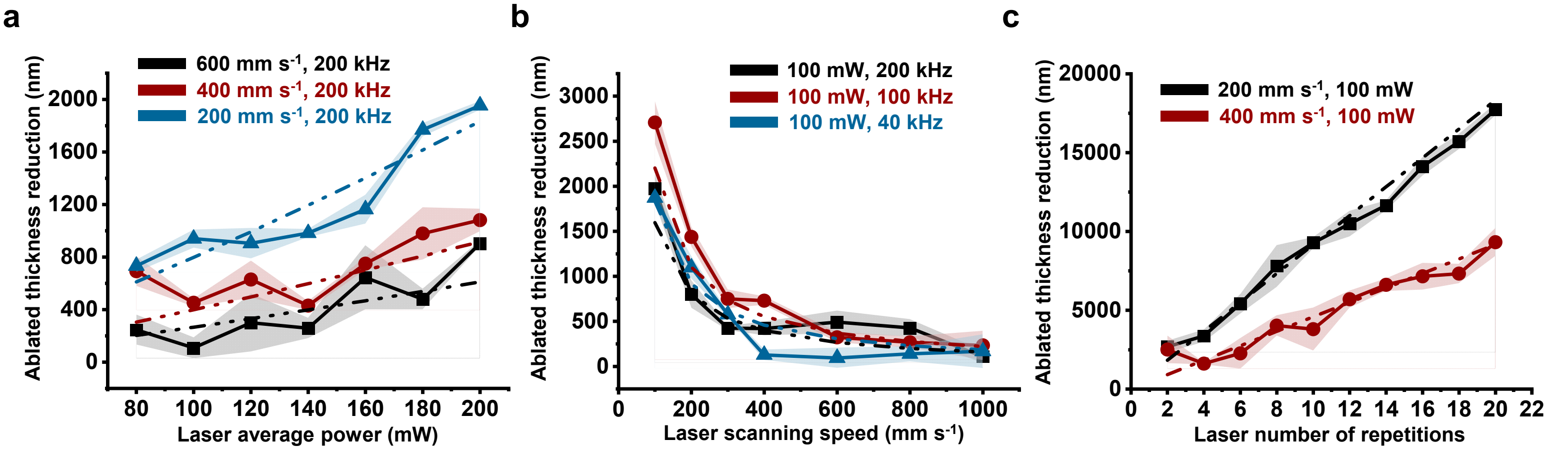


**Supplementary Figure 5 | Experimental and simulation results for controlled thickness reduction in Mg by tuning the laser parameters.** The laser parameters include average power (**a**), scanning speed (**b**), frequency (**b**), and the number of repetitions (**c**). Simulation results (dash-dotted lines) are consistent with experimental measurements. The shaded areas denote the standard deviation.

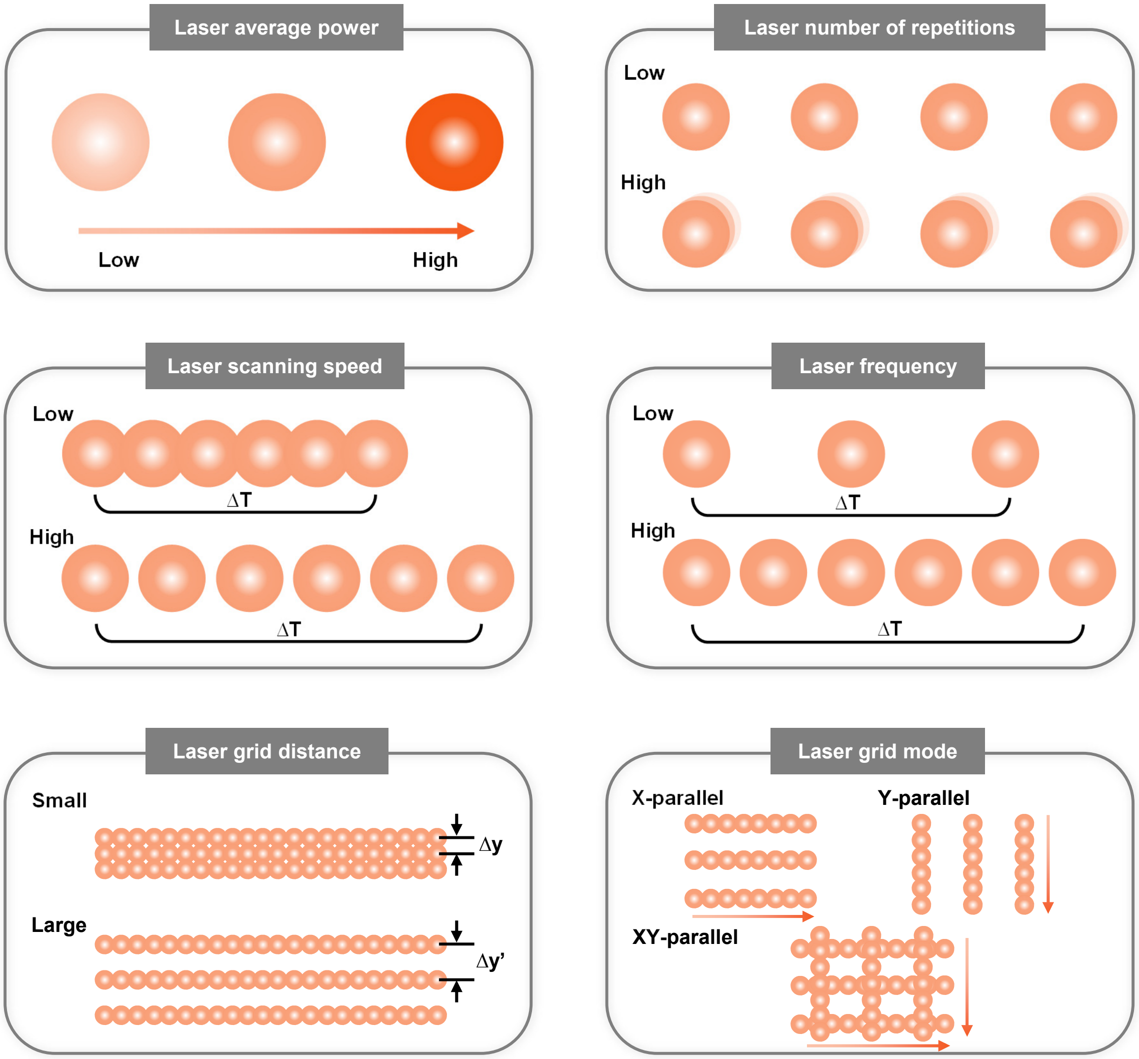


**Supplementary Figure 6 | Schematic illustrations of key parameters used for ablation using ultrashort pulsed lasers.**

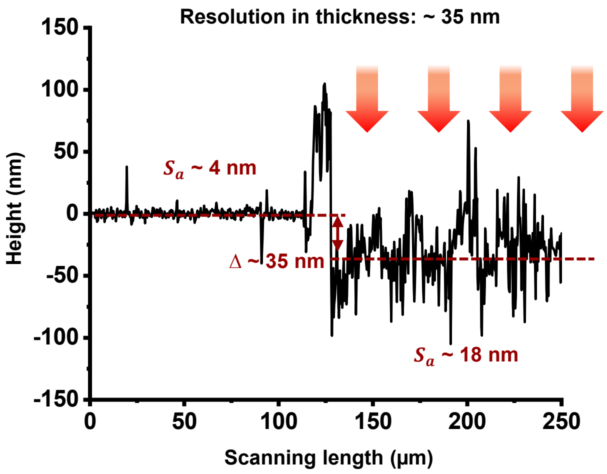


**Supplementary Figure 7 | Method resolution in thickness reduction (~ 35 nm).**

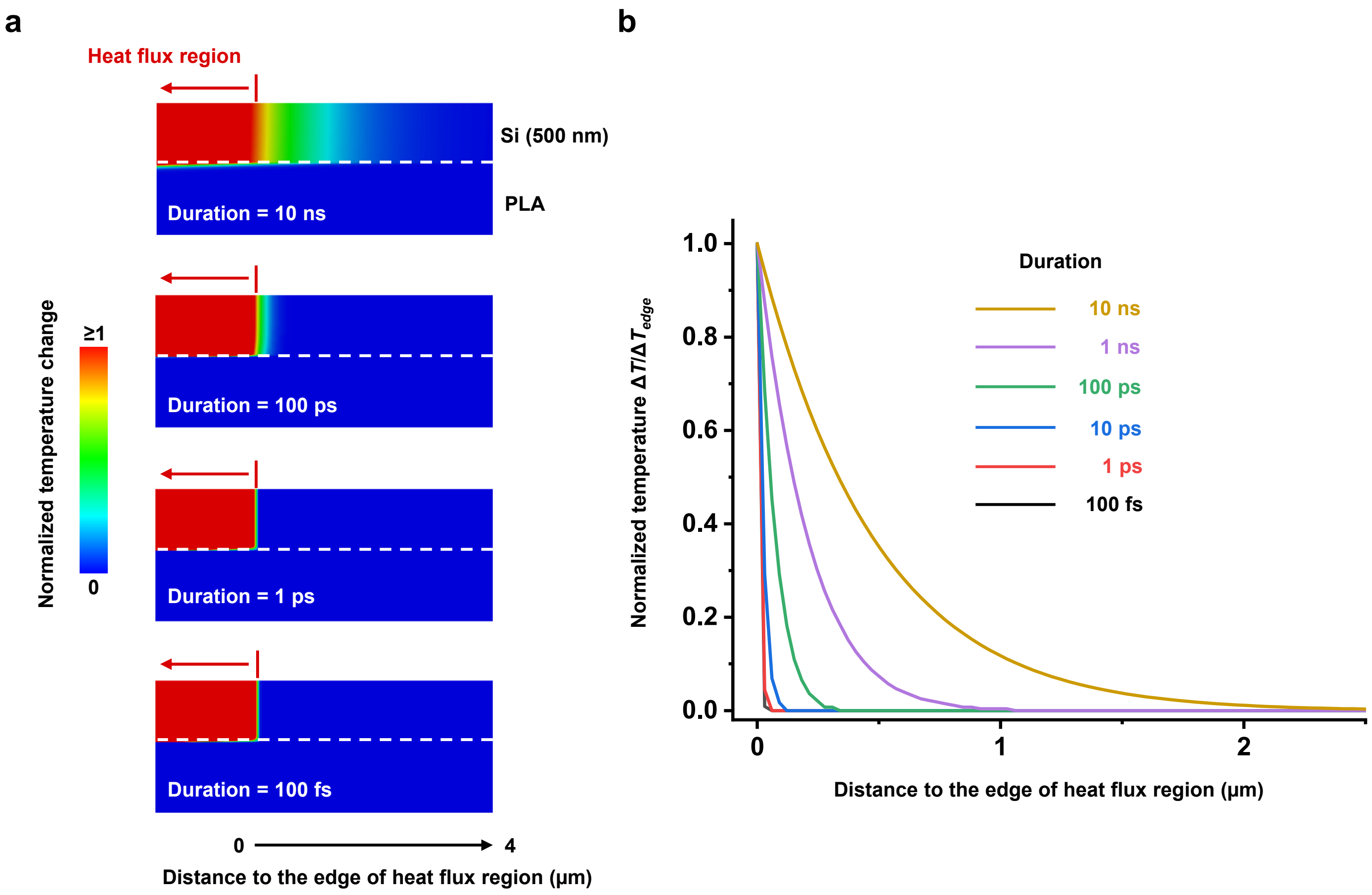


c

| Duration | Length scale |
|---|---|
| 10 ns | 500 nm |
| 1 ns | 140 nm |
| 100 ps | 45 nm |
| 10 ps | 15 nm |
| 1 ps | 5 nm |
| 100 fs | 5 nm |

**Supplementary Figure 8 | Simulations of the thermal diffusion zone induced by a body heat flux with durations *t* from 10 ns to 100 fs. a,** Normalized temperature distribution on a Si/PLA (thickness: 500 nm/50 μm) structure at time *t*. The temperature values are normalized by the temperature at the edge of the heat flux region. The results from top to bottom are the thermal diffusion zones associated with durations of 10 ns, 100 ps, 1 ps, and 100 fs, respectively. **b,** Normalized temperature as a function of the distance to the edge of the heat flux region for different durations. Durations that are less than 1 ps minimize heat transfer to the surrounding areas. **c,** Length scale values with different durations, which highlights that the ultrashort pulsed laser efficiently minimizes the thermal diffusion zone adjacent to the edges of patterned features.

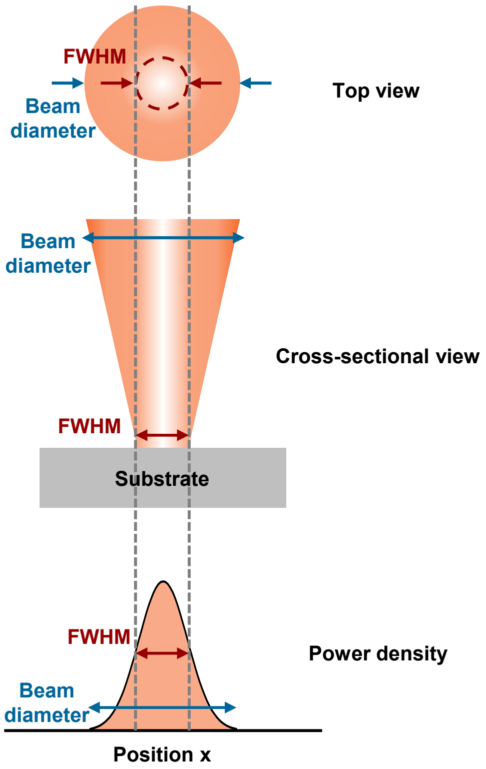


**Supplementary Figure 9 | Gaussian distribution of the power density along the radial direction of the laser spot (diameter: ~ 15 μm).**

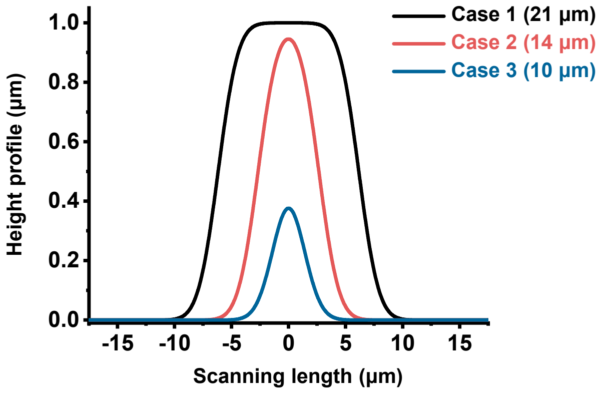


**Supplementary Figure 10 | Simulation results for the cross-sectional profiles of Si ribbons patterned in three cases, corresponding to Fig. 2g.**

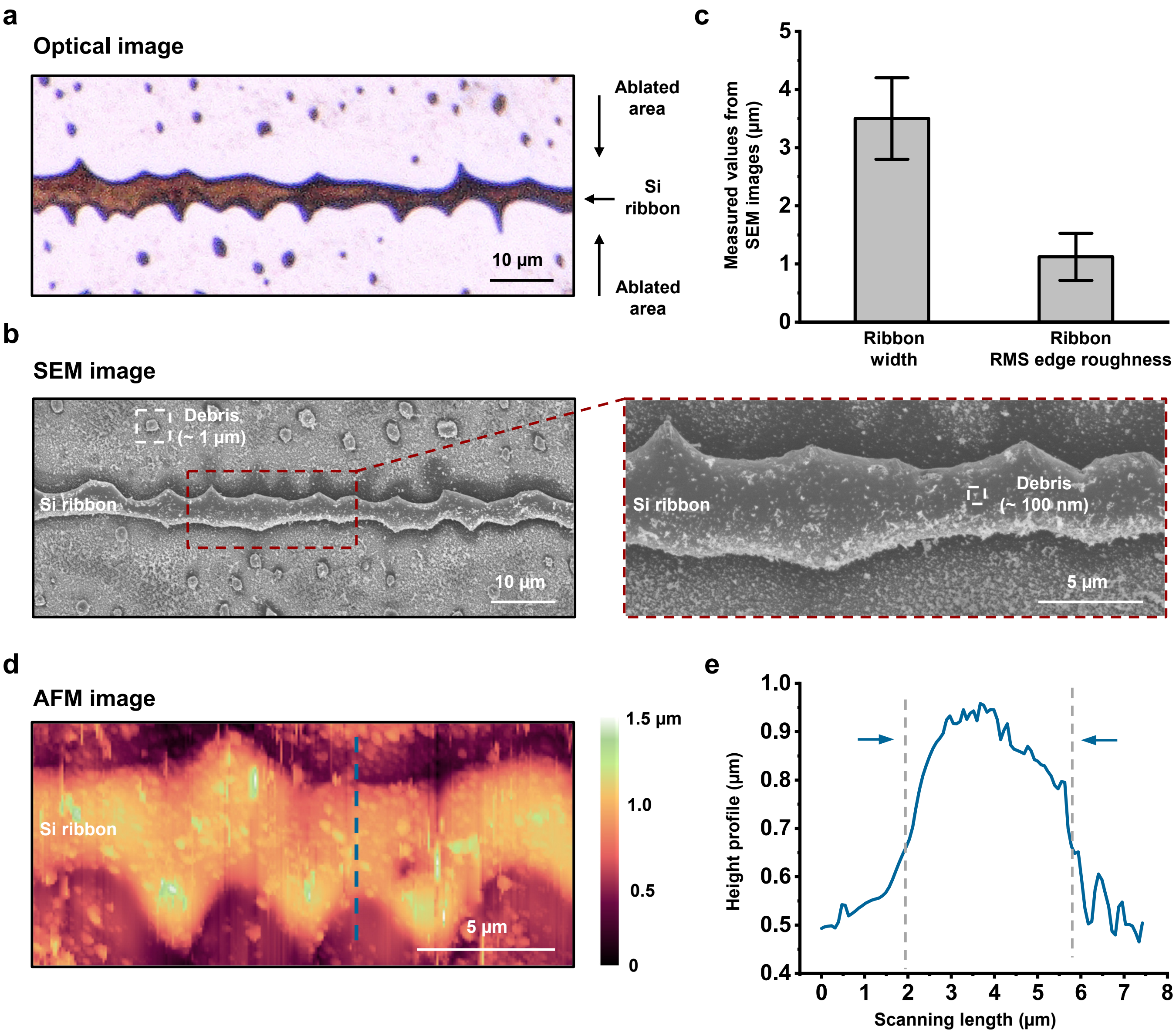


**Supplementary Figure 11 | Characteristic feature sizes of a monocrystalline Si ribbon patterned on a PLA substrate. a,** Optical image of the Si ribbon on a PLA substrate (Average power: 90 mW; scanning speed: 300 mm $s^{-2}$; frequency: 200 kHz; number of repetition: 1; grid distance: 1 μm; grid mode: Y-parallel; projected width: 8 μm). **b.** SEM characterization of the Si ribbon. Inset: high-magnification SEM image of the Si ribbon. **c,** SEM analysis indicates that the average ribbon width and the RMS edge roughness are 3.5 ± 0.7 and 1.1 ± 0.4 μm, respectively. **d,** AFM characterization of the Si ribbon. **e,** AFM analysis reveals that the full width at half maximum of the ribbon is ~ 4 μm.

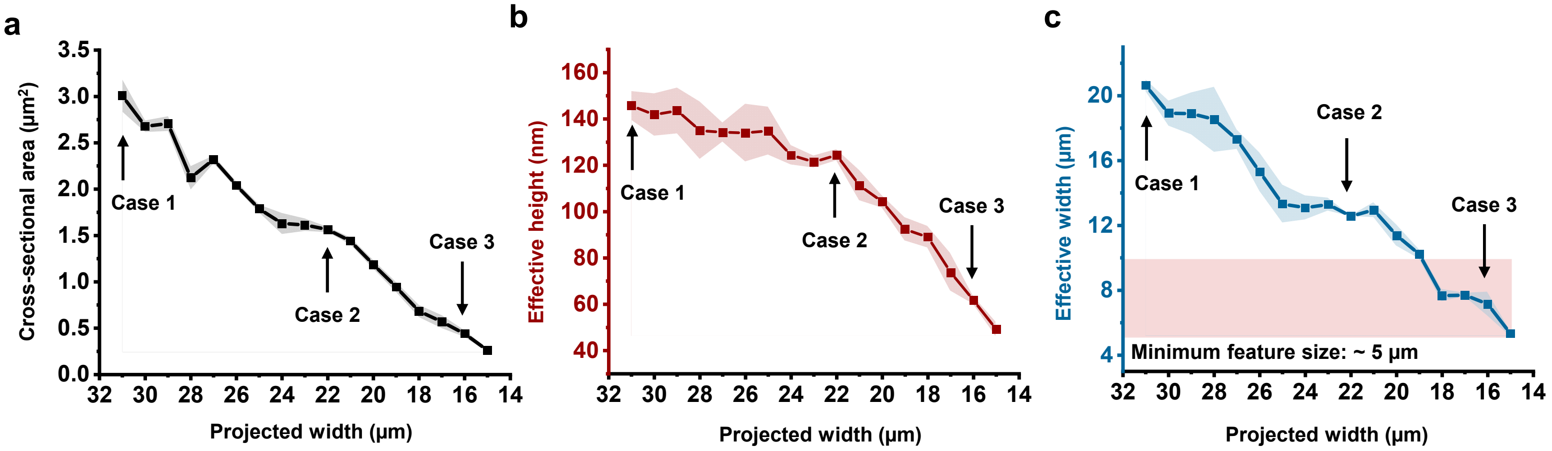


**Supplementary Figure 12 | Quantitative characterization of a trench shape formed by laser method. a-c,** Cross-sectional area (**a**), peak height (**b**), and effective width (**c**) as functions of projected width. The minimum feature size is ~ 5 μm. The shaded areas denote the standard deviation.

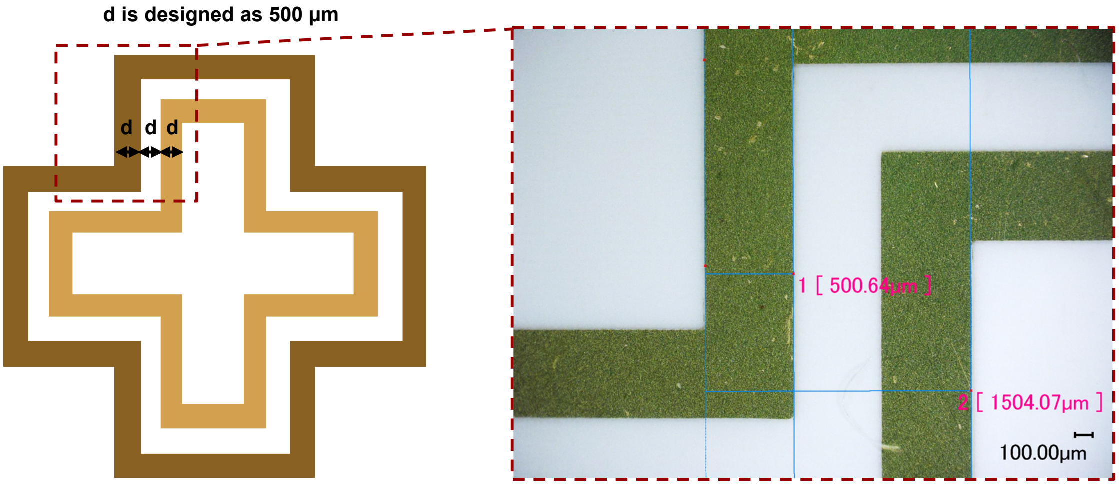


**Supplementary Figure 13 | Schematic illustration and optical micrograph of the pattern for characterizing overlay registration, corresponding to Fig. 2j.** The distance between two crisscross patterns is designed to 500 µm. The difference between the actual measured distance and the designed distance determines the overlay registration.

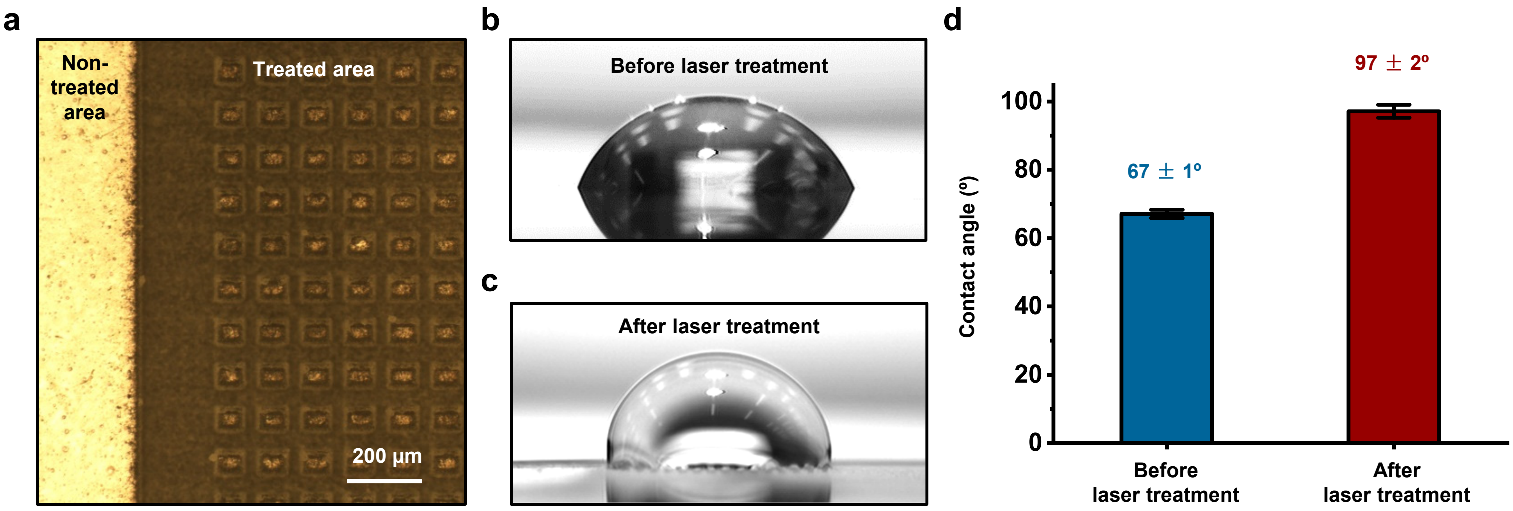


**Supplementary Figure 14 | Laser ablation method for surface treatment on substrates. a,** Laser-treated micro-textured PLA surface (right) compared with pristine PLA surface (left). **b-c,** Cross-sectional images of a water droplet (volume: 1.5 μL) on the pristine PLA surface (b) and laser-treated micro-textured PLA surface (c). **d,** Contact angle values of the pristine and laser-treated surfaces are 67 ± 1 ° and 97 ± 2°, respectively. The laser treatment enhances surface hydrophobicity.

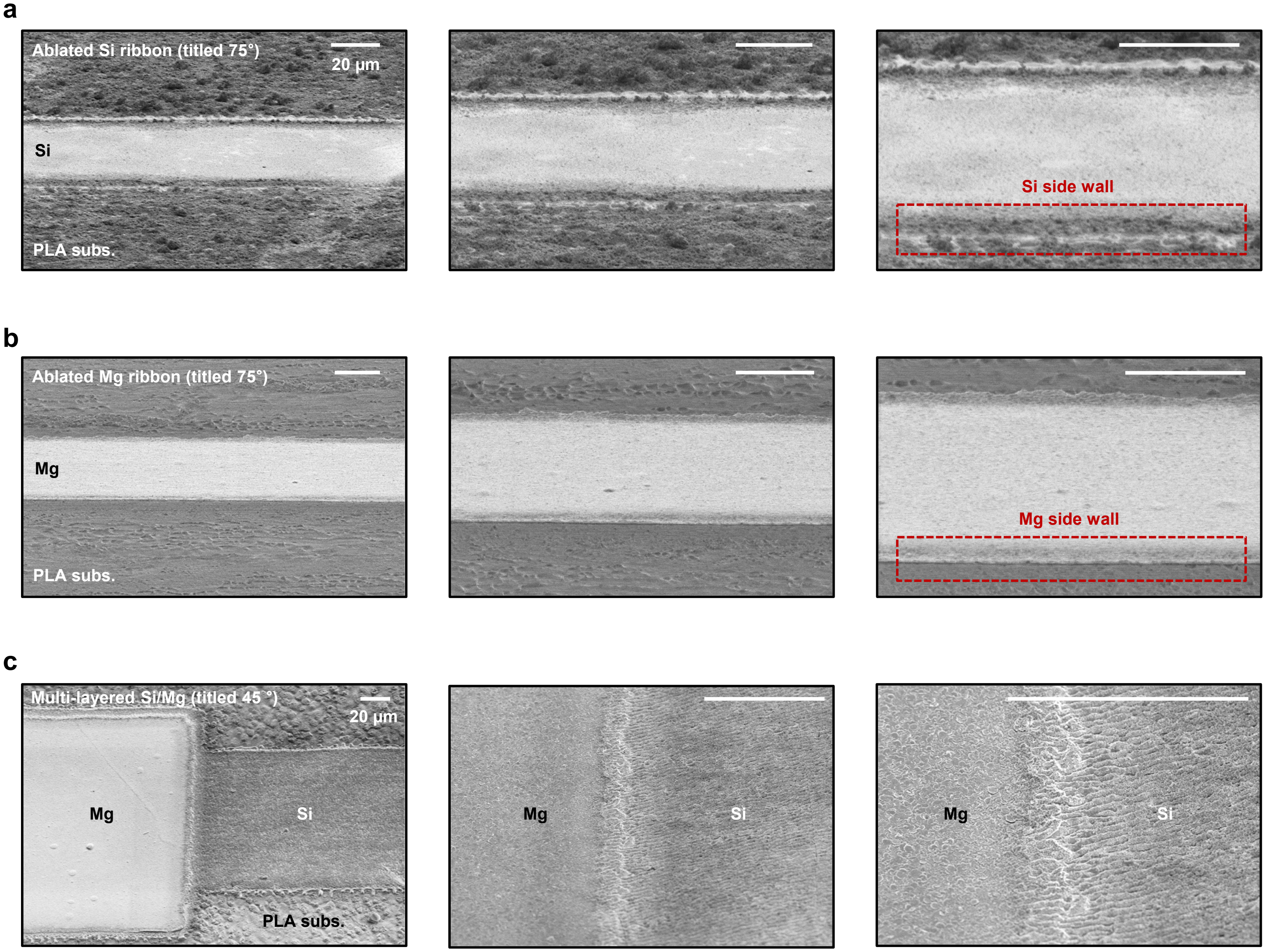


**Supplementary Figure 15 | SEM characterization of laser-ablated Si and Mg structures. a,** SEM images (tilted angle: 75°) of an ablated Si ribbon (width: 50 μm; thickness: 2 μm) on a PLA substrate (thickness: 50 μm). **b,** SEM images (tilted angle: 75°) of an ablated Mg ribbon (width: 50 μm; thickness: 500 nm) on a PLA substrate (thickness: 50 μm). **c,** SEM images (titled angle: 45°) of a bi-layer Mg/Si structure (thickness: 300 nm for top Mg and 2 μm for bottom Si) on a PLA substrate (thickness: 50 μm).

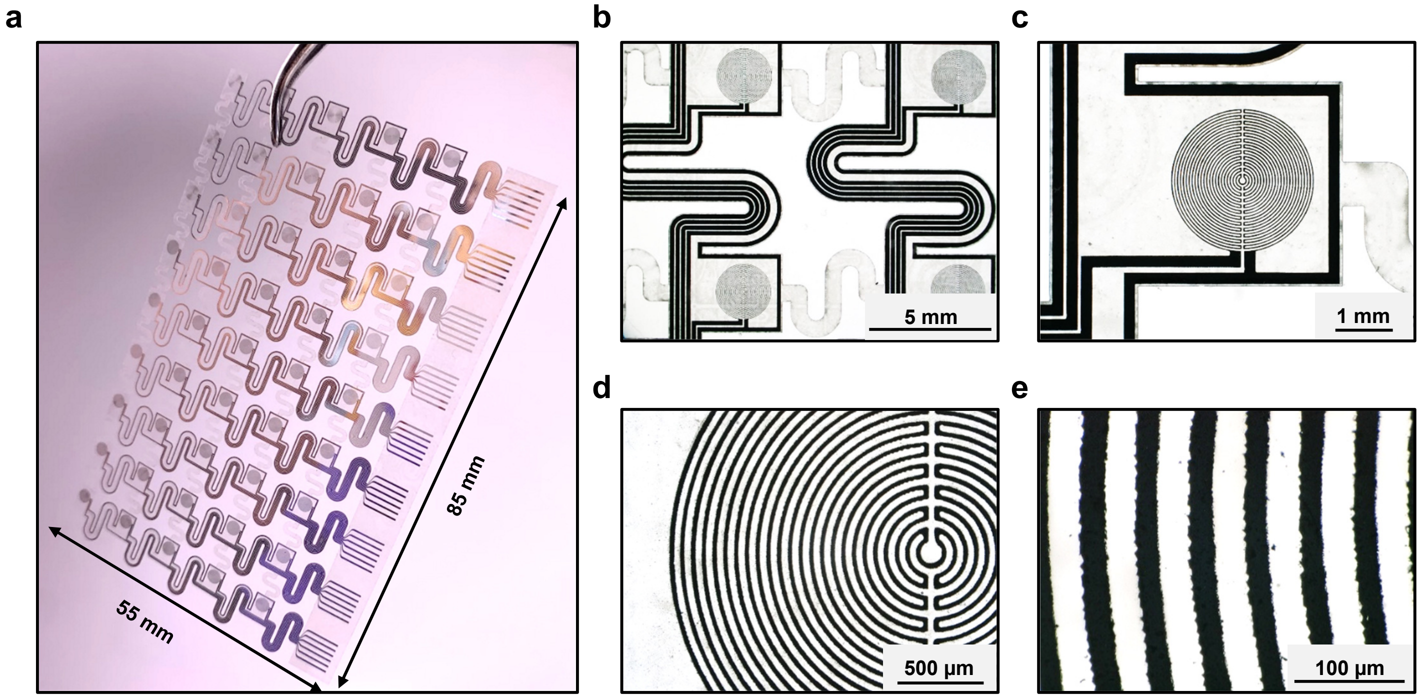


**Supplementary Figure 16 | Capability for forming arrays of devices over large areas.** Optical micrograph of an array (materials: Mg/PLA; thickness: 0.3/50 µm; dimension: 85 × 55 mm) of 45 thin-film resistive-type temperature sensors (arrangement: 9 × 5), on a serpentine-type substrate of PLA, to allow stretchability.

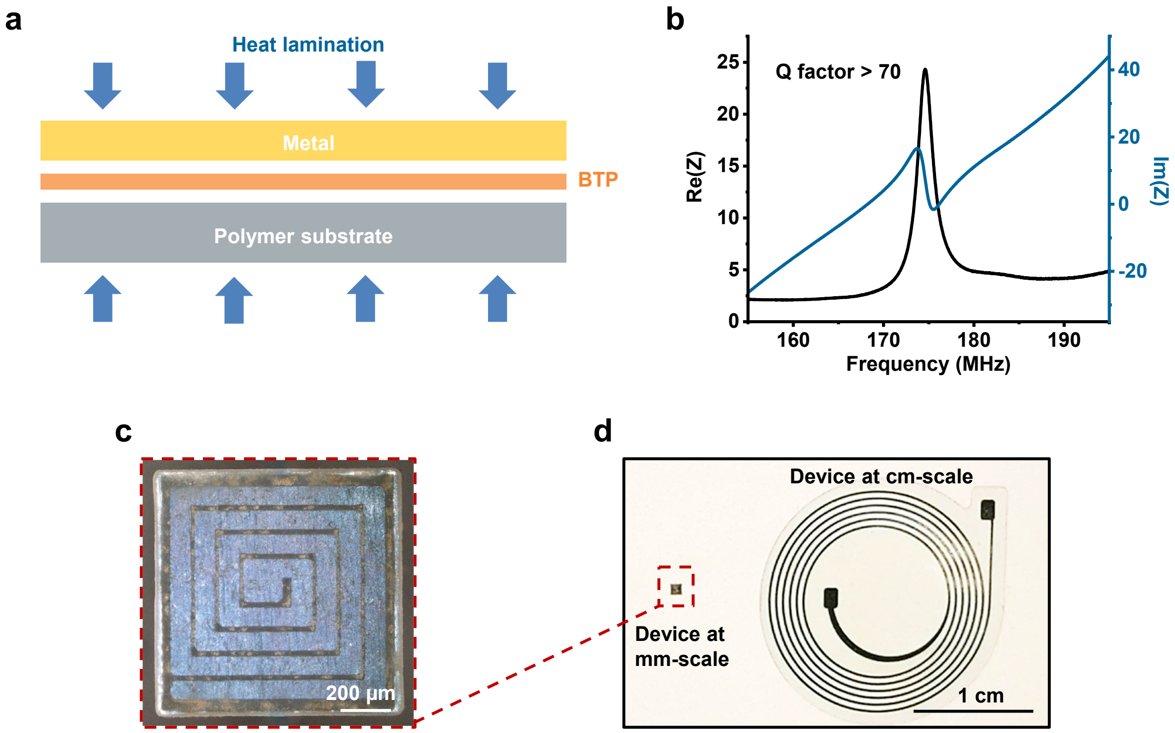


**Supplementary Figure 17 | Laser ablation strategy for bioresorbable inductors at the cm- and sub-mm scales. a,** BTP adhesive enables robust lamination of the metal layer on a polymer substrate by thermal compression. **b,** The Mg inductor presents a resonance frequency of ~ 175 MHz with a Q factor larger than 70. **c-d,** Sub-mm scale (diameter: ~ 1 mm; number of turns: 4; line width: 90 µm; spacing: 20 µm; zoom-in image in **c**) and cm-scale (zoom-in image in **d**) inductors fabricated by laser ablation.

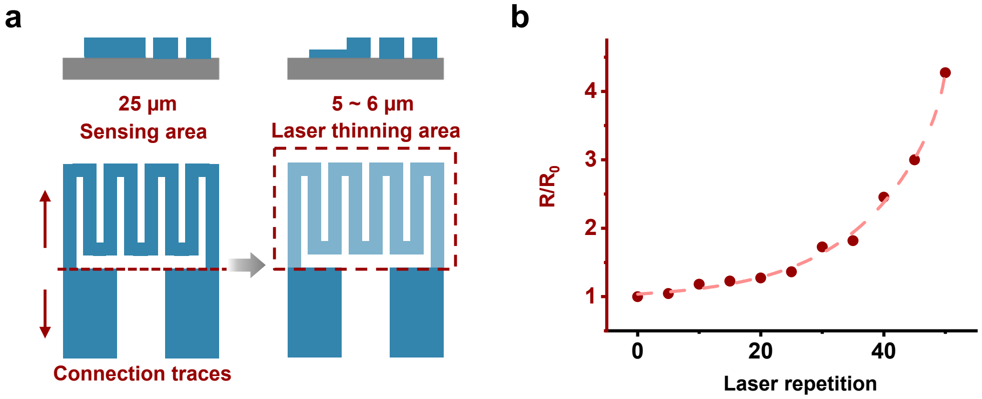


**Supplementary Figure 18 | Laser thinning strategy for bioresorbable resistive-type devices. a,** Laser thinning method reduces the thickness of the device sensing area from 25 to ~ 5 – 6 μm for high resistance and high sensitivity, while leaving the thicknesses of the connection traces unchanged (thickness: 25 μm). **b,** The resistance of the entire device increases by ~ 3.5 times when the thickness of the sensing area is reduced from 25 to ~ 5 – 6 μm. The resistance of the sensing area is inversely proportional to the thickness.

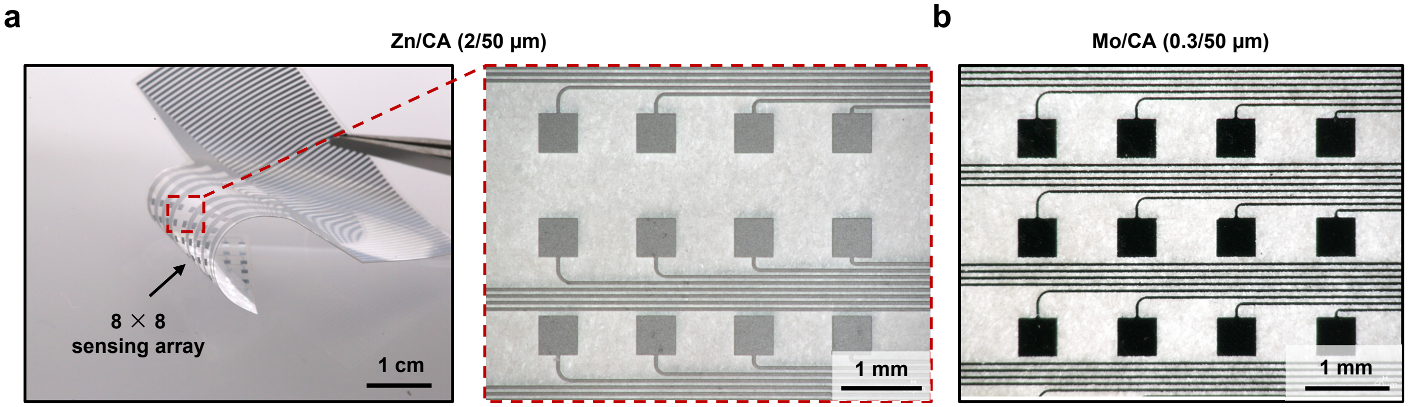


**Supplementary Figure 19 | Laser patterning of Zn (a) and Mo (b) on CA substrates into flexible sensing arrays.**

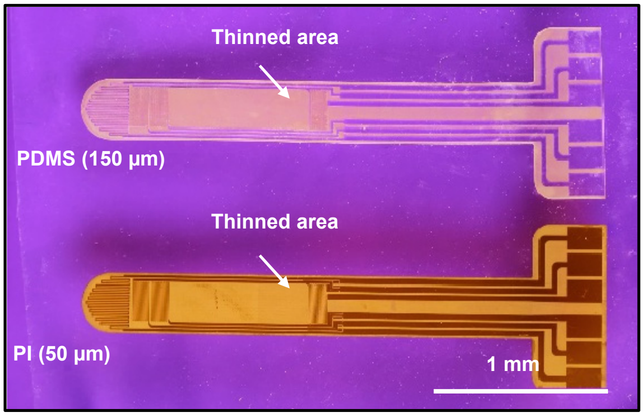


**Supplementary Figure 20 | Laser thinning and cutting of non-eco/bioresorbable polymers, including polyimide (PI) and polydimethylsiloxane (PDMS), into probe shapes.**

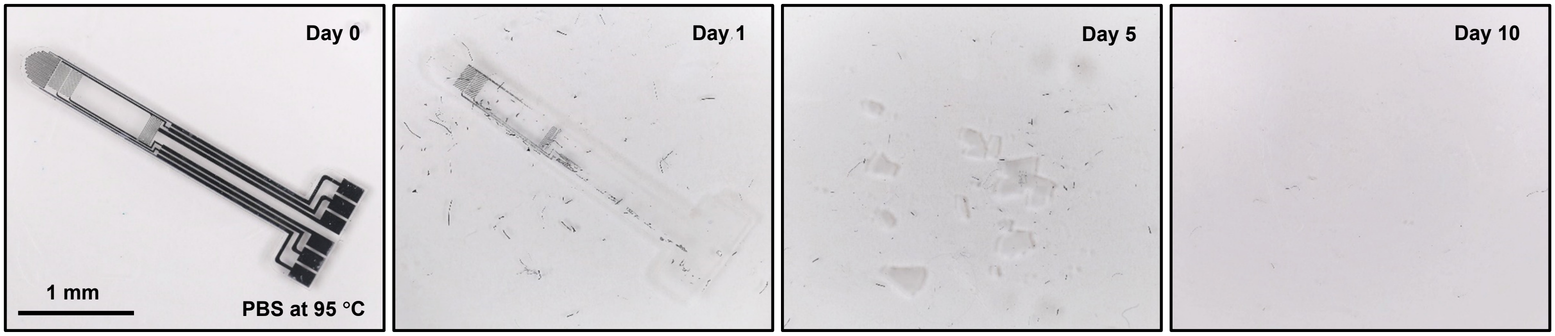


**Supplementary Figure 21 | Bioresorption of laser-fabricated devices.** Water-soluble electronic materials can be patterned by laser ablation into devices that harmlessly bioresorb in simulated biofluids, as illustrated here through images of a microvascular flow sensing probe at different times of immersion in PBS at 95 °C, pH 7.4 for accelerated testing.

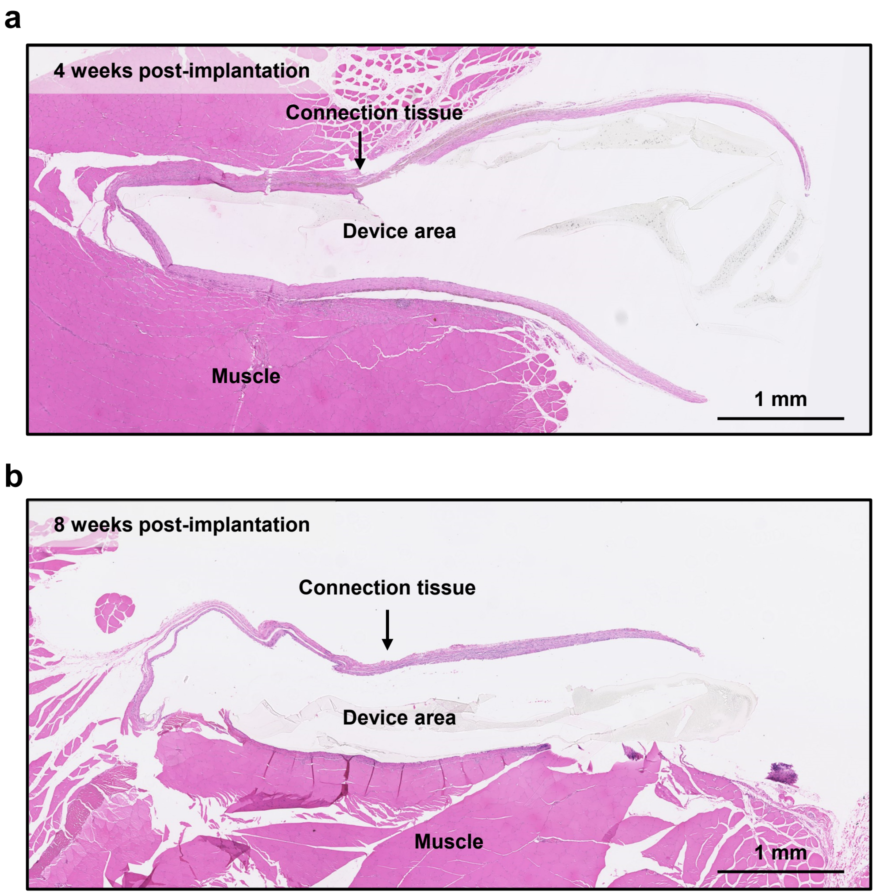


**Supplementary Figure 22 | Biocompatibility of bioresorbable sensing probes formed by laser ablation.** H&E staining of tissue from the leg of a rat model 4 weeks (**a**) and 8 weeks (**b**) after implantation.

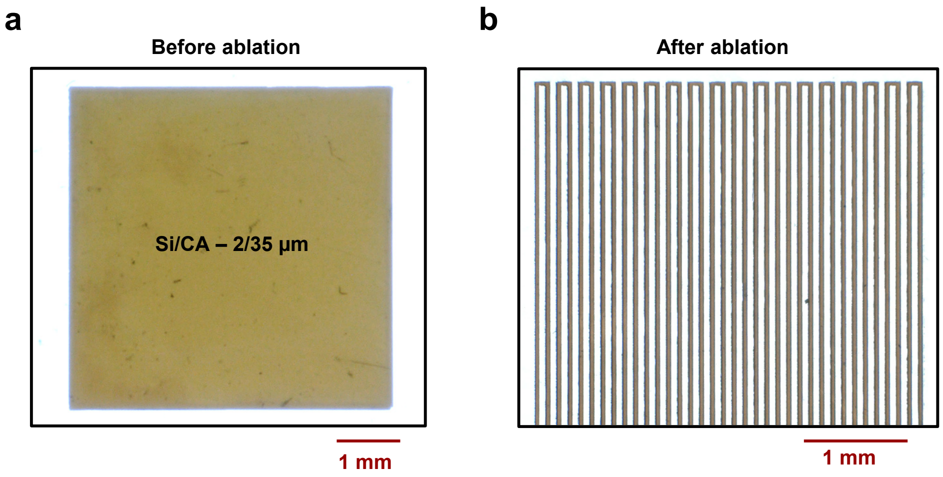


**Supplementary Figure 23 | Laser ablation strategy for Si-based resistive-type devices. a-b,** Optical micrographs of pristine (**a**) and ablated (**b**) monocrystalline Si (thickness: 2 μm) on a CA substrate (thickness: 35 μm).

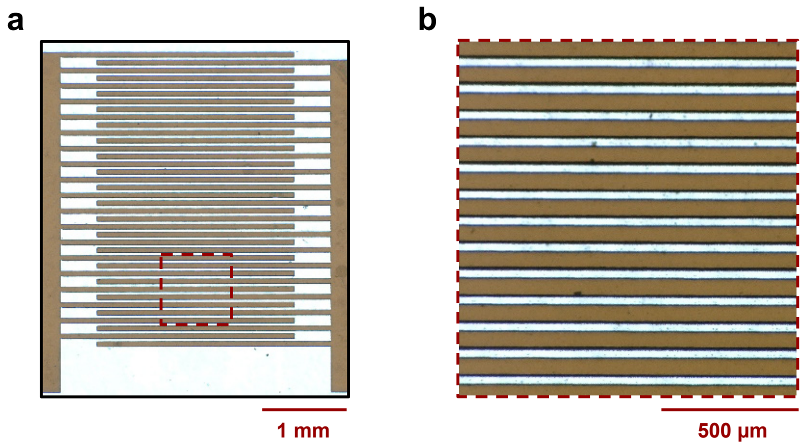


**Supplementary Figure 24 | Laser ablation strategy for forming Si-based capacitive-type devices. a-b,** Optical micrograph of the interdigitated structure (**a**), with a magnified image in **b** (line width: 100 μm).

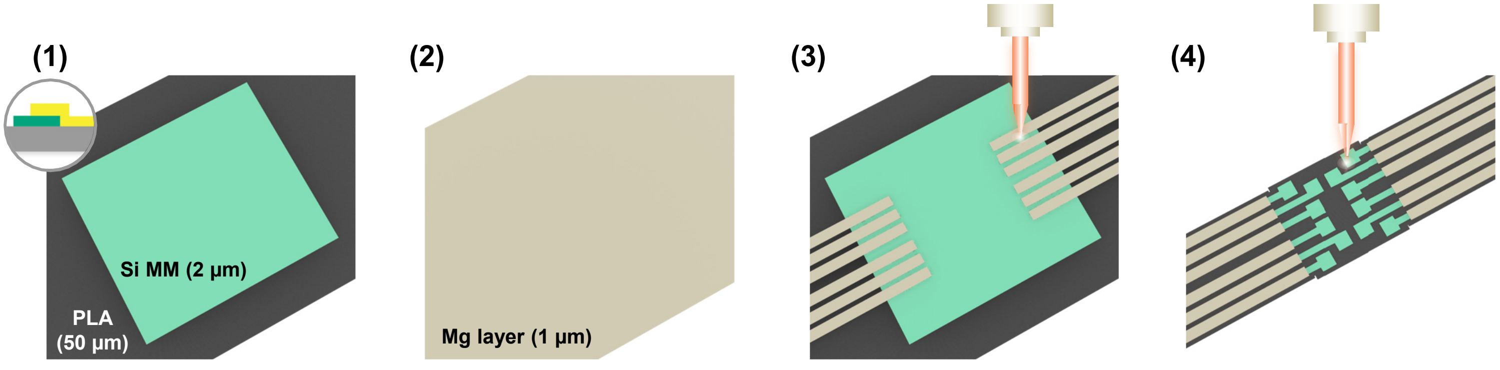


**Supplementary Figure 25 | Schematic illustration of an alternative process for forming multi-layer Si-based electrode arrays.** The process consists of (1) transfer printing of a monocrystalline highly n-doped Si MM (thickness: 2 µm) on a PLA substrate (thickness: 50 µm), (2) vacuum depositing a uniform layer of Mg (thickness: 1 µm), (3) ablating the Mg layer to define connection traces, and (4) ablating the Si MM to define an array of electrodes and to cut the PLA substrate into a ribbon shape.

Randles equivalent circuit fitting model

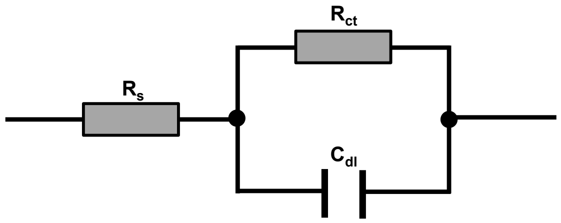


| Si electrode | $R_{ct}$ (MΩ) | Fitting error (%) | $C_{dl}$ (μF cm$^{-2}$) | Fitting error (%) |
|---|---|---|---|---|
| 1 | 225 | 32.54 | 2.94 | 2.58 |
| 2 | 205 | 31.99 | 2.70 | 3.12 |
| 3 | 243 | 32.01 | 2.28 | 3.10 |
| 4 | 290 | 39.46 | 3.46 | 2.05 |

$R_s$: solution resistance; $R_{ct}$: charge transfer resistance; $C_{dl}$: doble layer capacitance

**Supplementary Figure 26 | Equivalent circuit model used to fit the electrochemical impedance spectra of Si-based electrodes.** The interface charge transfer resistance, $R_{ct}$, and double layer capacitance per unit area, $C_{dl}$, are ~ 250 MΩ and 2.8 μF cm$^{-2}$, respectively.

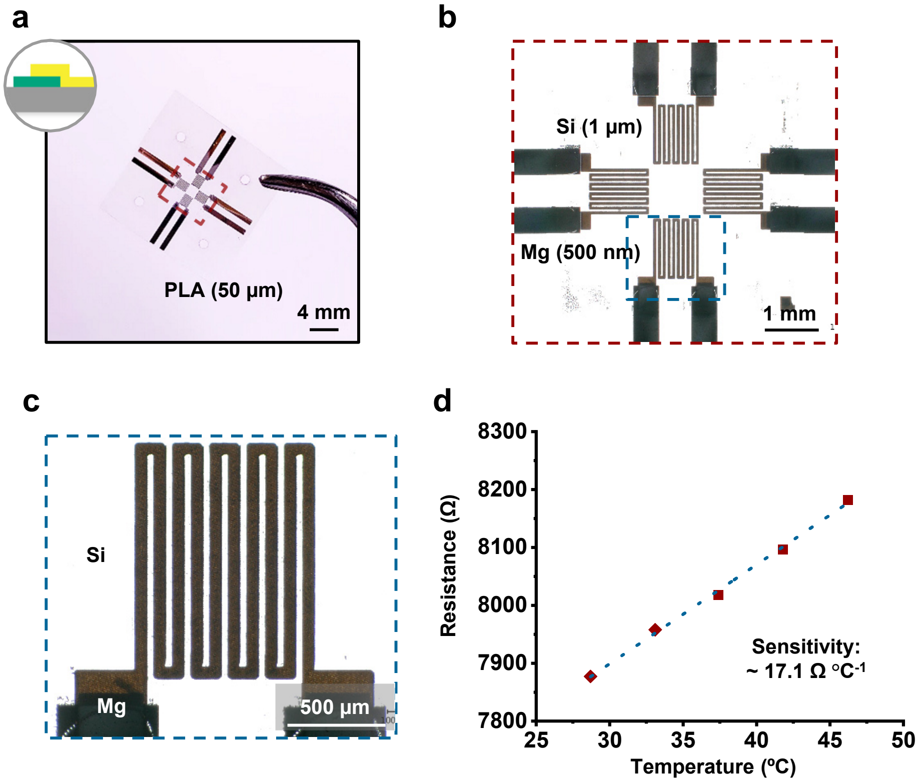


**Supplementary Figure 27 | Multi-layer laser ablation strategy for Si-based temperature sensing arrays. a-c,** Optical micrographs of the device, including four temperature sensors, with highly p-doped monocrystalline Si (thickness: 1 μm) as the sensing element and Mg (thickness: 500 nm) as interconnection traces. The laser ablation procedures are similar to those of the multi-layer Si-based electrode arrays. **d,** Resistance-temperature relation of such a device indicates a sensitivity of ~ 17.1 Ω $°C^{-1}$.

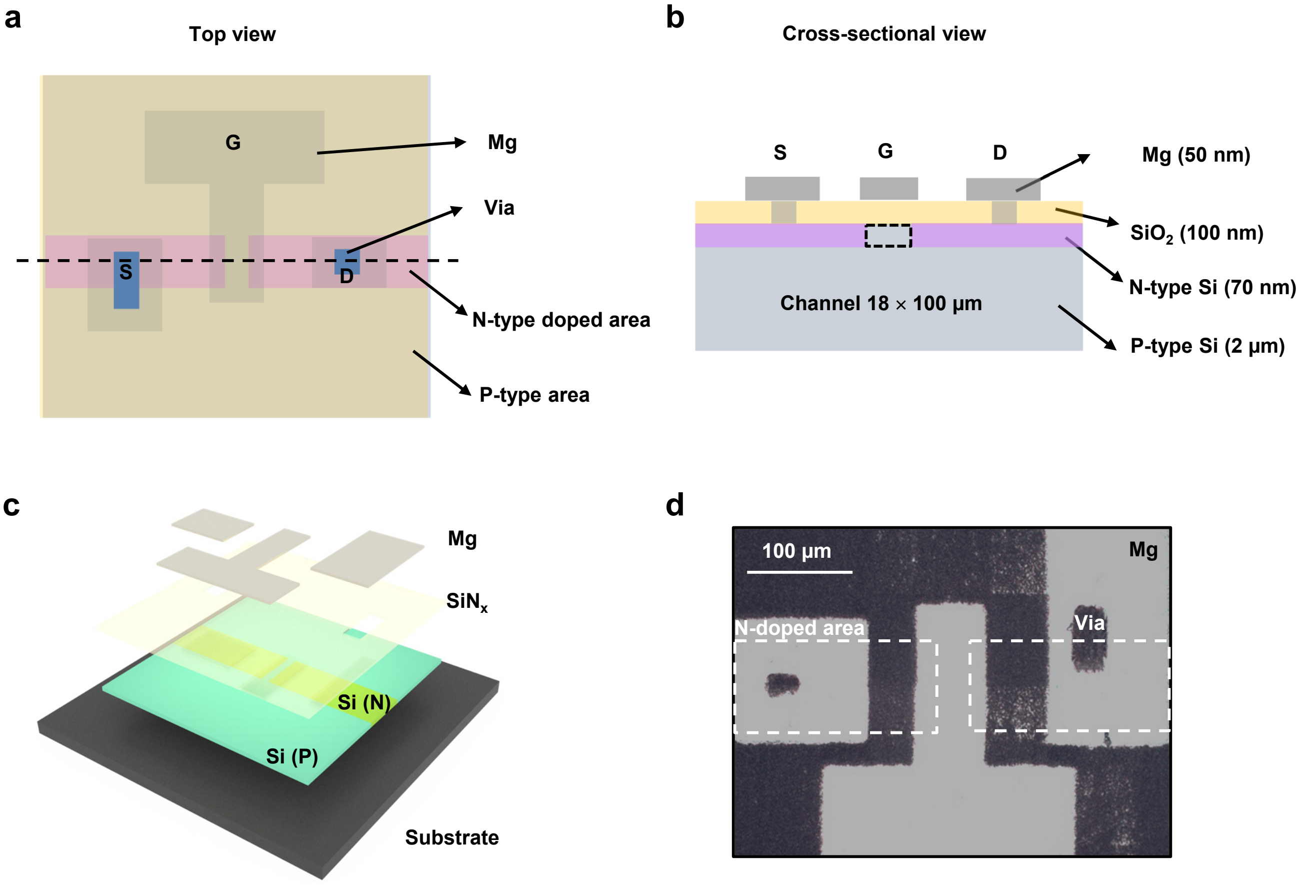


**Supplementary Figure 28 | Additional information for multi-layer Si-based n-channel MOSFETs fabricated by laser methods, corresponding to Fig. 5g-i, including top view (a), cross-sectional view (b), 3D exploded view (c), and optical micrograph (d) of the MOSFET devices.**

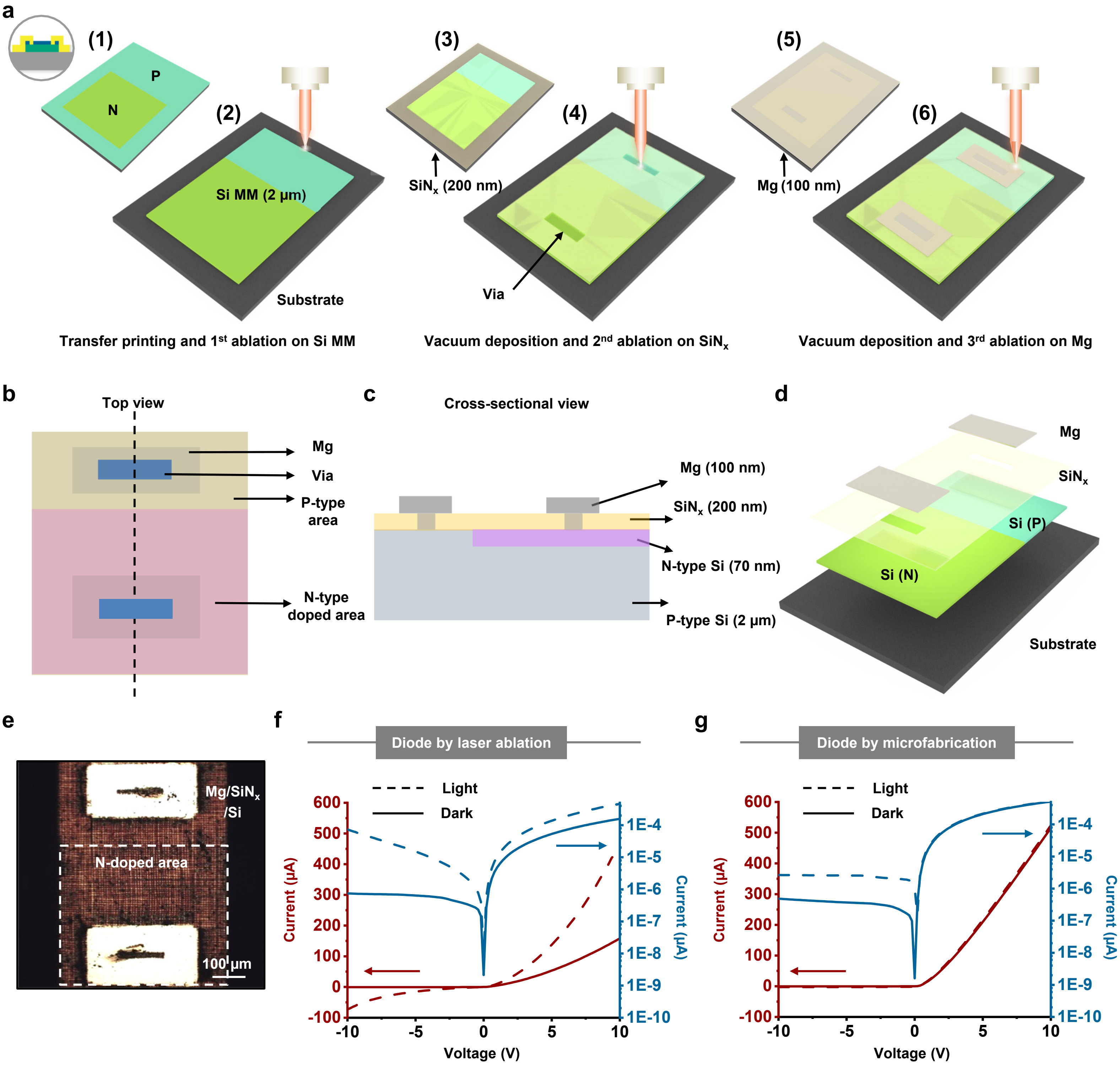
a
(1)
P
N
(2)
Si MM (2 μm)
Substrate
Transfer printing and 1st ablation on Si MM
(3)
SiNx (200 nm)
(4)
Via
Vacuum deposition and 2nd ablation on SiNx
(5)
Mg (100 nm)
(6)
Vacuum deposition and 3rd ablation on Mg
b
Top view
Mg
Via
P-type area
N-type doped area
c
Cross-sectional view
Mg (100 nm)
SiNx (200 nm)
N-type Si (70 nm)
P-type Si (2 μm)
d
Mg
SiNx
Si (P)
Si (N)
Substrate
e
Mg/SiNx /Si
N-doped area
100 μm
f
Diode by laser ablation
Light
Dark
Current (μA)
Current (μA)
Voltage (V)
g
Diode by microfabrication
Light
Dark
Current (μA)
Current (μA)
Voltage (V)

**Supplementary Figure 29 | Multi-layer laser ablation strategy for Si-based diodes. a,** Schematic illustration of the laser ablation process to form a multi-layer Si-based diode, consisting of (1) doping a specific region (dimension of highly n-doped area: 500 × 500 μm) on a monocrystalline Si MM (thickness: 2 μm), (2) ablation to pattern the Si MM into a rectangular pattern (dimension: 500 × 800 μm) into the PN junction pattern, (3) vacuum deposition of a uniform $SiN_x$ dielectric layer (thickness: 200 nm), (4) ablation of the $SiN_x$ layer to define shapes similar to those of Si and with two exposed vias (dimension: 50 × 140 μm), (5) vacuum deposition of a uniform Mg layer (thickness: 100 nm), and (6) ablation of the Mg layer to define two connection pads (dimension: 155 × 280 μm). **b-e,** top view (**b**), cross-sectional view (**c**), 3D exploded view (**d**), and optical micrograph (**e**) of the diode fabricated by laser ablation. **f,** Photoresponse characteristics. The intensity of the white LED used in the light environment is 10,000 lux.

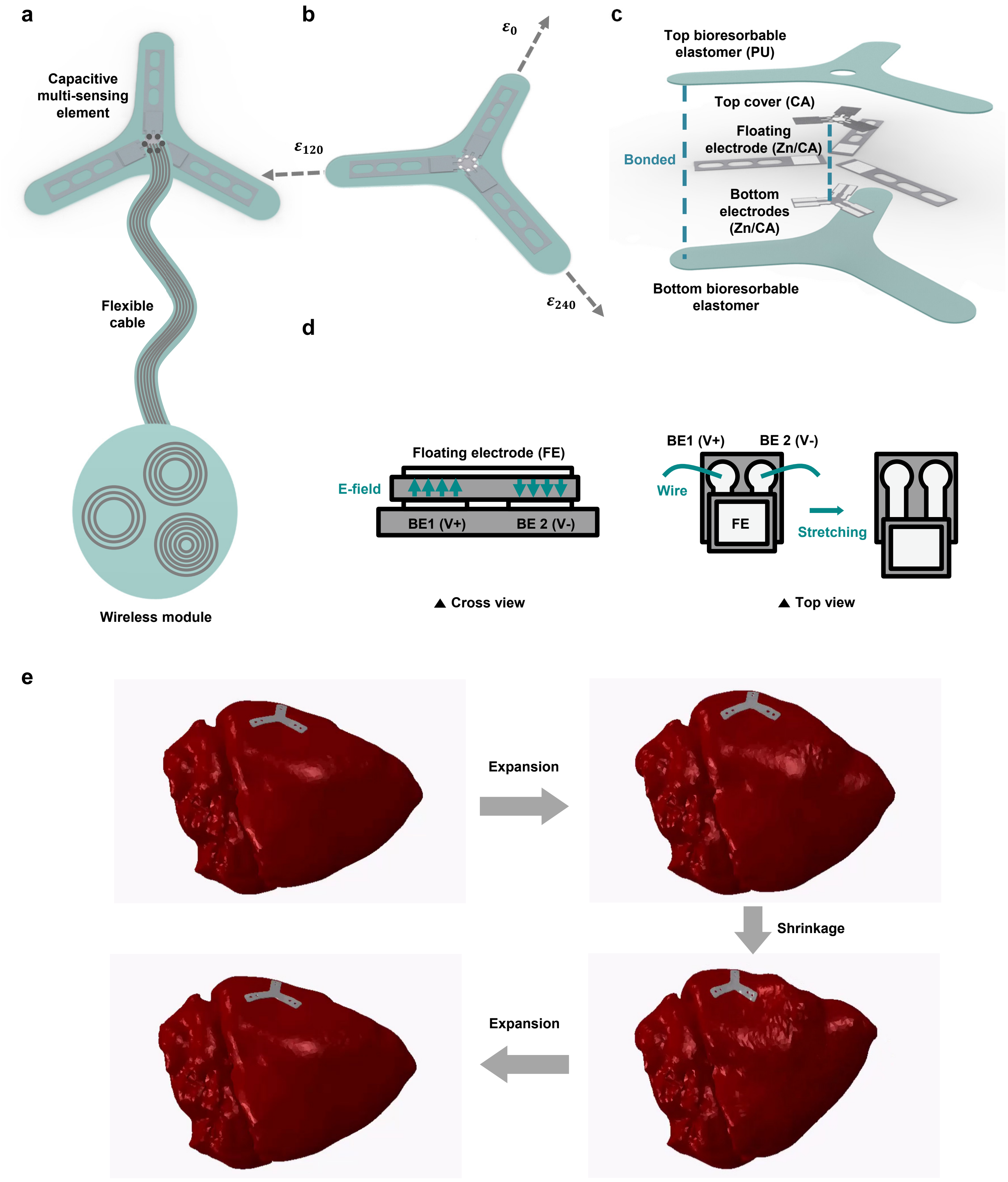
a
Capacitive multi-sensing element
Flexible cable
Wireless module
b
$\varepsilon_0$
$\varepsilon_{120}$
$\varepsilon_{240}$
c
Top bioresorbable elastomer (PU)
Top cover (CA)
Floating electrode (Zn/CA)
Bonded
Bottom electrodes (Zn/CA)
Bottom bioresorbable elastomer
d
Floating electrode (FE)
E-field
BE1 (V+)
BE 2 (V-)
▲ Cross view
BE1 (V+)
BE 2 (V-)
Wire
FE
Stretching
▲ Top view
e
Expansion
Shrinkage
Expansion

**Supplementary Figure 30 | Device architectures and working principles of the multi-sensing element. a,** Multi-sensing element connects to the wireless module by a long flexible cable. **b,** The element measures strains along the three arms. **c,** Exploded view of the element, including three functional layers (bottom electrodes, floating electrodes, and top cover) and two encapsulation layers. **d,** Working principles for strain sensing. The strain along each arm leads to a change in the relative position between the floating electrode and the pair of bottom electrodes, and therefore, a change in the capacitance. The change in capacitance results in a shift in resonance frequency. **e,** Expansion and contraction of the heart during each cardiac cycle leads to epicardial strains that can be measured by the multi-sensing element.

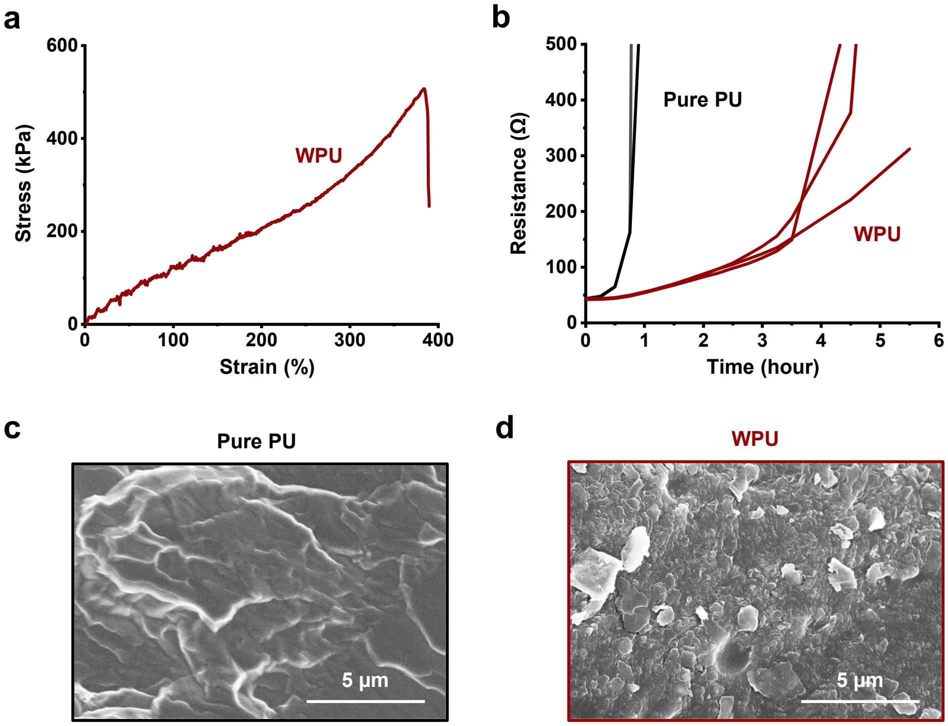


**Supplementary Figure 31 | Tissue-like mechanical and water barrier encapsulation properties of the WPU layer. a,** Stress-strain curve for the WPU indicates a tissue-like Young's modulus of ~ 100 kPa. **b,** Relative water permeation properties of WPU and pure PU, determined by the time dependence of the resistance of a thin-film Mg trace. Water that penetrates through the encapsulation layers dissolves the Mg traces, and therefore, increases the resistance. WPU has superior water barrier characteristics compared to those of pure PU. **c-d,** Scanning electron microscope (SEM) images of the cross-section of pure PU **(c)** and WPU **(d)**.

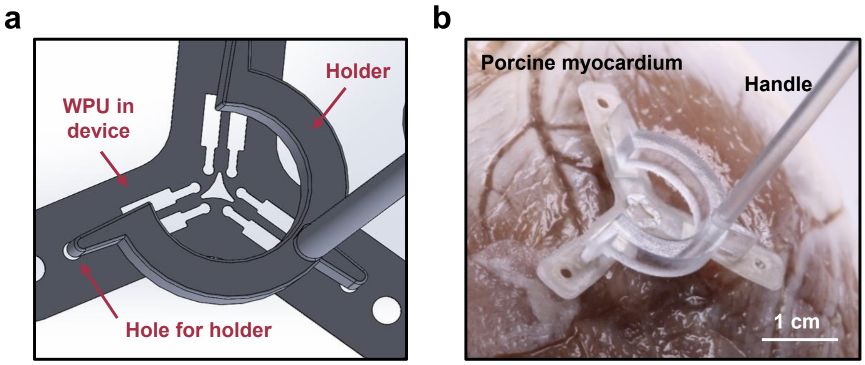


**Supplementary Figure 32 | Customized 3D-printed accessory to hold the device in a manner that avoids bending and fracture during the surgery. a,** Schematic illustration of the accessory**. b,** Optical image of the accessory to hold the device on the cardiac surface.

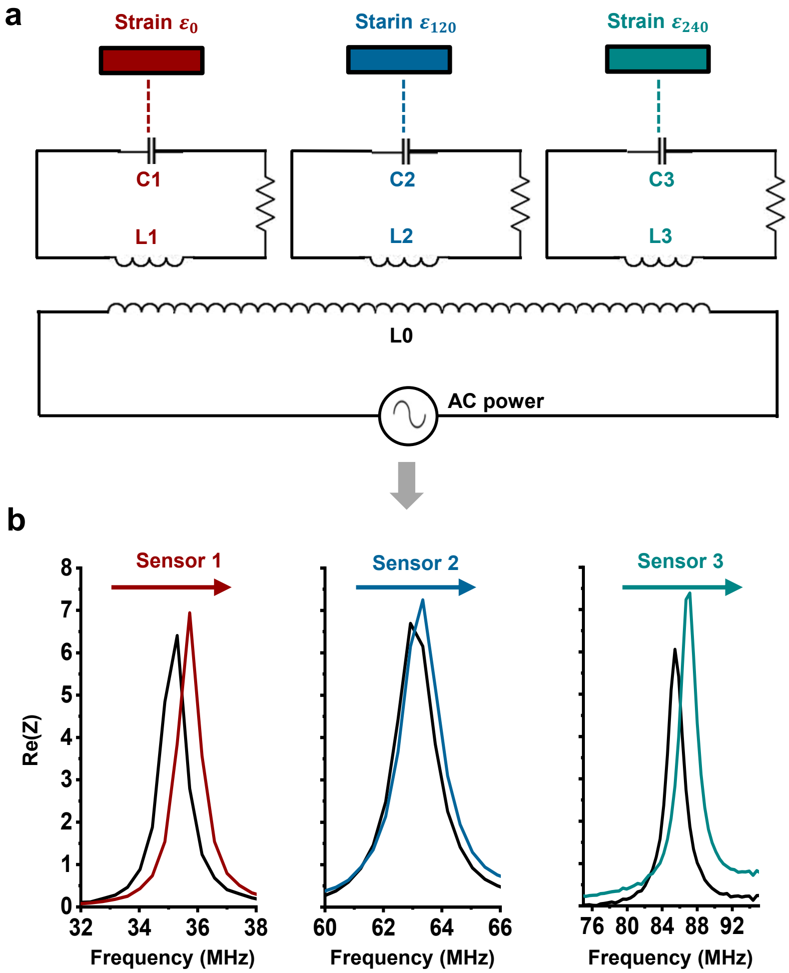


**Supplementary Figure 33 | Equivalent circuit model for simultaneous wireless measurements. a,** Equivalent circuit of the three LC-resonance sensing units in a multi-sensing element (top) and the wireless readout system (bottom). **b,** Stretching the arms leads to a reduction of the capacitance and an increase in the resonance frequency.

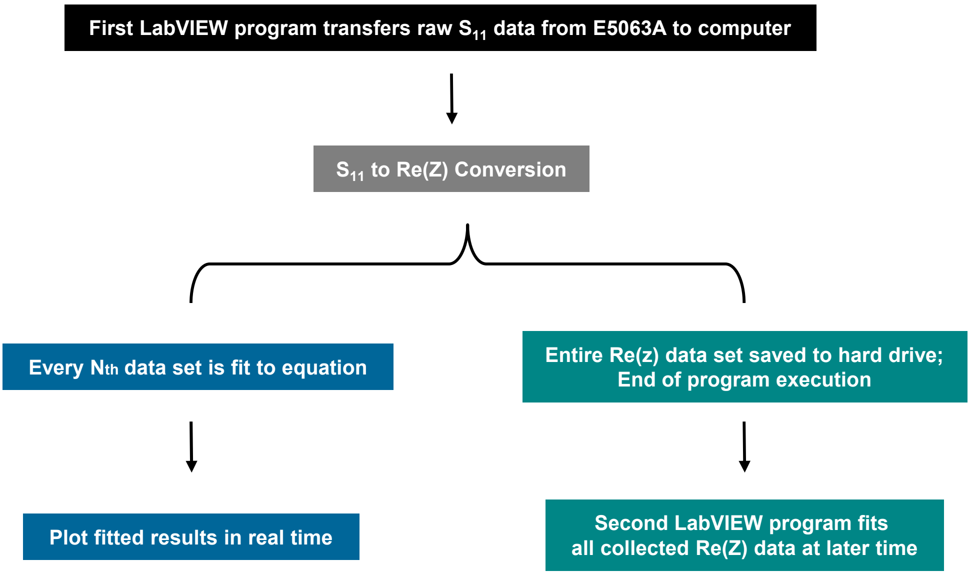


**Supplementary Figure 34 | Flow chart for a custom LabVIEW program for real-time data collection and analysis and subsequent further analysis.**

**a**

**Data collection and data fitting in real-time**

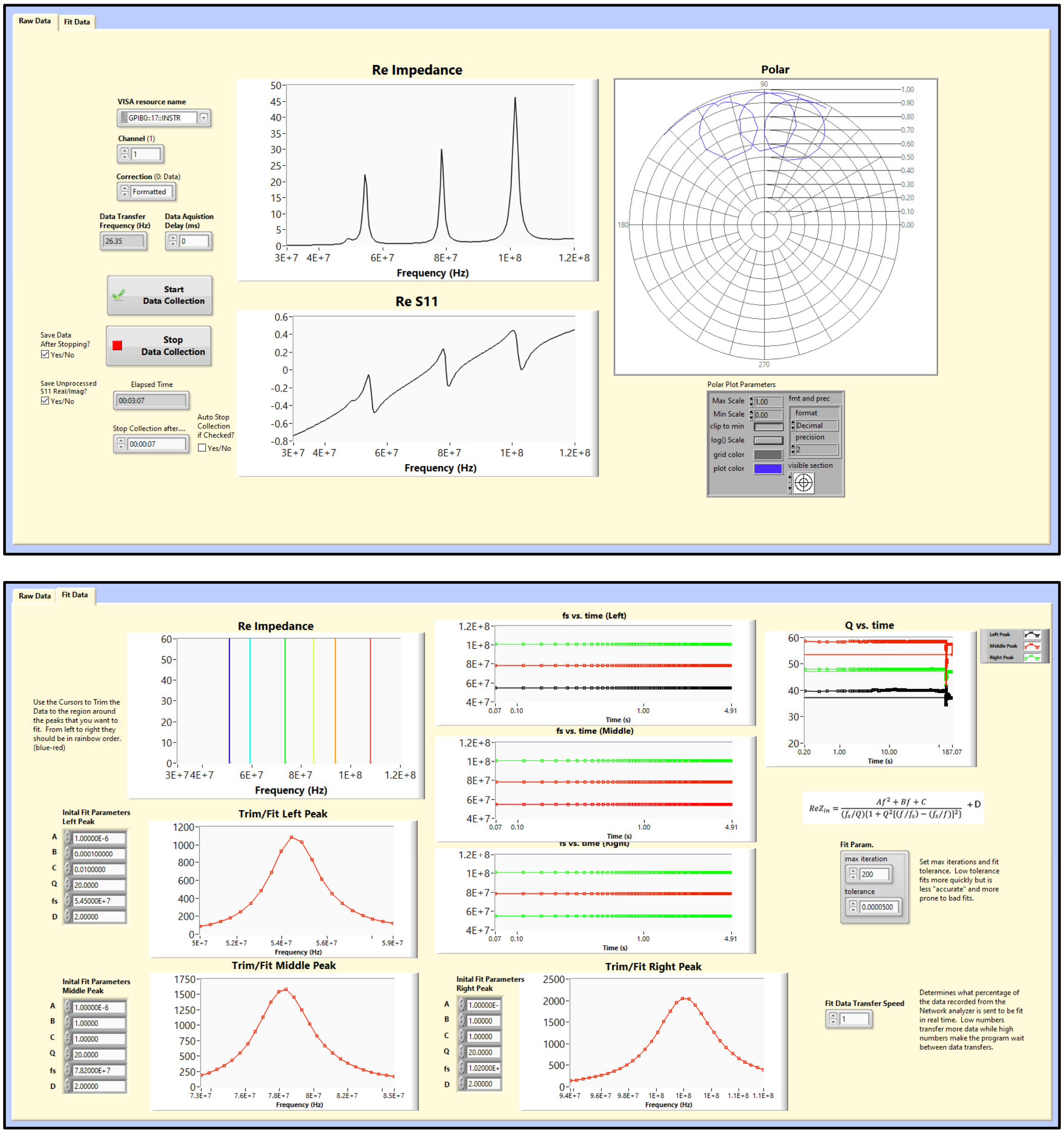


**b**

**Data fitting in post-processing**

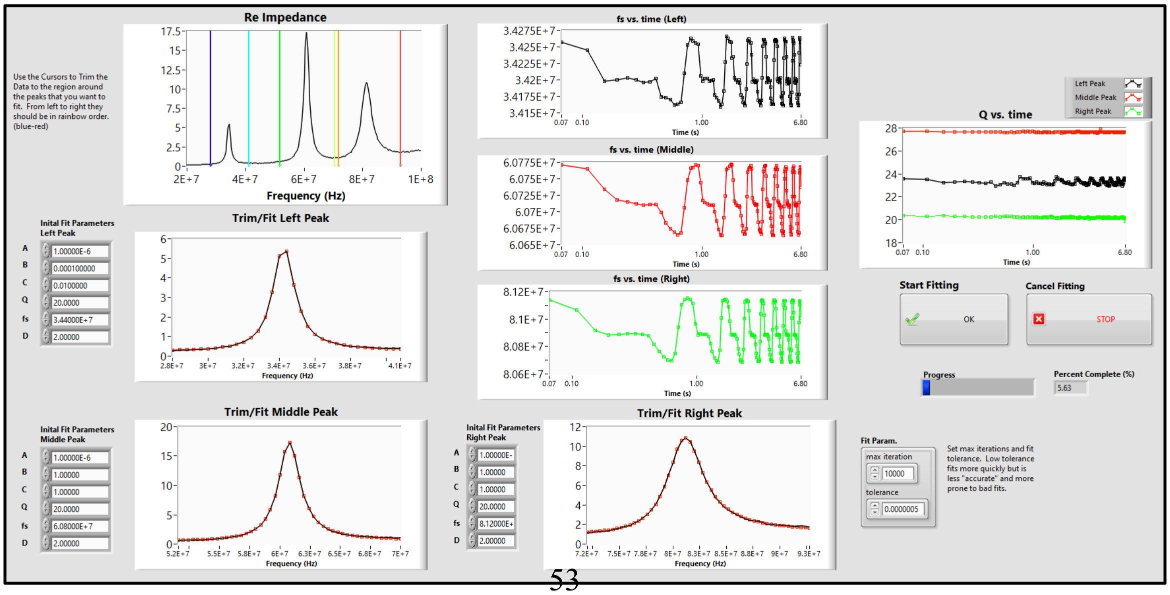

**Supplementary Figure 35 | Custom LabVIEW program for real-time data collection and analysis and subsequent further analysis. a,** The program collects and analyzes the reflection coefficient ($S_{11}$) data measured with the ENA network analyzer in real time and converts the $S_{11}$ data to the real part of the impedance (*Re(Z)*) accordingly, with further calculations on the resonance frequency ($f_s$) and Q factor ($Q$) in real time, albeit with low time resolution (maximum data frequency: ~ 5 Hz; lower than the data collection frequency (~ 25 Hz)). **b,** The program also post-processes all the data.

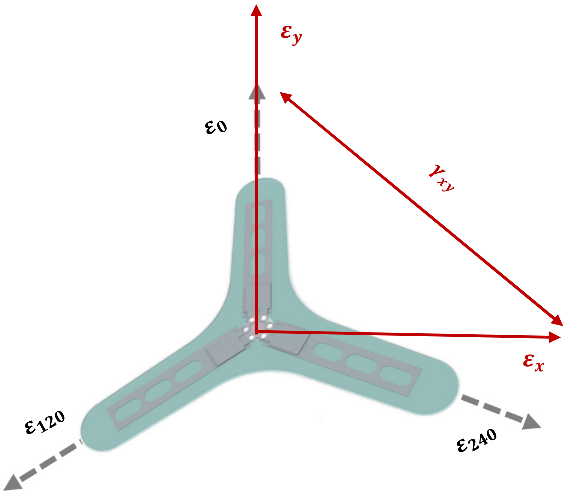


$$\varepsilon_x = \frac{2(\varepsilon_0 + \varepsilon_{240}) - \varepsilon_{120}}{3}$$

$$\varepsilon_y = \varepsilon_{120}$$

$$\gamma_{xy} = \frac{2}{\sqrt{3}}(\varepsilon_0 - \varepsilon_{240})$$

**Supplementary Figure 36 | Coordinate transformation to convert the strains along 0, 120, and 240° for each arm to principal strains ($\varepsilon_x$ and $\varepsilon_y$) and shear strain ($\gamma_{xy}$).**

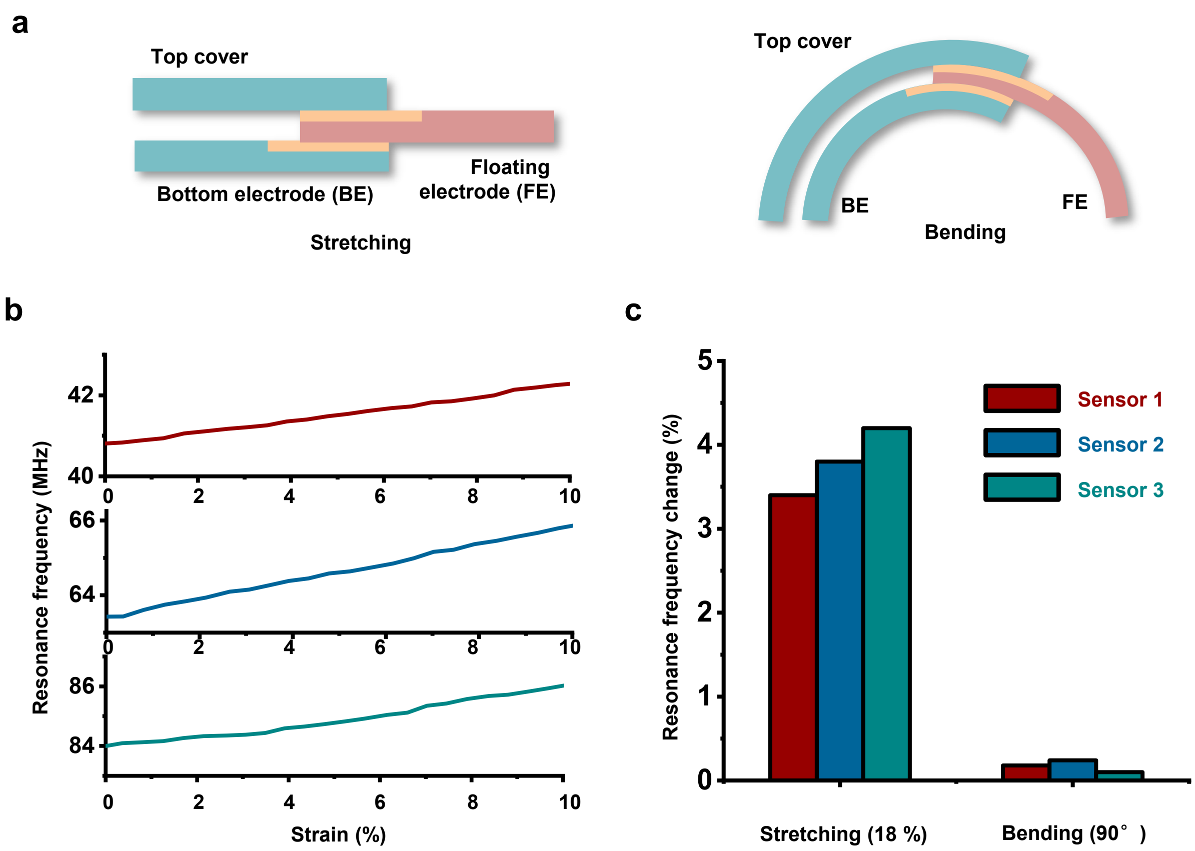


**Supplementary Figure 37 | Strain sensitivity and bending insensitivity of the multi-sensing element. a,** Functionality of the top cover. The top cover fixes the distance between the floating electrode and the pair of bottom electrodes; and therefore, the capacitance remains stable under curvature. **b,** Change in resonance frequency for each arm is ~ 1.5 – 2 % with a strain of 10 %, suggesting a sensitivity of ~ 200 kHz per unit strain. **c,** Change in resonance frequency is ~ 3 – 4 % with a strain of ~ 18 % and < 0.2 % under a bending angle of 90°, respectively, suggesting negligible sensitivity to bending.

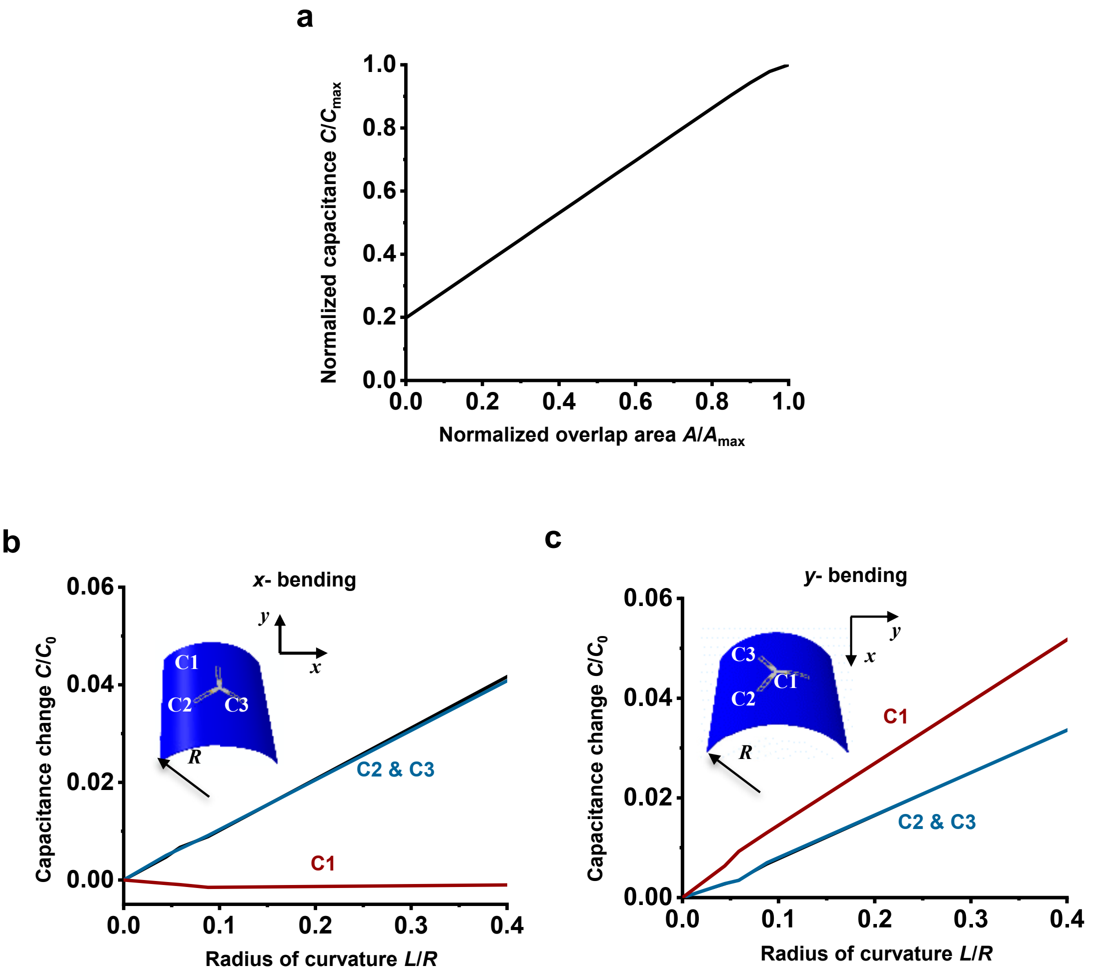


**Supplementary Figure 38 | Simulation results for the sensitivity to strain and bending.** **a,** Normalized capacitance exhibits a linear relationship with normalized overlap area/length (with fixed width) during stretching. **b-c,** Relationships between the capacitance change and the radius of curvature normalized by the device in-plane size $L$ for the three capacitors as a result of x- and y-bending. The capacitance change under curvature is negligible compared with that under stretching.

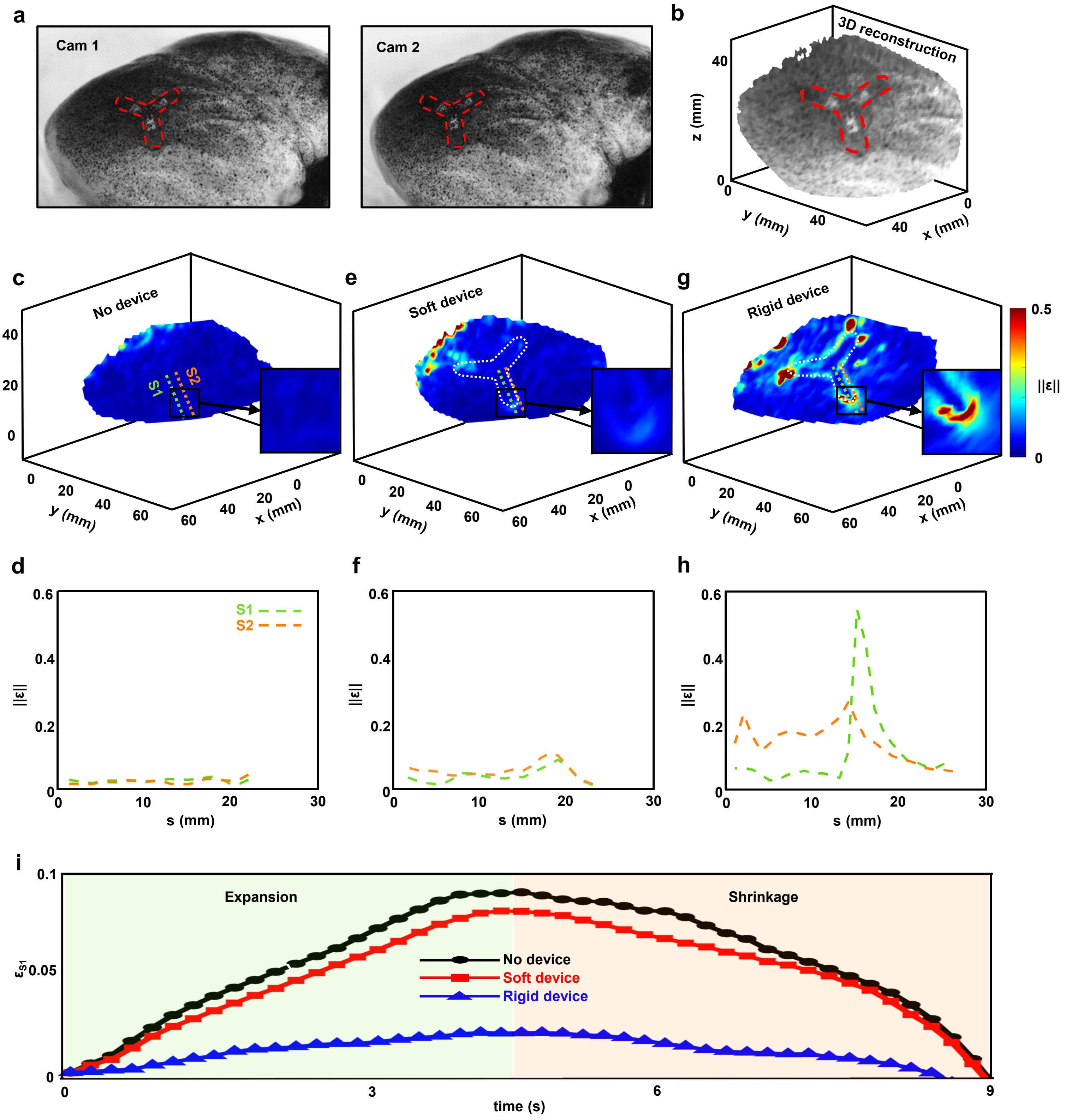
a
Cam 1
Cam 2
b
3D reconstruction
z (mm)
y (mm)
x (mm)
c
No device
S1
S2
e
Soft device
g
Rigid device
||ε||
d
f
h
s (mm)
i
Expansion
Shrinkage
No device
Soft device
Rigid device
$\varepsilon_{S1}$
time (s)

**Supplementary Figure 39 | Insignificant mechanical constraints on natural motions of the cardiac tissues. a,** Optical images from the two cameras for 3D-PTV. The porcine heart surface has many plastic circular black particles (diameter: 1 mm) for 3D-PTV. **b,** 3D reconstruction of the cardiac tissue with device mounted. **c-g,** Strain distributions of the cardiac surface without the device, with the device, and with a rigid film in a similar structure (Young's modulus: ~ 1 GPa) at maximum heart volume. **d-h,** Quantitative assessments of the strain values (S1 and S2) along two lines in **c-g** at full heart volume. In the cases without and with the device, the strain values are all lower than 0.1. By contrast, the strain values are much higher (maximum value over 0.5) for the case of a uniform film. **i,** Strain values (S1) during the entire expansion-contraction process. The deformations with the device (strain at the full volume: ~ 8 %) are similar to those without the device (strain at the full volume: ~ 9 %). By contrast, the attachment of a uniform film reduces the strain at full expansion to ~ 2 %.

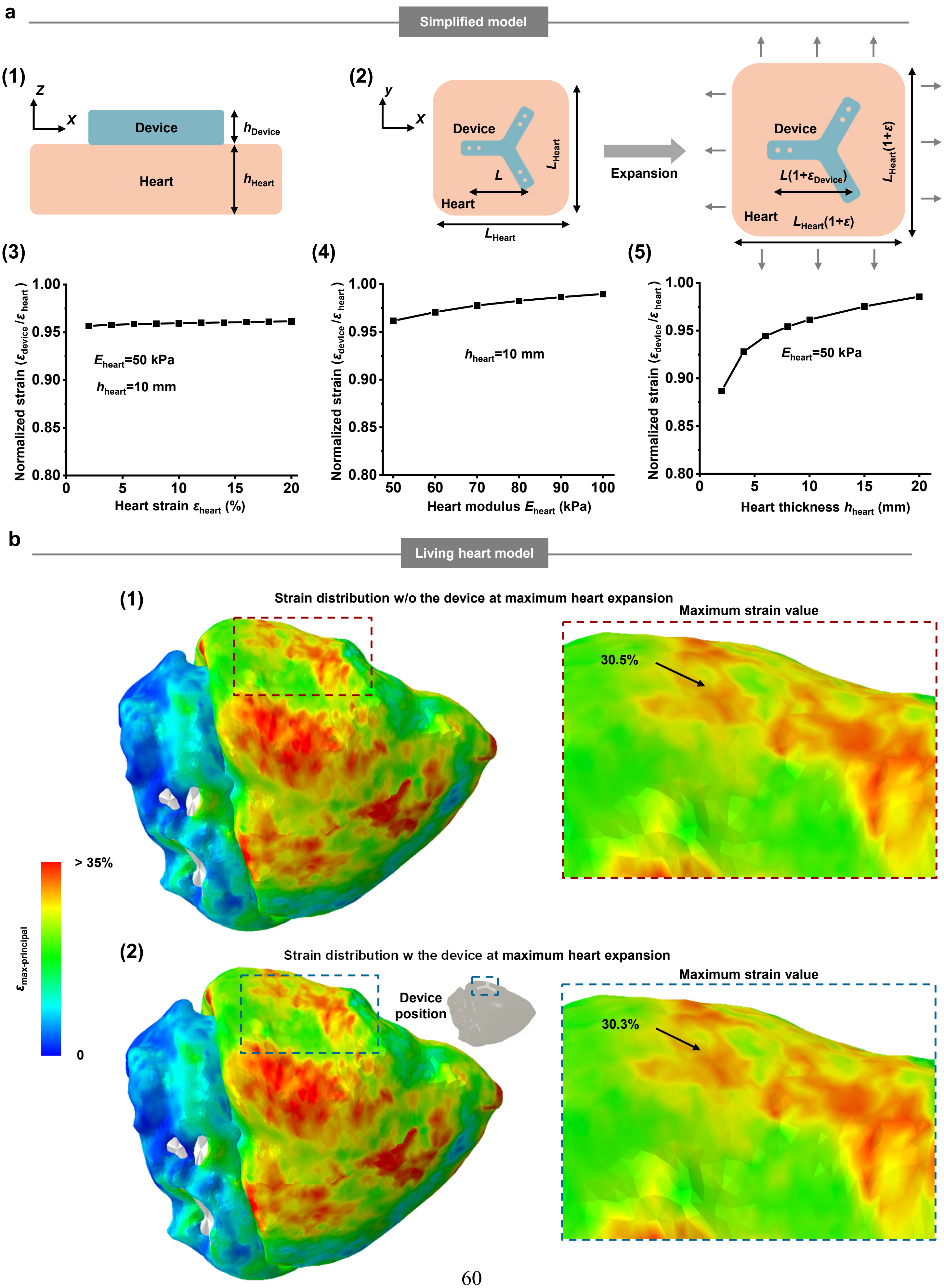
a
Simplified model
(1)
Device
Heart
h_Device
h_Heart
(2)
Device
Heart
L
L_Heart
Expansion
L(1+ε_Device)
L_Heart(1+ε)
(3)
E_heart=50 kPa
h_heart=10 mm
Normalized strain (ε_device/ε_heart)
Heart strain ε_heart (%)
(4)
h_heart=10 mm
Heart modulus E_heart (kPa)
(5)
E_heart=50 kPa
Heart thickness h_heart (mm)
b
Living heart model
(1)
Strain distribution w/o the device at maximum heart expansion
Maximum strain value
30.5%
> 35%
0
ε_max-principal
(2)
Strain distribution w the device at maximum heart expansion
Device position
Maximum strain value
30.3%

**Supplementary Figure 40 | Results using a simplified model and a living heart model on the constraints to natural motions of cardiac tissues. a,** Simplified model results. (1-2), Cross-sectional and top view schematic illustrations of the device on the cardiac tissues. (3-5) Normalized strain ($\varepsilon_{device}/\varepsilon_{heart}$; device strain, $\varepsilon_{device}$, divided by the heart strain, $\varepsilon_{heart}$) as functions of heart strain (3), heart modulus (4), and heart thickness (5). These results suggest a negligible influence of the device on motions of the heart. b, Living heart model results. Strain distribution without (1) and with (2) the device at maximum heart expansion. Insets: maximum strain values at maximum heart expansion without (1) and with (2) the device are 30.5 % and 30.3 %, respectively, suggesting negligible difference.

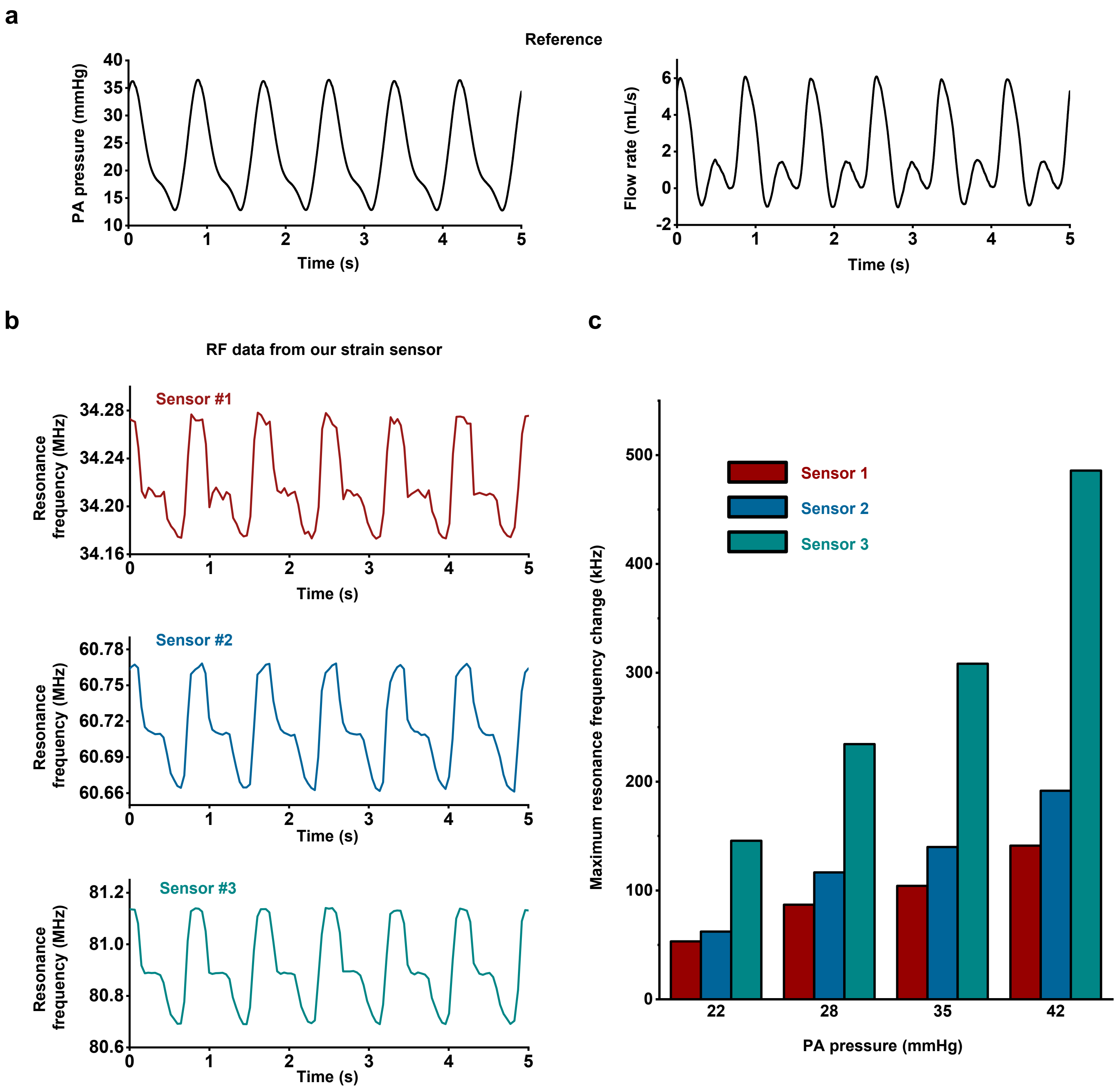


**Supplementary Figure 41 | *Ex vivo* evaluations of the cardiac device on an artificial heart system with adjustable oscillatory flow and pressure. a,** PA pressure and flow rate as references. **b,** Resonance frequency data for each sensing unit. The results correspond well to the reference pressure and flow rate data in terms of waveform shape and frequency. **c,** Maximum change in resonance frequency increases as the PA pressure rises in these three sensing units.

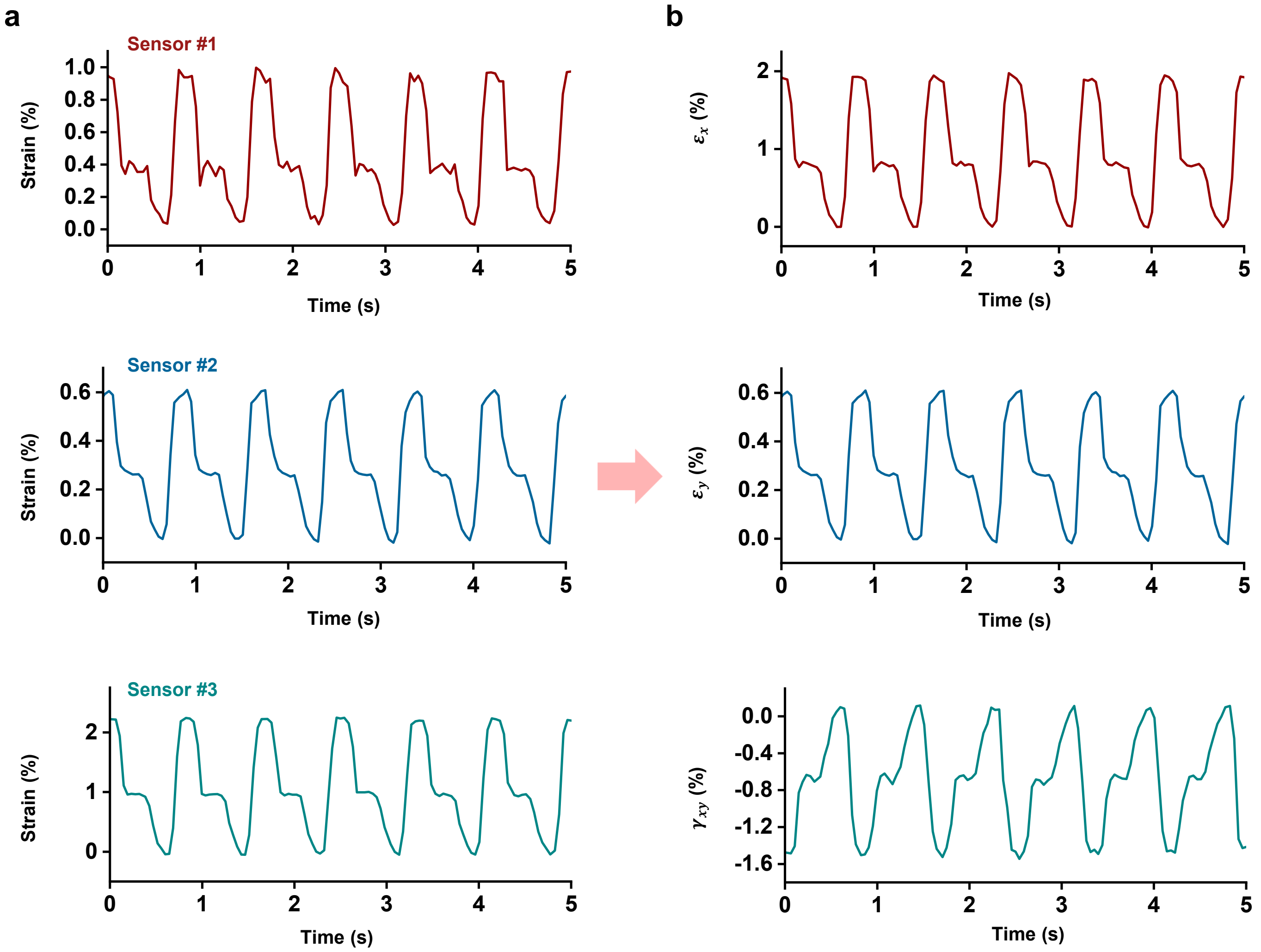


**Supplementary Figure 42 | Principal and shear strains in *ex vivo* evaluations of the cardiac device. a,** Converted strain data along with each sensing unit. **b,** Coordinate transformation of the strain values along the three arms determines the principal and shear strains.

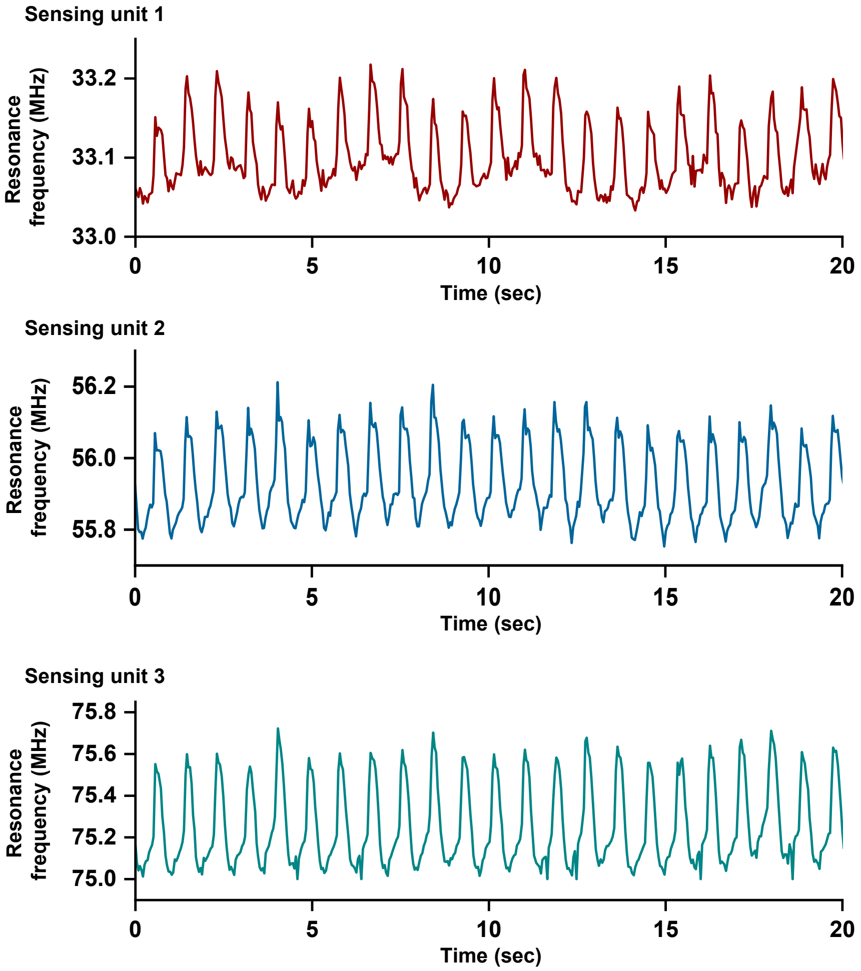


**Supplementary Figure 43 | Raw data for the three units in a representative segment (duration: 20 s).**

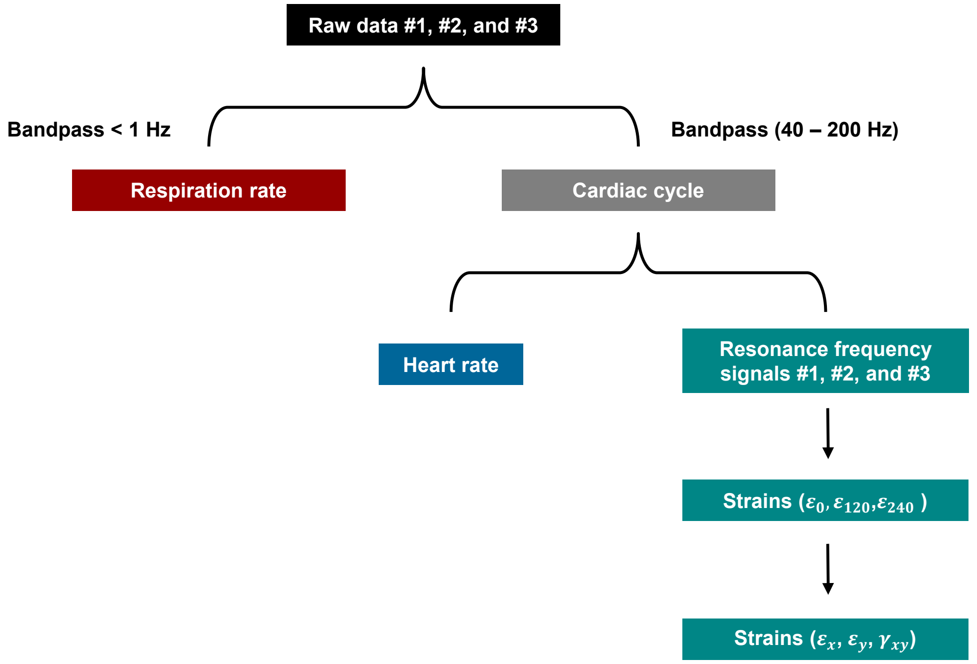


**Supplementary Figure 44 | Data filtering for raw data measured from the LV of the porcine heart.** Data with a bandpass of < 1 Hz determines the respiration rate, and data with a bandpass of 40 – 200 Hz determines the heart rate and the strains.

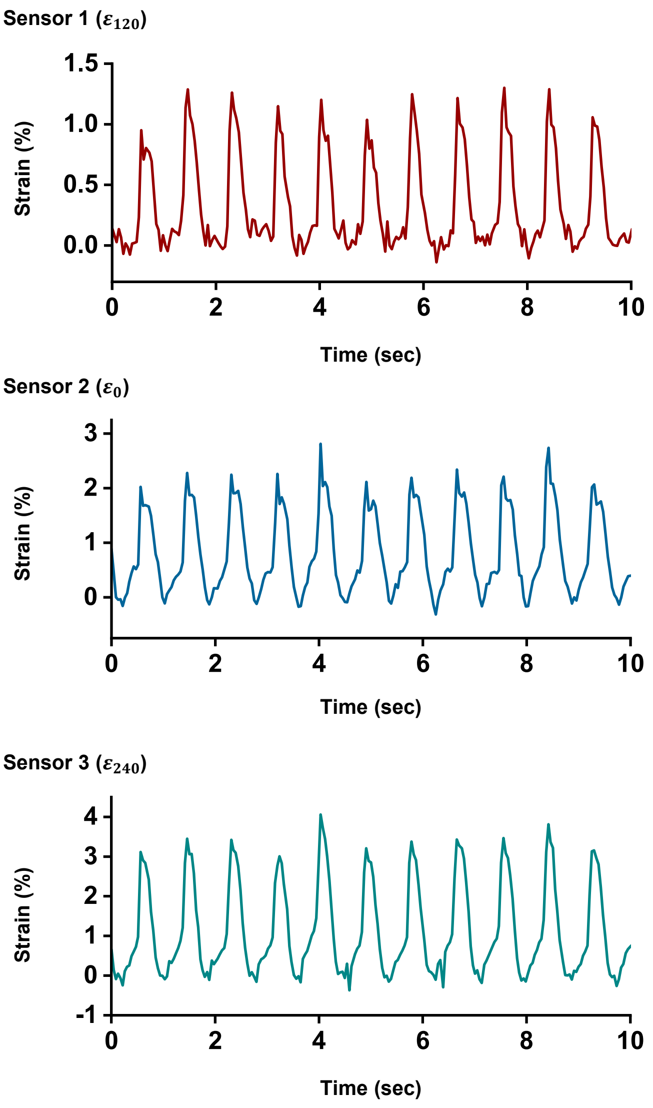


**Supplementary Figure 45 | Quantitative assessment of the strains of the ovine LV along the three arms, corresponding to Fig. 6j-l.**

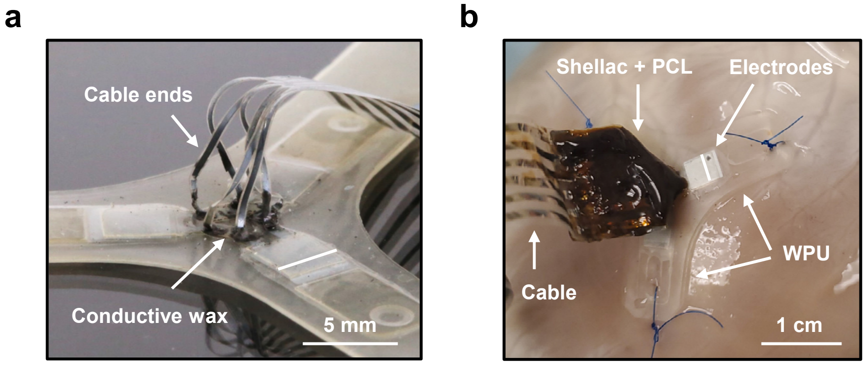


**Supplementary Figure 46 | Connection between the multi-sensing element and the flexible cable. a,** Bioresorbable conductive wax connects the ends of the six independent traces in the flexible cable to the six pads of the three pairs of bottom electrodes in the multi-sensing element. **b,** A bioresorbable polymer mixture based on PCL and shellac (ratio of PCL to shellac: 9:1) seals the connection sites. The PCL provides encapsulation and the shellac provides adhesion.

## References


1. Hwang, S.-W. *et al.* High-performance biodegradable/transient electronics on biodegradable polymers. *Advanced Materials* **26**, 3905–3911 (2014).
2. Yang, Q. *et al.* Materials, mechanics designs, and bioresorbable multisensor platforms for pressure monitoring in the intracranial space. *Advanced Functional Materials* **30**, 1910718 (2020).
3. Kang, S.-K. *et al.* Bioresorbable silicon electronic sensors for the brain. *Nature* **530**, 71–76 (2016).
4. Yu, X., Shou, W., Mahajan, B. K., Huang, X. & Pan, H. Materials, processes, and facile manufacturing for bioresorbable electronics: a review. *Advanced Materials* **30**, 1707624 (2018).
5. Shou, W. *et al.* Low-cost manufacturing of bioresorbable conductors by evaporation–condensation-mediated laser printing and sintering of Zn nanoparticles. *Advanced Materials* **29**, 1700172 (2017).
6. Lee, Y. K. *et al.* Room temperature electrochemical sintering of Zn microparticles and its use in printable conducting inks for bioresorbable electronics. *Advanced Materials* **29**, 1702665 (2017).
7. Han, W. B., Ko, G.-J., Shin, J.-W. & Hwang, S.-W. Advanced manufacturing for transient electronics. *MRS Bulletin* **45**, 113–120 (2020).
8. Feng, S., Cao, S., Tian, Z., Zhu, H. & Kong, D. Maskless patterning of biodegradable conductors by selective laser sintering of microparticle inks and its application in flexible transient electronics. *ACS Applied Materials & Interfaces* **11**, 45844–45852 (2019).
9. Li, J., Luo, S., Liu, J., Xu, H. & Huang, X. Processing techniques for bioresorbable nanoparticles in fabricating flexible conductive interconnects. *Materials* **11**, 1102 (2018).
10. Feng, S., Tian, Z., Wang, J., Cao, S. & Kong, D. Laser sintering of Zn microparticles and its application in printable biodegradable electronics. *Advanced Electronic Materials* **5**, 1800693 (2019).
11. Mahajan, B. K. *et al.* Aerosol printing and photonic sintering of bioresorbable zinc nanoparticle ink for transient electronics manufacturing. *Science China Information Sciences* **61**, 060412 (2018).
12. Huang, X. *et al.* Biodegradable materials for multilayer transient printed circuit boards. *Advanced Materials* **26**, 7371–7377 (2014).
13. Xian, J. *et al.* A simple model to predict machined depth and surface profile for picosecond laser surface texturing. *Applied Sciences* **8**, 2111 (2018).
14. Valvano, J. W., Allen, J. T. & Bowman, H. F. The simultaneous measurement of thermal conductivity, thermal diffusivity, and perfusion in small volumes of tissue. *Journal of Biomechanical Engineering* **106**, 192–197 (1984).
15. Khot, M. B. *et al.* Thermal diffusion probe analysis of perfusion changes in vascular occlusions of rabbit pedicle flaps. *Plastic and Reconstructive Surgery* **115**, 1103–1109 (2005).
